# Spike‐Embedded Nanocatalysts via Metal‐Directed Carbonization for Highly Efficient and Robust Semi‐Hydrogenation

**DOI:** 10.1002/advs.75570

**Published:** 2026-05-14

**Authors:** Yintao Li, Yang Sun, Minghang Li, Wenxuan Zhang, Zhengtao Li, Yihao Ni, Yao Zhang, Yong Wang, Samuel S. Veroneau, Pengfei Ji

**Affiliations:** ^1^ Institute of Catalysis, Department of Chemistry Zhejiang University Hangzhou P. R. China; ^2^ Zhejiang Key Laboratory of Low‐Carbon Synthesis of Value‐Added Chemicals Zhejiang University Hangzhou P. R. China; ^3^ Institute of Science Westlake Institute for Advanced Study Hangzhou P. R. China; ^4^ Department of Chemistry Westlake University Hangzhou P. R. China; ^5^ Vagelos Institute for Energy Science and Technology University of Pennsylvania Philadelphia Pennsylvania USA

**Keywords:** anti‐poisoning, azoxybenzene, carbon material, palladium, semi‐hydrogenation

## Abstract

Achieving high catalytic efficiency while maintaining robust structural stability is a persistent dilemma in the design of carbon‐supported nanocatalysts. Herein, we report a metal‐directed carbonization strategy to resolve this trade‐off by utilizing hierarchical organic microspheres as precursors. The pivotal role of metal ions (e.g., Pd^2+^) extends beyond serving as precursors for metallic active sites, as they also facilitate carbonization at reduced temperatures and help preserve the precursor morphology during calcination. This metal‐assisted aromatic coupling process reduces carbonization temperature by 130°C and drives the migration of metal nanoparticles to the tips of the carbon spikes, where they become embedded within the carbon matrix and partially exposed. Consequently, the afforded catalyst is 84 times more active than Pd/C and 14 times more active than Pd/Al_2_O_3_ for semi‐hydrogenation of nitrobenzene to azoxybenzene, and maintains high activity and selectivity even in the presence of ethylenediamine and thiourea. The catalyst also displays applicability in the semi‐hydrogenation of alkynes to alkenes with excellent chemoselectivity. Overall, the templated strategy is general, extends to multiple metals and microsphere morphologies, and provides a scalable route to carbon‐supported catalysts that combine site accessibility with nanoparticle stabilization for challenging, poison‐prone reactions.

## Graphical abstract

A metal‐directed carbonization strategy transforms hierarchical organic microspheres into spiky carbon catalysts with Pd nanoparticles embedded at the tips. This approach resolves the activity‐stability trade‐off, achieving exceptional poison resistance in semi‐hydrogenations, offering a versatile, scalable platform for designing high‐performance, robust carbon‐supported nanocatalysts.

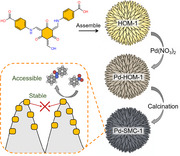

## 1 Introduction

The precise spatial confinement of metal nanoparticles within porous supports represents a challenge in heterogeneous catalysis, aiming to maximize atomic utilization while mitigating deactivation pathways such as sintering and leaching. Carbon materials, owing to their tunable electronic properties and chemical inertness, serve as widely used supports [1, 2, 3, 4]. However, their synthetic protocols often face an inherent trade‐off between active site accessibility and structural stability. Conventional synthetic methods, such as polymer carbonization or biomass pyrolysis [5, 6], typically offer limited control over the morphology and pore structure of carbon supports, affording poorly structured catalysts with active sites either buried deep within the carbon matrix, resulting in limited mass transport and catalytic efficiency [7, 8, 9, 10, 11].

Templated synthesis is a promising strategy to produce carbon materials with controlled nanostructures [12, 13, 14, 15, 16, 17]. For example, flower‐like particles include pores that enhance surface accessibility, ion diffusion, and mass transport, demonstrating the power of templated synthesis in accessing more robust and useful types of carbon materials [18, 19, 20]. While templated synthesis can create open carbon frameworks to enhance mass transport and surface accessibility, traditional techniques for immobilizing nanocatalysts onto carbon supports, such as physical adsorption or surface coordination [21, 22], fail to prevent the migration and sintering of nanoparticles [23, 24]. This issue becomes especially serious when strongly coordinating catalyst poisons (e.g., amines, thiols) are present, which competitively adsorb onto active sites, rendering conventional surface‐loaded catalysts inactive [25]. Strategies that can spatially isolate metal nanoparticles to prevent sintering while still maintaining a high accessibility for substrates are essential for broadening the application scope of durable catalysis in harsh chemical environments.

**SCHEME 1:**
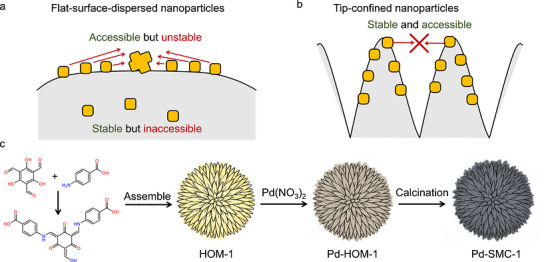
Design and synthesis of spiky microspherical carbons. (a) Schematic illustration for the Ostwald ripening process of surface‐supported metal nanoparticles. (b) Schematic illustration for spatial isolation of metal nanoparticles by tip enrichment. (c) Schematic illustration for the synthesis of microspherical carbons.

Herein, we present a metal‐directed carbonization strategy to construct hierarchically structured, spiky microspherical carbon (SMC) supports that simultaneously achieve high structural stability and high accessibility of active sites [26, 27]. By utilizing hierarchical organic microspheres as precursors, we demonstrate that palladium ions play a synergistic dual role, stabilizing the precursor assembly through coordination while catalyzing the carbonization process at temperatures reduced by approximately 130°C. Also, the sintering of the carbon matrix drives Pd nanoparticles toward the surface and concentrates them at the spike tips, while the simultaneous densification of the outer carbon layer embeds the particles so that they are partially exposed at the surface. This hierarchical structure not only prevents nanoparticle further migration and aggregation but also exposes catalytically active sites at accessible spike tips, offering clear structural advantages over conventional carbon supports (Scheme 1a, b) [28, 29]. Moreover, the hierarchical structure imparts superhydrophobicity akin to lotus leaves, which modulates the binding affinity of competing reactants and intermediates in multi‐step reactions. This work provides a general approach for preparing carbon‐supported catalysts from hierarchical small‐molecule assemblies and demonstrates their application in hydrogenation reactions.

## 2 Results and Discussion

### 2.1 Characterization of Pd‐SMC‐1

HOM‐1 was synthesized via imine condensation of 2,4,6‐trihydroxybenzene‐1,3,5‐tricarbaldehyde and 4‐aminobenzoic acid, during which the double‐condensation of the precursors triggers spontaneous precipitation and in situ assembly, forming microspheres with intertwined fibers on the surface(Figure S1a) [26]. This material exhibited a negative zeta potential of −30.1 ± 6.7 mV, promoting the electrostatic loading of cationic Pd^2+^. After mixing with the Pd(NO_3_)_2_ solution, the material changed color from yellow to brown, accompanied by the appearance of a new UV–vis absorption band at ∼440 nm, which is a spectroscopic characteristic of Pd^2+^ ions, confirming the successful formation of Pd‐HOM‐1 (Figure S1b,c) [30]. Energy‐dispersive X‐ray spectroscopy (EDS) analysis confirmed Pd incorporation and uniform distribution through its diagnostic Lα edge at 2.835 keV (Figures S2 and S3). Transmission electron microscopy (TEM) of ultrathin sectioned Pd‐HOM‐1 samples revealed no visible nanoparticles, while inductively coupled plasma‐mass spectrometry (ICP‐MS) analysis quantified the Pd loading of 12.7 wt.% (Figure S4). X‐ray photoelectron spectroscopy (XPS) revealed a notable difference between HOM‐1 and Pd‐HOM‐1 in the O_1s_ region before calcination. In Pd‐HOM‐1, the peak intensity at 533.3 eV was significantly reduced compared to HOM‐1, likely due to coordination between Pd^2+^ and carboxyl groups (Figures S5 and S6) [31].

**FIGURE 1:**
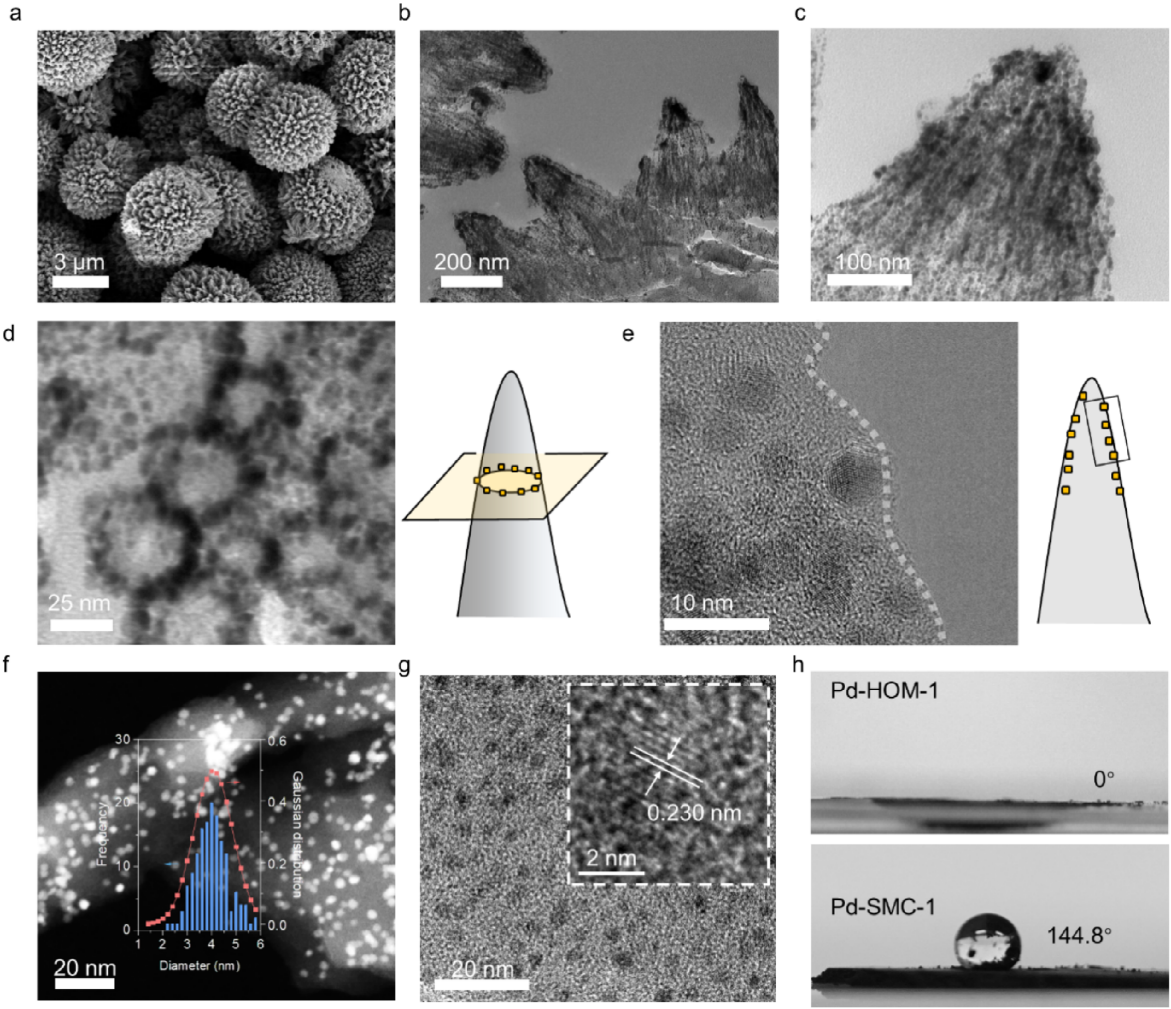
Characterizations of Pd‐SMC‐1. (a) SEM image of the Pd‐SMC‐1 powder. (b) TEM of the ultrathin section of Pd‐SMC‐1. (c) Enlarged cross‐sectional view of the tip of Pd‐SMC‐1. (d) Cross‐sectional view revealing surface‐enriched Pd nanoparticles in ring‐like arrangements. (e) HR‐TEM of spike edge demonstrating carbon‐encapsulated Pd nanoparticles. Insets: illustration of the imaging locations. (f) HADDF‐STEM image of ultrathin section of Pd‐SMC‐1. The insert chart is the size distribution of Pd nanoparticles within Pd‐SMC‐1. (g) HR‐TEM image of Pd nanoparticles within Pd‐SMC‐1. The inserted chart is the enlarged image of the lattice fringe. (h) Water contact angle over Pd‐HOM‐1 and Pd‐SMC‐1.

The high loading and uniform distribution of Pd^2+^ made Pd‐HOM‐1 an ideal platform for producing carbon embedded with Pd‐nanoparticles through pyrolysis. Based on thermogravimetric analysis, the material reached a stable mass above 450°C (Figure S7). Accordingly, Pd‐HOM‐1 was calcined under N2 at 450°C for 2 h, affording a black powder in 50% mass yield (Scheme 1c). Scanning electron microscopy (SEM) revealed that the spiky morphology of HOM‐1 and Pd‐HOM‐1 was preserved in this carbonized material (Figure 1a and Figure S8). Delaunay triangulation analysis (Figure S9) further qualified this surface morphology with a point spacing of 0.43 ± 0.12 µm, almost identical to the original HOM‐1 (0.48 ± 0.14 µm). The carbonized Pd‐SMC‐1 also successfully retained this mesoporous architecture with a surface area of 67 m2/g (Figure S10). In contrast, direct calcination of HOM‐1 under the same conditions without Pd^2+^ showed a collapse of surface structure, suggesting a stabilizing role of Pd^2+^ during carbonization (Figure S11). The particle size distribution after calcination was analyzed using static light scattering and determined to be 6.8 ± 3.4 µm, which was varied insignificantly from the particle size of HOM‐1 (6.4 ± 2.0 µm) and Pd‐HOM‐1 (7.1 ± 3.2 µm; Figure S12). EDS elemental mapping confirmed the uniform distribution of Pd, and the Pd content was determined to be 39.4 wt % by ICP‐MS (Figures S13 and S14).

To investigate the structure and location of the embedded Pd species in the carbonized material, 100 nm thin sections were prepared via ultramicrotomy for TEM imaging (Figure S15). TEM revealed Pd nanoparticles, identified by their strong electron scattering contrast, that were enriched at the tips of the spiky microspheres (Figure 1). Cross‐sectional imaging of an individual spike showed a ring‐like distribution of nanoparticles around the spike, which is just embedded beneath the surface carbon layers (Figure 1d,e). High‐angle annular dark‐field scanning TEM (HAADF‐STEM) established a narrow size distribution of Pd nanoparticles with a diameter of 4.1 ± 0.8 nm (Figure 1f). High resolution TEM (HR‐TEM) resolved the characteristic (111) lattice fringes of metallic Pd with a d‐spacing of 0.230 nm, consistent with standard values within a face‐centered cubic cell (0.225 nm, PDF no. 46–1043; Figure 1g).

**FIGURE 2:**
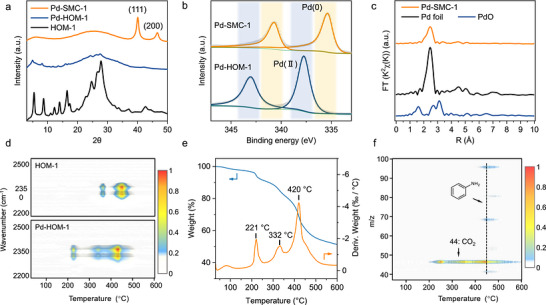
Formation mechanism of Pd‐SMC‐1 and comparison with precursor materials. (a) PXRD patterns of Pd‐SMC‐1 compared with its precursor materials. (b) XPS spectra of Pd 3d specimens for Pd‐SMC‐1 compared with Pd‐HOM‐1. (c) Fourier‐transform EXAFS spectra for Pd‐SMC‐1 at Pd K‐edge EXAFS, compared with Pd foil and palladium oxide. (d) TG‐FTIR spectra of thermal degradation of HOM‐1 and Pd‐HOM‐1. (e) The TG and the first derivative curve for Pd‐HOM‐1 during calcination. (f) TG‐MS spectra of thermal degradation of Pd‐HOM‐1.

Calcination significantly reformed the surface chemistry of the material, notably with a significant transition from a superhydrophilic (0° for Pd‐HOM‐1) to a superhydrophobic surface (144.8° for Pd‐SMC‐1) as well as a reversal of the zeta potential from −31.5 ± 2.5 to 30.6 ± 4.8 mV (Figure 1h). These surface modifications likely enhance the material's ability to selectively adsorb and activate aromatic substrates, which always play a critical role in improving catalytic performance by increasing the interaction between the catalyst and hydrophobic reactants. The structural evolution of this carbon support was further analyzed by Powder X‐ray diffraction (PXRD) and Raman spectroscopy. The PXRD patterns of HOM‐1 before and after adsorption of Pd revealed that the introduction of metal ions significantly reduced the crystallinity of the organic molecule stacking, as evidenced by the almost negligible diffraction signal of the original HOM‐1 (Figure 2a). XPS revealed a substantial reduction in N and O contents in the calcinated sample, suggesting the decomposition of the organic linker to the carbon matrix. Prior to calcination, Pd was uniformly dispersed in the material in its ionic form, and thus, no characteristic peaks of Pd species were observed. After calcination, the PXRD pattern of the sample showed distinct signals at 40.4° and 46.5°, corresponding to the (111) and (200) crystal planes of Pd (face‐centered cubic), confirming the formation of crystalline metallic Pd within the carbon matrix. While no distinctive Raman peaks were observed for the sample before calcination, after calcination, the Raman spectrum displayed the characteristic D band (1367 cm^−1^) and G band (1593 cm^−1^) of carbon material, indicating the formation of defective sp^2^ carbon with a moderate disorder level (I_D_/I_G_ = 0.82; Figure S16) [32, 33, 34]. Furthermore, solid‐state ^13^C NMR verifies the successful transformation into a graphitized, sp^2^‐hybridized carbon matrix (Figure S17) [35, 36, 37]. Based on its spiky microspherical morphology, this material was thus designated as Pd‐loaded spiky microspherical carbon (Pd‐SMC‐1).

The electronic properties of Pd were investigated using XPS to assess Pd reduction (Figure 2b). In Pd‐HOM‐1, the Pd 3d_5/2_ and 3d_3/2_ peaks appeared at 337.8 and 343.0 eV, respectively, characteristic of Pd^2+^ species [34, 38]. In contrast, these peaks shifted to 335.4 and 340.8 eV in Pd‐SMC‐1, which are consistent with metallic Pd^0^. Synchrotron radiation X‐ray absorption spectroscopy corroborated this. EXAFS fitting showed intermetallic Pd─Pd bonding interactions (2.75 Å) as the dominant first‐shell coordination, with no Pd─O contributions detected (Figure 2c and Figure S18). Quantitative fitting reveals a significantly reduced Pd─Pd coordination number of 5.7 for Pd‐SMC‐1, which indicates the presence of atomically dispersed Pd species or ultra‐small clusters stabilized within the carbon matrix [39, 40, 41].

### 2.2 Formation of Pd‐SMC‐1 Through Pd‐Directed Carbonization

The presence of Pd^2+^ extends beyond serving as precursors for the potential catalytic sites, which also significantly alters the carbonization kinetics by catalyzing decomposition at lower temperatures. Thermogravimetric (TG) analysis indicates that Pd‐HOM‐1 begins to decompose at approximately 200°C, accompanied by a visible color change to black by 220°C, whereas the metal‐free HOM‐1 remains stable until 330°C (Figure S19). SEM and TEM images confirmed that Pd‐HOM‐1 preserved its fibrous structure on the surface at high temperatures, whereas the surface of HOM‐1 transformed into disordered structures due to thermal instability (Figure S20 and S21). The calcinated HOM‐1, namely, SMC‐1, exhibited numerous voids within the carbon framework (Figure S22), indicative of structural instability during thermal decomposition. DSC confirms that the metal‐free precursor lacks a melting phase prior to 300°C, proving the structural instability is not caused by melting (Figure S26).

TG combined with Fourier transform infrared spectroscopy (FTIR) and mass spectrometry (MS) shows the detailed pyrolysis mechanisms for HOM‐1 and Pd‐HOM‐1. These weight losses are primarily attributed to decarboxylation, as evidenced by the release of CO_2_ with characteristic signals at 2383 and 2350 cm^−1^ (Figure 2d). Pristine HOM‐1 exhibits two main weight‐loss events above 300°C with an initial decarboxylation at 351°C (6.5 wt.%, equal to 0.7 CO_2_ per monomer) and a major decomposition at 435°C (39 wt.%, 3.3 CO_2_ and 0.3 aniline per monomer), leaving a char yield of about 54.4% (Figure S23). By contrast, Pd‐HOM‐1 follows a modified pathway with three decarboxylation steps (Figure 2e,f), including 221°C (7.2 wt.%, 0.8 CO_2_), 332°C (11 wt.%, 1.3 CO_2_), and a final step at 420°C (27 wt.%, 1.9 CO_2_ together with 0.8 aniline).TG‐FTIR of Pd‐HOM‐1 also shows an additional band around 1620 cm^−1^ attributable to NO_2_ from residual nitrate (Figure S24). Variable‐temperature FTIR dynamically captures this degradation, revealing that the gradual attenuation of C═O and C═N stretching vibrations (1600–1700 cm^−1^) aligns well with these sequential decarboxylation steps (Figure S25). Mechanistically, Pd^2+^ ions effectively activate the adjacent carboxylate groups, significantly lowering the activation energy for C─C bond cleavage and facilitating the extrusion of CO_2_ under much milder conditions [42, 43]. These results confirm that Pd mediates sequential decarboxylation steps and promotes efficient carbonization under much milder conditions. Simultaneously, Pd reduces the molecular mobility of the organic precursor through strong coordination interactions, preserving the fibrous morphology after calcination. This dual role of Pd provides a method for preparing carbon‐supported nanocatalysts with specific surficial morphology.

### 2.3 Generalizable Synthetic Strategy and Morphological Diversity of MC Materials

**FIGURE 3:**
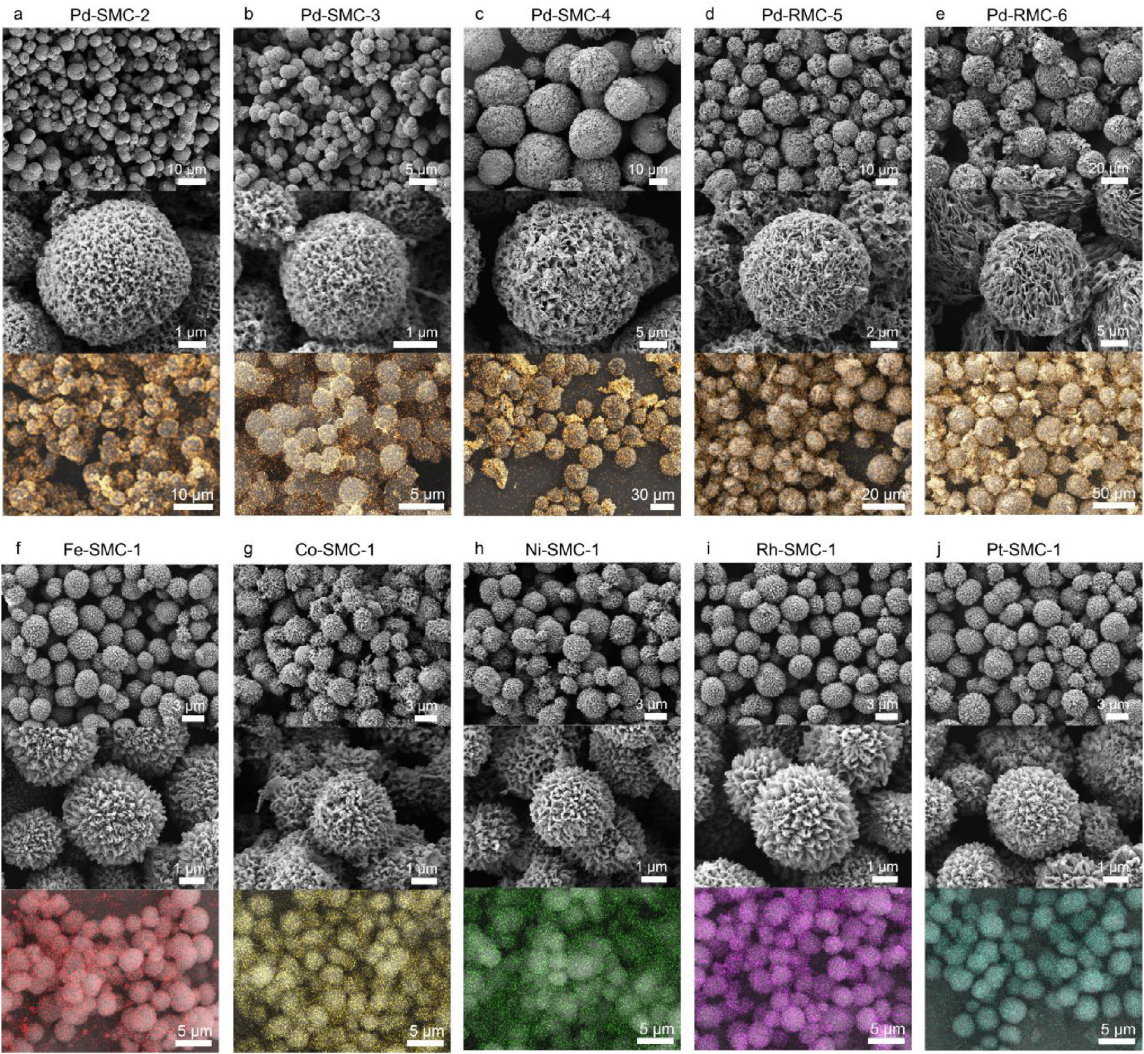
Diversity of MCs derived from diverse HOMs and metallic addition. (a–e) SEM images (top and middle) for Pd‐MCs derived from diverse HOMs; the bottom images are the EDS mapping images of Pd‐MCs. (f‐j) SEM (top and middle) images for the various SMC‐1 loaded with diverse metal ions; the bottom images are the EDS mapping images of M‐SMC‐1. (orange: Pd; red: Fe; yellow: Co; green: Ni; purple: Rh; cyan: Pt).

We extended our synthetic method to prepare 10 other nanocatalysts supported on microspherical carbons with diverse morphologies and metal types (Figure 3 and Figure S27). By altering the structure of the assembly monomers, HOMs with different surface morphologies can be obtained. Delaunay triangulation analysis quantitatively confirms that these structural features are well‐preserved post‐calcination (Figure S28a). We further demonstrated that each of these HOMs can be converted into the corresponding MCs. Materials with ridge morphologies are denoted as ridged microspherical carbon (RMC). The successful carbonization of organic precursors in all samples was confirmed by Raman spectroscopy, showing characteristic D (1367 cm^−1^) and G (1593 cm^−1^) bands (Figure S29). EDS mapping showed uniform Pd dispersion across all Pd‐MCs with varying metal loadings from 20 to 46 wt.%. Thermal stability tests in air showed that all Pd‐MCs maintained structural integrity up to 300°C with minimal weight loss (Figure S30).

The Pd‐MCs exhibited varied microsphere diameters. Although Pd‐SMC‐3 is only about 30% the size of Pd‐SMC‐1 (2.0 ± 0.4 µm), it maintains a similar Pd content of 31% while hosting much smaller Pd nanoparticles (2.6 ± 0.5 nm). Regardless of size, all spiky variants maintained a surface‐enriched Pd distribution (Figures S31 and S32). Similarly, Pd‐RMCs were prepared with ridged surface morphology, characterized by distinct linear protrusions (e.g., Figure 3e), though with larger particle sizes. These materials in different morphologies showed surface‐enriched Pd nanoparticle distribution. Pd‐RMC‐6 had an average particle size of 25 ± 3.0 µm, with Pd accumulation at the top and bottom surfaces and limited incorporation into the interior (Figure S33).

This synthetic strategy was further extended to include other metals commonly used in hydrogenation (Pt, Rh) and redox‐active transition metals (Fe, Co, Ni) relevant to fine chemical synthesis. All materials preserved their original morphology after calcination, as confirmed by SEM and Delaunay triangulation analysis (Figure S28b), while EDS mapping confirmed the successful incorporation of each metal (Figures S34–S38). In summary, our synthetic platform demonstrated precise control over microsphere size, metal distribution, and chemical composition, highlighting its robustness and versatility. With this method, we thus sought to employ microspherical carbons in catalysis.

To investigate the catalytic behavior at a loading closer to industrial standards, we synthesized a low‐loading catalyst denoted as Pd_0.1_‐SMC‐1 by reducing the initial palladium precursor input to one‐tenth, which successfully yielded an actual palladium content of approximately 4 wt.%. Ultrathin section TEM analysis reveals that the palladium nanoparticles in Pd_0.1_‐SMC‐1 possess an average size of 2.5 ± 0.4 nm (Figure S39a), which is slightly smaller than those in the original Pd‐SMC‐1. This reduced nanoparticle size is further corroborated by the noticeably weaker and broader diffraction peaks in the corresponding PXRD pattern (Figure S39b). Also, to investigate the importance of hierarchical structure on nanoparticle distribution, we synthesized a reference sample by modifying the solvent system and obtained a fibrous assembly (FA). Despite sharing the same primary stacking pattern as confirmed by PXRD, FA displays an entangled fibrous morphology without hierarchical assembly, as revealed by SEM imaging (Figure S40). After calcination, the Pd‐loaded fibrous network sintered to form a bulk amorphous carbon (denoted as Pd‐BAC). TEM imaging of ultrathin sections showed that Pd nanoparticles have a broad size distribution (2.4 ± 0.9 nm) at relatively low Pd loading (21%) and lack apparent surface enrichment (Figure S41). The internal embedding of Pd nanoparticles would restrict substrate access to active sites, thereby diminishing catalytic efficiency (vide infra).

### 2.4 Poison‐Resistant and Durable Catalysis for Selective Hydrogenation of Nitrobenzene

**FIGURE 4:**
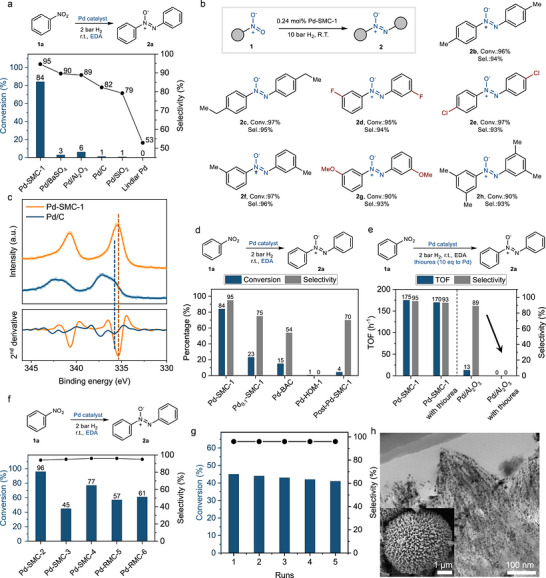
Poison‐resistance of the Pd‐SMC‐1 in nitrobenzene hydrogenation. (a) Comparison of nitrobenzene hydrogenation in EDA over diverse Pd catalysts. Reaction condition: 400 µL of EDA, 25°C, 2 h, 2 bar H_2_, 15 µL nitrobenzene, 0.24 mol% Pd. (b) Substrate scope for selective hydrogenation of nitrobenzene in EDA. (c) XPS spectra of Pd 3d specimens for Pd‐SMC‐1 compared with commercial Pd/C. The bottom is the second‐derivative spectra. (d) Effect of Pd catalyst morphology and synthetic method on nitrobenzene hydrogenation. (e) Comparison of the catalytic performance of the Pd catalyst with toxicant (thiourea). (f) Comparison of nitrobenzene hydrogenation over diverse Pd‐MCs. Reaction condition: 400 µL of EDA, 25°C, 2 h, 2 bar H_2_, 15 µL nitrobenzene, 0.24 mol% Pd. (g) Conversion of nitrobenzene and selectivity of azoxybenzene in 5 consecutive runs of Pd‐SMC‐1 catalyzed reactions. Reaction conditions: 500 µL of EDA, 30 µL of substrate, 0.15 mg of the catalyst, 25°C, 2 bar H_2_, 2 h. (h) TEM images of Pd nanoparticles within Pd‐SMC‐1 after 5 cycles of reaction. The inserted figure is a SEM image of the sample.

Azoxybenzenes are valuable intermediates in the synthesis of dyes, pharmaceuticals, and functional materials, and their controlled preparation from nitroaromatics remains an attractive route in green chemistry. Traditionally, their selective synthesis from nitroaromatics often relies on the use of alkali, like ethylenediamine (EDA), to modulate the hydrogenation pathway [44]. In this process, the alkaline nature of EDA enhances the condensation of the hydroxylamine intermediate (Figure S42) [45]. However, utilizing EDA as a solvent presents a significant challenge, as such strongly coordinating amines typically deactivate metal catalysts. Notably, Pd‐SMC‐1 demonstrated exceptional resistance to this poisoning effect. In the presence of EDA, Pd‐SMC‐1 achieved the highest performance among all tested catalysts, reaching 84% conversion with 95% selectivity toward azoxybenzene within 2 h (Figure 4a and Figure S43). By contrast, other representative Pd catalysts exhibited negligible reactivity due to severe poisoning. The next best performer, Pd/Al_2_O_3_, converted only 6% of the nitrobenzene, highlighting the superior activity of Pd‐SMC‐1 in this challenging environment. The general applicability of this system was further evaluated. Substrates bearing methyl, ethyl, fluorine, and methoxy groups all exhibited >93% selectivity for azoxybenzenes with >90% conversion (Figure 4b; Figures S44–S50).

The enhanced performance and poison resistance of Pd‐SMC‐1 stem from a combination of electronic and geometric properties. XPS shows that the Pd 3d_5/2_ peak in Pd‐SMC‐1 is at a lower binding energy by approximately 0.5 eV relative to Pd/C, a difference that is clearer in second‐derivative spectra and indicates an intrinsically more electron‐rich nature of Pd species (Figure 4c) [46, 47]. Upon EDA introduction, the Pd 3d_5/2_ peak in Pd/C shifts significantly toward lower binding energy (∼0.4 eV), evidencing strong amine coordination and consequent poisoning. Conversely, Pd‐SMC‐1 exhibits a minimal shift of only ∼0.1 eV under identical conditions (Figure S51). This stark difference confirms that the electron‐rich nature of Pd‐SMC‐1 effectively mitigates strong EDA adsorption, preserving the active sites for catalysis and enhancing the adsorption of electron‐poor nitro groups, improving substrate enrichment at active sites. Moreover, contact angle measurements (25° for pure EDA on Pd‐SMC‐1) confirm that EDA readily wets the surface, proving that the outstanding poison resistance is primarily driven by this intrinsic electronic effect rather than physical exclusion by the hydrophobic carbon shell (Figure S52).

Furthermore, compared to Pd‐SMC‐1, Pd_0.1_‐SMC‐1 exhibited a significantly lower catalytic efficiency with 23% conversion. This likely originates from the fact that at drastically reduced metal loadings, a larger fraction of the ultra‐small nanoparticles becomes deeply encapsulated within the vast rigid carbon framework rather than optimally exposed on the accessible spiky surfaces (Figure 4d). Structurally, the non‐hierarchical Pd‐BAC underwent pronounced sintering during calcination, which led to a substantial loss of accessible catalytic sites and consequently lower activity compared to Pd‐SMC‐1 (Figure 4d). The inhomogeneous distribution of Pd nanoparticles within Pd‐BAC further compromised reaction selectivity.

In the case of Pd‐HOM‐1 without calcination, the Pd species were not stabilized by a carbon framework. During catalysis, partial dissolution of the material occurred, followed by Pd leaching and aggregation into large particles, resulting in negligible catalytic activity. TEM imaging of the sample after catalysis showed large macroscopic Pd nanoparticles, which were likely formed from the reduction and aggregation of leached Pd^2+^ species in the absence of structural confinement (Figure S53). These results highlight the importance of maintaining well‐dispersed Pd nanoparticles under reaction conditions. In addition, post‐Pd‐SMC‐1 was synthesized by a conventional post‐impregnation route, in which SMC‐1 first adsorbed Pd ions, followed by NaBH_4_ reduction to generate Pd nanoparticles. Because the Pd nanoparticles in this material were not embedded and stabilized by the carbon matrix, it exhibited deactivation in EDA, similar to that of commercial Pd/C.

Moreover, sulfur‐containing compounds are well‐known catalyst poisons in hydrogenation reactions [48]. Upon introduction of 10 equivalents of thiourea, Pd/Al_2_O_3_ was completely deactivated, whereas Pd‐SMC‐1 retained high activity against both thiourea and thiophene, further highlighting the superior anti‐poisoning capability of the Pd‐MC catalysts (Figure 4e and Figure S54). We further confirmed the robustness and generality of our synthesis strategy (Figure 4f). All Pd‐MCs maintained conversion rates above 45% with high selectivity, demonstrating that stabilization of Pd nanoparticles by an in situ‐generated carbon matrix is a generally effective strategy for enhancing poison resistance.

The catalyst also demonstrated excellent durability. Hot filtration control experiments completely ruled out contributions from homogeneous catalytic species (Figure S55), and ICP‐MS measurements of the post‐reaction filtrate detected low Pd leaching (∼1.8%). Over five consecutive reaction cycles, Pd‐SMC‐1 maintained a steady conversion of approximately 43% while preserving a high selectivity of 96%, achieving a cumulative turnover number of over 900 (Figure 4g). Post‐reaction characterization confirmed the structural integrity of the catalyst, revealing well‐preserved microspherical morphology and no aggregation of the Pd nanoparticles (4.0 ± 1.0 nm; Figure 4h and Figure S56). Furthermore, XPS of post‐reaction catalyst revealed a marginal binding energy shift of ∼0.1 eV compared to newly synthesized Pd‐SMC‐1, which is identical to the shift observed in the simple EDA‐treated control sample, demonstrating that the structural and electronic integrities were well‐preserved during the catalytic cycles (Figure S57).

Full hydrogenation of nitrobenzene to aniline could also be achieved with Pd‐SMC‐1 by changing the reaction conditions. In aqueous media, Pd‐SMC‐1 efficiently catalyzed the hydrogenation of nitroarenes to anilines, reaching 78% conversion within 12 h with no detectable byproducts. A wide scope of ten substrates was achieved with >93% selectivity, tolerating both electron‐rich substituents such as alkyl and methoxy groups and electron‐deficient groups including fluorine, cyano, and trifluoromethyl (Figure S58). Notably, the catalyst remained highly robust under solvent‐free conditions, enabling gram‐scale hydrogenation of nitrobenzene (1.8 g) to afford 1.2 g of aniline in 88% isolated yield at a low Pd‐loading of 0.05 mol% (Figure S59).

### 2.5 Semi‐Hydrogenation of Alkynes to Alkenes Catalyzed with Pd‐MCs

**FIGURE 5:**
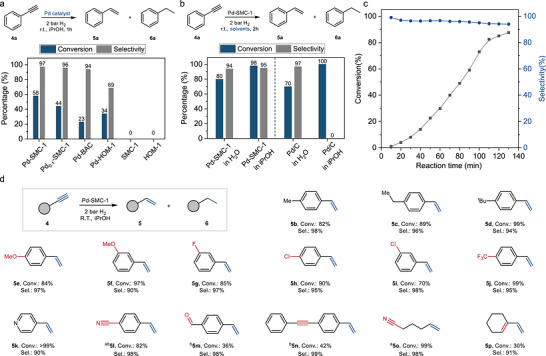
Selective catalytic hydrogenation of phenylacetylene. (a) Effect of Pd catalyst morphology and synthetic method on phenylacetylene hydrogenation. (b) Effect of reaction solvent on phenylacetylene hydrogenation. (c) The conversion of phenylacetylene and the selectivity of phenylethylene versus time over Pd‐SMC‐1. (d) Substrate scope for selective hydrogenation of alkynes by Pd‐SMC‐1. The blue color represents the hydrogenation of C≡C bonds to corresponding C═C. Reaction condition: 400 µL of iPrOH, 25°C, 2 h, 2 bar H_2_, 0.146 mmol substrate, 0.24 mol% Pd. ^a^12 h of reaction time. ^b^acetonitrile as the solvent.

Pd‐SMC‐1 also demonstrated outstanding performance in the selective semi‐hydrogenation of alkynes. For phenylacetylene, Pd‐SMC‐1 achieved over 97% selectivity toward phenylethylene at 58% conversion (Figure 5a). In contrast, the low‐loading Pd_0.1_‐SMC‐1 retained only 75% of the original activity, and Pd‐BAC showed a pronounced decrease by 60% in catalytic yield under identical conditions, highlighting the critical role of hierarchical carbon structures and optimal metal exposure in ensuring the accessibility of active sites. Similar to the hydrogenation of nitrobenzene, the lack of stability leads to the Pd‐HOM‐1 performing lower conversion (34%) and poorer selectivity (69%). Control experiments confirmed that Pd‐free materials exhibited no catalytic activity.

Notably, Pd‐SMC‐1 also exhibited superior solvent tolerance in the semi‐hydrogenation of phenylacetylene compared with conventional Pd/C. In isopropanol, an extended reaction time to 2 h led to 98% conversion while maintaining 95% selectivity toward phenylethylene, demonstrating that high activity and selectivity can be simultaneously achieved in protic organic solvents (Figure 5b). Importantly, Pd‐SMC‐1 remained effective in water, delivering 80% conversion with 94% selectivity, highlighting its robustness across solvents of markedly different polarity. In contrast, Pd/C was overly active in isopropanol, resulting in the complete hydrogenation of phenylacetylene to phenylethane. However, in aqueous media, its activity dropped significantly, reaching only 70% conversion, with phenylethylene becoming the dominant product. These results further highlight the ability of the carbon‐embedded Pd sites in Pd‐SMC‐1 to balance activity and selectivity over a broad solvent window.

The origin of the high selectivity in alkyne hydrogenation was analyzed by temperature‐programmed desorption studies. The stronger adsorption of alkynes relative to alkenes, as evidenced by higher desorption temperatures (119°C vs 103°C) for C_2_H_2_ than for C_2_H_4_, suppresses over‐hydrogenation and accounts for the observed selectivity (Figure S60). Time‐resolved catalytic profiles further revealed a rapid increase in conversion after an initial activation period, reaching about 90% conversion within 120 min while maintaining phenylethylene selectivity above 95% throughout the reaction (Figure 5c).

The versatility of Pd‐SMC‐1 was further demonstrated through its compatibility with a wide range of alkyne substrates. High activity and selectivity were maintained for substrates bearing both electron‐donating and electron‐withdrawing groups, while reducible functionalities such as cyano and carbonyl groups remained intact, underscoring the excellent chemoselectivity of the catalyst (Figure 5d and Figures S61–S75). Remarkably, Pd‐SMC‐1 enabled precise regioselective hydrogenation of multifunctional alkynes, achieving 99% selectivity for terminal alkynes while leaving internal alkynes unconverted (Figure S74). In summary, these results demonstrate that Pd‐SMC‐1 is a versatile and selective catalyst for semi‐hydrogenation with broad substrate scope and precise control over product selectivity.

## 3 Conclusion

In this study, we developed a general templated strategy to construct spiky microspherical carbon supports that confine Pd nanoparticles at highly accessible surface locations, resulting in a robust and poison‐resistant hydrogenation catalyst. The presence of Pd ions not only stabilizes the precursor assembly through strong coordination interactions but also simultaneously catalyzes the carbonization process at significantly lower temperatures. During the carbonization of the matrix, the metal nanoparticles are gradually expelled and migrate toward the surface of the carbon spikes, where they are embedded within the carbon matrix yet surface‐exposed. This unique architecture effectively suppresses nanoparticle aggregation and leaching, addressing common stability issues in supported catalysts. Also, benefiting from electron donation by the surrounding carbon matrix, the resulting electron‐rich Pd surface effectively suppresses catalyst deactivation by common amine‐ and sulfur‐containing poisons, enabling stable performance under challenging conditions. Furthermore, the catalyst showes wide applicability, including the selective hydrogenation of functionalized nitroarenes and the semi‐hydrogenation of alkynes across diverse solvent systems. The catalyst also demonstrates outstanding structural stability and recyclability. The successful application of this method across various metals and surface morphologies provides a practical structural approach for designing carbon‐supported nanocatalysts.

## 4 Experimental Section

Experimental details, including catalyst preparation, characterizations, and catalytic performance tests, are provided in the Supporting Information.

## Funding

This work was supported by the National Key R&D Program of China (No. 2025YFA0921000 and 2024YFA1510500) and NSFC (22377107).

## Conflicts of Interest

The authors declare no conflict of interest.

## Supporting information




**Supporting File**: advs75570‐sup‐0001‐SuppMat.pdf.

## Data Availability

The data that supports the findings of this study are available in the supplementary material of this article.
