# Supplementary material for: Spike‐Embedded Nanocatalysts via Metal‐Directed Carbonization for Highly Efficient and Robust Semi‐Hydrogenation
Source: Adv Sci (Weinh). 2026 May 14;13(43):e75570. doi: 10.1002/advs.75570 (PMC13335993; doi:10.1002/advs.75570)
Supplement: Supplementary file 1 — Supporting File: advs75570‐sup‐0001‐SuppMat.pdf. [file ADVS-13-e75570-s001.pdf]

Supporting Information

Spike-Embedded Nanocatalysts via Metal-Directed Carbonization for Highly Efficient and Robust Semi-Hydrogenation

*Yintao Li†, Yang Sun†, Minghang Li, Wenxuan Zhang, Zhengtao Li, Yihao Ni, Yao Zhang, Yong Wang, Samuel S. Veroneau, Pengfei Ji**

Table of content

Experimental Procedures 2
- General information 2
- Synthetic procedure of HOMs 6
- Synthesis of X-MC-1 7
- Synthesis of Reference Catalysts 7
- Surface modelling of Pd-SMC-1 8
- Catalytic performance evaluation for selective hydrogenation of nitrobenzen 11
- Catalyst recycling test 12
- Catalytic performance evaluation for selective hydrogenation of phenylacetylene 12

Supplementary Figure 14
Supplementary Code 89
Reference 94

Experimental Procedures

General information

Reagents

2,4,6-Trihydroxybenzene-1,3,5-tricarbaldehyde (97%), pyridine-2,6-dicarbaldehyde (98%), 2-hydroxyisophthalaldehyde (98%), 4-amino-3-ethylbenzoic acid (98%), 1-methylpyrrolidin-2-one (NMP), rhodium nitrate (99%), nitrobenzene (99%), 4-ethynylbenzonitrile (98%), 1-ethynyl-4-methylbenzene (98%), 1-ethynyl-4-(trifluoromethyl)benzene (99.5%), 1-ethynylcyclohex-1-ene (98%), 1-ethynyl-4-(phenylethynyl)benzene (98%), 3-chlorophenylacetylene (98%), 1-chloro-4-ethynylbenzene (98%), 4-ethynylbenzaldehyde (99%), p-nitrobenzyl methyl sulfide (99%), 1-nitro-4-(trifluoromethyl)benzene (98%), and 1-nitronaphthalene (98%) from Shanghai Bide Pharmatech Co., Ltd. Isophthalaldehyde (98%), 6-amino-2-naphthoic acid (97%), 4'-amino-[1,1'-biphenyl]-4-carboxylic acid (97%), potassium tetrachloroplatinate (II) (98%), 5-cyano-1-pentyne (96.77%), 4-ethynylpyridine (98%), 4-cyanophenylacetylene (98%), and p-nitrobenzonitrile (99.98%) were purchased from Shanghai Haohong Scientific Co., Ltd. 4-Aminobenzoic acid (99%), N,N-dimethylacetamide (DMAc, >99.8%), 1-methylpyrrolidin-2-one (98%), phenylacetylene (97%), p-nitrochlorobenzene (99.5%), and p-nitroaniline (99%) were purchased from Shanghai Macklin Biochemical Co., Ltd. Dimethyl sulfoxide (DMSO), N,N-dimethylformamide (DMF), tetrahydrofuran (THF), toluene, ethanol (EtOH), isopropanol (iPrOH), acetonitrile (MeCN), ethyl acetate, 1,4-dioxane, toluene, acetone, dichloromethane (DCM), and nitrobenzene (99%) were purchased from Sinopharm Chemical Reagent Co., Ltd. N,N-Diethylformamide (DEF) and methanol (MeOH) were purchased from Shanghai Titan Co., Ltd. Palladium (II) nitrate dihydrate, cobalt(II) nitrate hexahydrate (99%), iron(III) sulfate (99%), 1-ethynyl-3-fluorobenzene (98%), p-nitrotoluene (99%), 4-ethylnitrobenzene (99%), and m-fluoronitrobenzene (97%) were purchased from Shanghai Aladdin Biochemical Technology Co., Ltd. Thiourea (98%) was obtained from Anhui Zesheng Technology Co., Ltd. Additionally, 4-ethylphenylacetylene (98%), 4-ethynylanisole (99%), 4-tert-butylphenylbutadiyne (98%), and 4-nitroacetophenone (98%) were purchased from Adamas Reagent Co., Ltd., while 3-ethynylanisole (96%) was obtained from Shanghai Meryer Chemical Technology Co., Ltd. were purchased from Shanghai Bide Pharmatech Co., Ltd.. All reagents were used as received without further purification.

Characterization

Solid-state nuclear magnetic resonance (SS-NMR): Solid-state NMR spectra were measured using an Avance Neo 400WB NMR spectrometer (Bruker, Sweden). For CP-MAS, an HFX DVT probe equipped with a 3.2 mm zirconia rotor was used at a spinning frequency of 10 kHz. The frequency scale in ppm was referenced to the adamantane C-H peak. ^{13}C CP-MAS NMR spectra were recorded with a contact time of 20 ms.

Scanning electron microscopy (SEM): SEM images were obtained using Sigma 360 (Zeiss, Germany) with a secondary electron detector, operating at 3 kV using a Schottky thermionic emission electron gun. Before imaging, the samples were sputtered with platinum (nano-sized film) for 60 s using a HITACHI MCIOOO ion sputter to avoid charging during SEM analyses. The samples were prepared simply by putting a drop (about 1μL) of dispersed samples in volatile solvents on clean aluminum foil.

Particle size analysis: The average sizes of HOMs were measured by Partica LA-960V2 (Horiba, Japan).

Ultrathin Sections: HOMs were immersed in K4M resin (London Inc., UK), which was polymerized under ultraviolet light. Ultra-thin sections (70 nm) of the sample blocks were cut by a Leica UC 6 microtome (Leica, Austria) with a diamond knife (Diatome, Switzerland), and placed onto 200 mesh copper grids (Zhong Jing Ke Yi, Beijing) for analysis with transmissive electron microscopy.

Transmission electron microscopy (TEM): TEM images were obtained using HT-7820 (HITACHI, Japan) at an accelerating voltage of 100 kV. High-resolution transmission electron microscopy (HR-TEM) and high-angle angular dark-field scanning transmission electron microscopy (HADDF-STEM) images were obtained using JEM-2100F (JEOL, Japan).

Spontaneous Raman spectra measurements: Spontaneous Raman spectra measurements were performed on a home-built microscope setup. The micro Raman system is based on an optical microscope (SopTop, China) used to focus the excitation light and collect it in a back-scattering configuration with a 532 nm longpass filter. An MLL-U-532 solid laser (Keri Photonics, U.S.A.) with an output wavelength of 532 nm was used as an excitation light source. The spontaneous Raman spectra were collected and analyzed by an Acton SpectraPro SP-2300 spectrometer (Princeton Instruments, U.S.A.) coupled with an electrically cooled electron-multiplying charge-coupled device (EMCCD) ProEM16002 detector (Princeton Instruments, U.S.A.). The final data have been averaged over ten accumulations to maximize the signal-to-noise ratio. The measurements were conducted at room temperature and in air. The laser power intensity has been kept at the order of 50 mW in order to avoid any sample degradation effects.

Fourier Transform Infrared Spectroscopy (FT-IR): FT-IR spectra were collected using Nicolet iS10 (Thermo Fisher Scientific, U.S.A). All samples were prepared using the potassium bromide pellet method.

Thermal Analysis (TGA): TGA was carried out on a TA SDT Q600 under nitrogen or air atmosphere from room temperature (around 28 °C) to 700 °C, along with a ramp rate of 10 °C min^{-1}. The samples were dried ahead by a vacuum oven at 1 Pa and 40 °C for 12 h. Thermogravimetric-mass spectrometry (TG-MS) data of the organic microspheres were collected using a STA 449 F3 (Netzsch, Germany) to record thermogravimetric changes, coupled with a QMS 403Q (Netzsch, Germany) to capture gas-phase mass spectrometry data at corresponding temperatures. Thermogravimetric- infrared spectroscopy (TG-IR) data were collected using a TGA 55 (Waters, U.S.A.), coupled with a Nicolet iS50 (Thermo Fisher Scientific, U.S.A.) to collect IR spectrometry. Pyrolysis gas chromatographs were obtained by GCMS-QP2020NX (Shimadzu, Japan), equipped with EGA3030D (Frontier Lab., Japan) as pyrolysis apparatus.

Powder X-ray diffraction (PXRD): PXRD patterns were recorded on a D/Max-2550pc (Rigaku, Japan), using Cu Kα radiation (tube operating at 40 kV and 250 mA) with a scintillation detector.

Inductively coupled plasma-Mass Spectrometry (ICP-MS): Pd-SMCs was weighted and then transferred into a beaker, followed by the addition of 25 mL of HNO_3 and 10 mL of $HClO_4$. The mixture was boiled at 180°C until no solid residues remained. Subsequently, 20 mL of HCl was added dropwise, and heating continued until the solution became clear. The final solution was diluted to 100 mL and filtered using a 0.22 μm membrane filter. The mass spectrometry was collected by ICP-MS 7700 (Agilent, U.S.A).

X-ray photoelectron spectroscopy (XPS): Approximately 15 mg of the powder sample was pressed into a pellet with a diameter of about 5 mm and mounted onto the sample holder. XPS data were collected on K-Alpha (Thermo Fisher Scientific, U.S.A.). The X-ray spot size was set to 400 μm, with an operating voltage of 12 kV and a filament current of 6 mA. The survey spectra were recorded with a pass energy of 150 eV and a step size of 1 eV, while high-resolution spectra were collected with a pass energy of 50 eV and a step size of 0.1 eV. All spectral acquisition parameters were set for automatic measurement by the instrument software. For the EDA treated samples, 15 mg of the material was dispersed in 15 mL of EDA by ultrasonication for 30 min to ensure a homogeneous suspension. The dispersion was then transferred to a sealed pressure tube and stirred at 40 °C for 24 h. After cooling to room temperature, the solid was washed three times with deionized water and dried overnight.

X-ray absorption fine structure spectroscopy (XAFS): The XAFS spectra at Pd K-edge were collected at BL14W beamline of Shanghai Synchrotron Radiation Facility (SSRF) using the Si(311) mode. The corresponding reference samples were collected in transmission mode

Temperature-programmed desorption (TPD): The TPD spectra were collected by BELCat II (Microtrac, Japan). A 40 mg sample was placed in a reaction tube and subjected to a drying pretreatment by heating at a rate of 10 °C/min from room temperature to 150 °C, under a He flow for 1 hour. After cooling to 25°C, a 10% C_2H_4/He or C_2H_2/He gas mixture was introduced for 1 hour to saturate the sample. The system was then switched to a He purge for 1 hour to remove weakly physiosorbed C_2H_4 or C_2H_2 from the surface. Finally, desorption was carried out under a He atmosphere, with a heating rate of 10 °C/min up to 500 °C, and the desorbed gases were monitored by mass spectrometry.

Gas chromatography (GC): GC analyses were performed on a GC-2030 (Shimadzu, Japan) instrument using CYCLOSIL-B 30 m × 0.25 mm × 0.25 μm chiral column (Agilent, U.S.A.). Gas Chromatography-Mass Spectrometry (GC-MS) analyses were performed on a GCMS-QP2010 SE (Shimadzu, Japan) instrument using a SH-1 30 m × 0.25 mm × 0.25 μm column (Shimadzu, Japan).

Solvent contact angle: Solvent contact angles were recorded using an OCA20 (Dataphysics, Germany). To ensure consistent surface roughness across measurements, a pressed pellet method was employed for sample preparation. Specifically, 10 mg of the powder sample was placed onto a glass slide and covered with a second glass slide. A pressure of 500 kPa was then applied to the slides and maintained for 30 seconds. After carefully removing the top glass slide, a 10 μL droplet of the test solvent was dispensed onto the flattened sample surface using a syringe. The contact angle was recorded once the droplet became stationary.

Variable-Temperature FTIR (VT-FTIR): VT-FTIR spectra were recorded on a VERTEX 80v spectrometer (Bruker, Germany) operating in diffuse reflectance infrared Fourier transform spectroscopy (DRIFTS) mode. The measurements were conducted under a continuous N_2 atmosphere over a temperature range of 150 to 450 °C. The system was heated at a rate of 10 °C/min, and the sample was held isothermally at each target temperature for 2 minutes prior to spectral data acquisition. All spectra were collected in the wavenumber range from 4000 to 400 cm^{-1}.

Differential Scanning Calorimetry (DSC): DSC analysis was performed using a Discovery DSC 25 thermal analyzer (TA Instruments, U.S.A.). The entire measurement was conducted under a continuous nitrogen purge at a flow rate of 50 mL/min. The sample was heated from

100 °C to 300 °C at a heating rate of 10 °C/min, and the corresponding heat flow curve was recorded.

Synthetic procedure of HOMs

2,4,6-Trihydroxybenzene-1,3,5-tricarbaldehyde (100 mg, 475.9 μmol) was dissolved in 25 mL DMAc, and 4-aminobenzoic acid (131 mg, 955.2 μmol) was dissolved in 25 mL iPrOH. After directly mixing the two solutions, a clear yellow solution was obtained. Precipitate occurred after several minutes, which was isolated by centrifugation (10,000 x g, 5 min) after 1 hr. The precipitate was washed with iPrOH three times to remove the excess amine and residue DMAc. The final products (4,4'-(5-(hydroxymethylene)-2,4,6-trioxocyclohexane-1,3-diylidene)-bis(methaneylylidene))-bis(azanediyl))dibenzoic acid) was dried in a vacuum oven at 40 °C and 1 Pa. The amount of product was measured to be 180 mg in 84% yield. The synthetic procedures of other HOMs were similar to HOM-1, replacing 2,4,6-trihydroxybenzene-1,3,5-tricarbaldehyde with altered dialdehyde reactants and 4-aminobenzoic acid with altered amine reactants. The structure of the amine and dialdehyde is listed in the table S1. Besides, the choice of aprotic solvent (solvent A) and protic solvent (solvent B) will also influence the final assembly structure. Solvents A and B used for the synthesis of HOMs are listed in the table S2. Pd content of diverse Pd-MCs is measured by EDS and is listed in table S3.

	Aldehyde	Amine
SMC-1	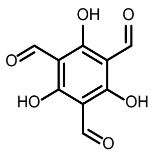	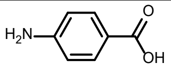
SMC-2		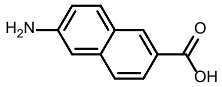
SMC-3		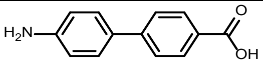
SMC-4	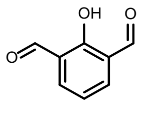	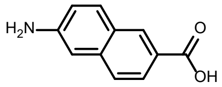
RMC-5		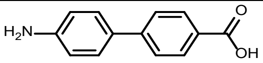
RMC-6	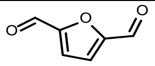	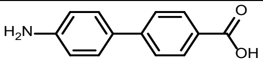

Table S1 Assembly molecules of MCs precursor materials

	Solvent A	Solvent B	Reaction time
SMC-1	DMAc	iPrOH	1 hr
SMC-2	DMAc	1,4-dioxane	1 hr
SMC-3	NMP	iPrOH	1 hr

SMC-4	Toluene	MeOH	12 hrs
RMC-5	DMAc	iPrOH	12 hrs
RMC-6	DMAc	MeOH	12 hrs

Table S2 Synthetic conditions for MCs

No.	Pd content (wt %)
**Pd-SMC-1**	39%
**Pd-SMC-2**	20%
**Pd-SMC-3**	31%
**Pd-SMC-4**	37%
**Pd-RMC-5**	46%
**Pd-RMC-6**	23%

Table S3 Pd content in diverse Pd-MCs

Synthesis of X-MC-1

10 mg of HOM was dispersed in 500 μL of deionized water and ultrasonicated at room temperature for 10 minutes to achieve a uniform suspension. Subsequently, 500 μL of metal salt solution (metal concentration: 0.045 mol L-1) was added to the suspension, and the mixture was vortexed for 12 hours. The solid was separated by centrifugation (10,000 x g, 5 min) and washed three times with deionized water, then dried overnight in a vacuum drying oven to obtain the intermediate product, denoted as X-HOM-1 (X = metal). The dried product was subsequently calcined under a nitrogen atmosphere, heating from room temperature to 450 °C at a rate of 5°C min-1 and holding at 450 °C for 2 hours, followed by cooling to room temperature under nitrogen flow, yielding the final product, X-SMC-1.

A similar procedure was used to synthesize X-SMC-1 (X = Fe, Co, Ni, Rh, Pd, Pt). For Fe-SMC-1, $Fe_2(SO_4)_3$ were used as Fe sources; for Co-SMC-1, $Co(OAc)_2$ was used as the Co source; for Ni-SMC-1, $Ni(NO_3)_2$ was used as the Ni source; for Rh-SMC-1, Rh(NO3)2 was used as the Rh source; for Pd-SMC-1, $Pd(NO_3)_2$ was used as the Pd source; and for Pt-SMC-1, K_2PtCl_4 was used as the Pt source.

Synthesis of Reference Catalysts

Post-Pd-SMC-1: 20 mg of calcined MC were dispersed in 2 mL of deionized water and ultrasonicated for 30 min to obtain a homogeneous suspension. Aqueous ammonia was then added to adjust the pH to 10, followed by the addition of 1 mL of an aqueous solution containing 5 mg of $Pd(NO_3)_2 \cdot 2H_2O$. The mixture was stirred at 80 °C in a sealed pressure tube for 3 h. After cooling to room temperature, an aqueous solution of sodium borohydride (10 equiv.) was

added, and the reaction was stirred at room temperature for another 3 h. The resulting solid was washed three times with deionized water and dried overnight.

$Pd_{0.1}$-SMC-1: 10 mg of HOM-1 was dispersed in 500 μL of deionized water and sonicated at room temperature for 10 min to obtain a homogeneous suspension. Subsequently, 500 μL of an aqueous palladium nitrate solution (metal concentration: 0.0045 mol L^{-1}) was added to the suspension, and the mixture was vortexed for 12 h. The solid was isolated by centrifugation (10,000 × g, 5 min), washed three times with deionized water, and dried overnight in a vacuum oven to yield the intermediate product, denoted as $Pd_{0.1}$-HOM-1. The dried product was then calcined under a nitrogen atmosphere by heating from room temperature to 450 °C at a rate of 5 °C/min. After being held at 450 °C for 2 h, the sample was cooled to room temperature under a continuous nitrogen flow to afford the final product, $Pd_{0.1}$-SMC-1.

Pd-BAC: 60 mg of 2,4,6-trihydroxybenzene-1,3,5-tricarbaldehyde were dissolved in 15 mL of N,N-dimethylformamide (DMF), and 79 mg of 4-aminobenzoic acid were suspended in 15 mL of water. After ultrasonication for 10 min to ensure homogeneous dispersion, the two solutions were directly mixed and allowed to stand in the dark for 1 h. The resulting precipitate was collected by centrifugation, washed three times with methanol, and dried overnight in a vacuum oven to afford fibrous assembly (FA). 10 mg of FA were dispersed in 500 μL of deionized water and ultrasonicated at room temperature for 10 min to obtain a uniform suspension. Subsequently, 500 μL of a $Pd(NO_3)_2 \cdot 2H_2O$ solution (metal concentration: 0.045 M) were added, and the mixture was vortexed for 12 h. The solid was separated by centrifugation (10,000 × g, 5 min), washed three times with deionized water, and dried overnight in a vacuum oven to yield the intermediate Pd-FA. The dried product was then calcined under a nitrogen atmosphere by heating to 450 °C at a rate of 5 °C min^{-1} and holding at this temperature for 2 h, followed by cooling to room temperature under flowing nitrogen to afford the final product, Pd-BAC.

Surface modelling of Pd-SMC-1

SEM images of HOMs or MCs were processed using ImageJ, where particles were approximated as ellipsoids. The major (A), intermediate (B), and minor (C) axes were defined, with a coordinate system established using the major axis as the x-axis, the minor axis as the y-axis, and the intermediate axis as the z-axis. In ImageJ, the origin was set at the top-left corner, and a scale bar was used to parameterize the image length. Boundaries of the particles were marked by selecting the topmost/bottommost points (y values) and the side points (x values).

The coordinates of these points were exported. The center of the ellipsoid was determined by the intersection of lines connecting the maximum/minimum y-axis and x-axis points, with the coordinates represented as (a, b, 0). The differences in the x- and y-axis values were recorded as 2A and 2B, respectively, assuming C = B. Based on these parameters, the surface equation of HOMs and SMCs was constructed.

$$\frac{(x-a)^2}{A}+\frac{(y-b)^2}{B}+\frac{z^2}{C}=1 \quad \text{(S1)}$$

Here, (x, y, z) was the coordinate of a point. $(a, b, 0)$ were the centre coordinates of the ellipsoid. A, B, and C were the major axis, intermediate axis, and minor axis of the ellipsoid, respectively. The particle surface tip vertices were marked in ImageJ, and the coordinates of all points were obtained using the previously defined coordinate system. The data were then processed in MATLAB R2021a. Using the HOM surface model, the z-axis coordinates for all points were calculated.

In the closest packing mode, when tips are distributed on the sphere's surface, the maximum particle radius that can diffuse and contact the HOM's surface is 2/√3 times the tip-to-tip distance. However, the actual surface stacking of tips in HOMs deviates from the ideal model, making the theoretical value impractical. Therefore, we focus on point spacing in practice. Delaunay triangulation, a common method in computational geometry, was used to organize the points into a non-overlapping triangle network, ensuring that no other points are inside the circumcircle of each triangle.

Using the x-y projection of the point set, we applied the built-in Delaunay module in MATLAB R2021a for triangulation and point connectivity. Considering the z-axis coordinates, we calculated the average edge length. To account for boundary errors, where non-neighboring points may be incorrectly connected, we filtered out such erroneous edges. The first average edge length was calculated, and the threshold value, √3 times this average, was used to filter out longer edges. In the closest packing model, the distance to the second nearest tip is √3 times

the shortest distance. The second average length of the remaining edges was then calculated and used as the final result. The code for surface modelling is provided in Supplementary Code.

X-ray absorption fine structure (XAFS) analysis

The average coordination number of palladium (Pd) nanoparticles was determined through X-ray Absorption Fine Structure (XAFS) spectroscopy using the following procedure: XAFS data, including XANES and EXAFS regions, were collected at a synchrotron radiation source, with a Pd foil serving as a reference sample for energy calibration. The EXAFS oscillation signals were processed using software such as Demeter[1]. This preprocessing includes background subtraction, k-space weighting (usually k^2 or k^3), and Fourier transformation to obtain the radial distribution function (RDF). A theoretical scattering path model was built based on the face-centered cubic (FCC) crystal structure of Pd (AMCSD code: 11155), focusing on the first coordination shell (Pd-Pd bonds). The EXAFS signal, χ(k), which is the sum of all contributions, χi(k), from groups of atoms that lie at approximately equal distances from the absorbing atom, was adjusted by applying the EXAFS equation, written in the following extended form:

$$\chi(k) = \sum_j \frac{S_0^2 N_j f_j(k)}{kR_j^2} e^{-2R_j/\lambda(k)} e^{-2k^2\sigma_j^2} \sin\left[2kR_j + \delta_j(k)\right] \tag{S2}$$

Where $\chi(k)$ is the EXAFS oscillation signal, k is photoelectron wave vector (related to the X-ray photon energy and the absorption edge threshold energy), N_j is the coordination number (CN) of the j-th shell refers to the number of neighboring atoms in the j-th shell surrounding the target atom, S_0^2 is the amplitude decay factor describes the attenuation of the EXAFS signal due to many-body effects, $f_j(k)$ is the backscattering amplitude of the scattering atoms in the j-th coordination shell, is the average bond length between the target atom and the scattering atoms in the j-th coordination shell, σ_j^2 is Debye-Waller factor (characterizes the disorder in the j-th coordination shell), $\lambda(k)$ is the mean free path of photoelectrons, $\delta_j(k)$ is phase shift correction term.

Using a least-squares fitting method, experimental data were fitted by optimizing parameters[2]. The S_0^2 was constrained based on bulk Pd EXAFS data (CN = 12). The reliability of the model was evaluated by the R-factor and χ^2 values. Then set the S_0^2 and fitting the N_j to obtain CN from the first coordination shell within Pd-SMC-1. The final CN was calculated to be 5.7.

The particle size was estimated using a correlation model between surface atom fraction and particle size. Considering the intrinsic coordination number (CN = 12) of the face-centered cubic (FCC) bulk structure, the empirical formula[3]:

$$CN = \frac{aN_{at}}{b+N_{at}} + \frac{cN_{at}}{d+N_{at}} \tag{S3}$$

where N_{at} is the number of atoms present in the cluster, a-d are four constants (a=8.981, b=9.640, c=3.026, d=1462.61). Substituting CN = 5.7 into the equation yields N_{at} = 17. This corresponds to a nanoparticle with a diameter of ~0.78 nm using the following equation[4]:

$$d = d_{Pd} \times \sqrt[3]{N_{at}/0.74} \tag{S4}$$

Where d is the diameter of the Pd particles, d_{Pd} is the bond length of Pd-Pd bond, 0.78 is the space filling factor of close packing. Also, when the d is set to 4 nm, the N_{at} is calculated to be 2045.9 with *CN* equaling to 10.7.

Catalytic performance evaluation for selective hydrogenation of nitrobenzen

For the reduction of nitrobenzene to azoxybenzene, nitrobenzene (15 μL) and the Pd catalyst (Pd: 0.24 mol%) were added to a 2 mL glass vial, followed by the addition of ethylenediamine as the solvent to adjust the total reaction volume to 400 μL. The vial was placed in a 14-position stainless-steel high-pressure reactor and purged with H_2 three times. The reaction was then carried out at room temperature under 2 bar of H_2 with stirring for 2 h. After completion, the mixture was centrifuged and analyzed by gas chromatography.

For catalyst poisoning experiment, 2.4 mol% thiourea or thiophene was added to each vial, and all other experimental conditions remained the same as previously described.

For the reduction of nitrobenzene to aniline, the solvent was changed from EDA to water, and the reaction time was prolonged to 6 hours. All other experimental conditions remained the same as previously described.

For solvent-free gram-scale hydrogenation, the reaction was conducted in a 25 mL stainless steel autoclave equipped with a glass vial containing nitrobenzene (1.5 mL, 18.4 mmol) and Pd-SMC-1 catalyst (0.5 mol% Pd relative to substrate). The system was purged three times with H_2, then pressurized to 10 bar H_2 and stirred at 80°C for 36 h. After cooling to room temperature, the catalyst was separated by centrifugation (12,000 x g, 10 min), and the crude mixture was purified via silica gel column chromatography (eluent: hexane/ethyl acetate = 5:1, v/v) to afford aniline as a yellow oil (1.2 g, 88% isolated yield).

The catalytic activity metrics were calculated as follows:

Turnover Number (TON):

$$TON = \frac{n_{product}}{n_M} \tag{S5}$$

Turnover Frequency (TOF, h^{-1}):

$$TOF = \frac{n_{product}}{n_M \times t} \tag{S6}$$

where $n_{product}$ is the molar amount of converted product (determined by GC analysis), n_M is the molar amount of metal addition, and t is the reaction time in hours.

Catalyst recycling test

A mixture of Pd-SMC-1 (0.15 mg), nitrobenzene (30 μL), and ethylenediamine (500 μL) was ultrasonicated to achieve a homogeneous dispersion and then transferred into a 2 mL glass vial. The vial was placed in a 14-position stainless-steel high-pressure reactor and purged with H_2 three times. The reaction was subsequently conducted at room temperature under 2 bar of H_2 for 2 h and repeated for five consecutive cycles. After each run, the catalyst was carefully recovered by centrifugation to avoid mass loss, washed twice with EDA, and reused under identical reaction conditions. Upon completion of each reaction, the mixture was centrifuged and analyzed by gas chromatography.

Catalytic performance evaluation for selective hydrogenation of phenylacetylene

The process for screening catalyst conditions for the hydrogenation of phenylacetylene is as follows. H_2 is used as the reducing agent, and the reaction is conducted in a stainless-steel high-pressure reactor. In a typical reaction, the Pd catalyst (Pd: 0.24 mol%) is added to a 2 mL glass vial and ultrasonically dispersed in 0.1 mL of the reaction solvent. Subsequently, 16 μL of phenylacetylene is added, and the solvent is supplemented to bring the reaction mixture to a total volume of 400 μL. The system is purged with H_2 three times. The reaction is carried out

at 2 bar and room temperature with stirring for 2 hours. After the reaction is completed, the mixture is centrifuged and analyzed by gas chromatography. For the other Pd catalysts, 0.24 mol% of the Pd catalyst is added. All other conditions remain the same as previously described, except that the reaction time is shortened to 1 hour.

The time-resolved monitoring of reaction conversion and selectivity was conducted by periodically sampling the reaction mixture at specified intervals. A mixture of 5 mg of Pd-SMC-1, 800 μL of phenylacetylene, and 20 mL of isopropanol is ultrasonically dispersed until uniform and transferred into a 25 mL stainless-steel high-pressure reactor. The reaction is then carried out at 20°C. To obtain the kinetic curve of the catalytic reaction, 500 μL of the reaction mixture is sampled every 10 minutes during the reaction.

Supplementary Figure

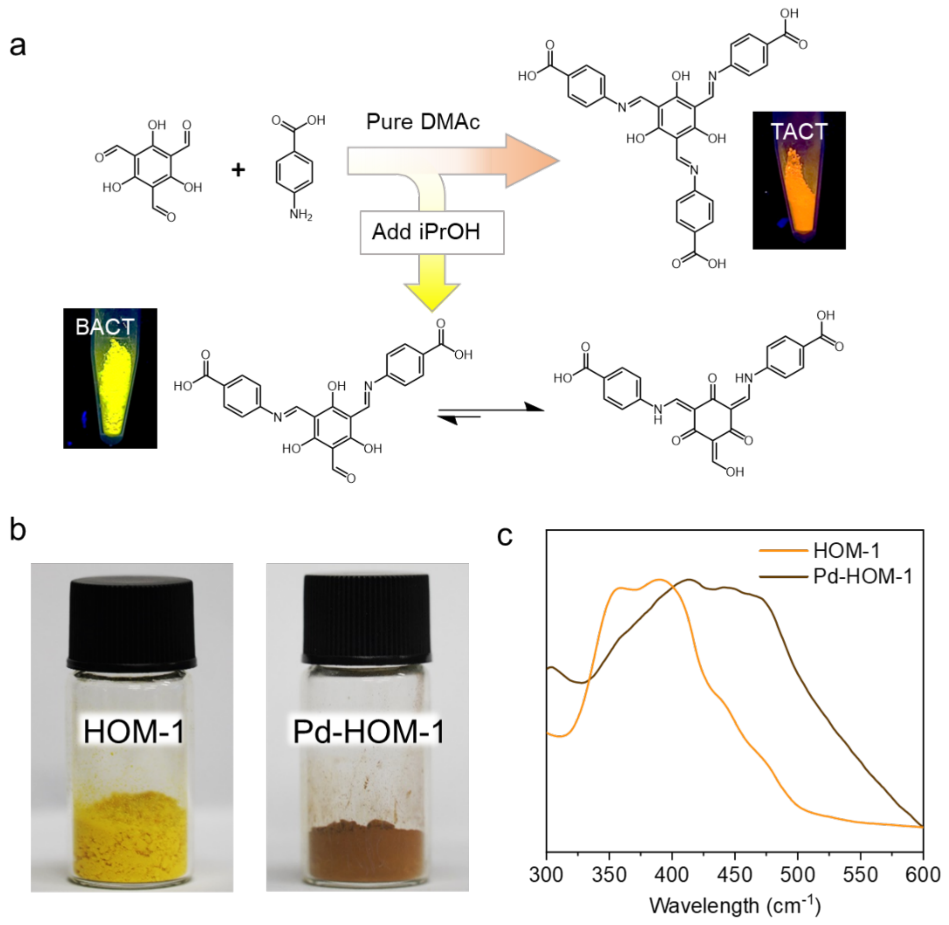

Figure S1. (a) Schematic illustration of the condensation conditions for the formation of TACT via triple condensation of 2,4,6-trihydroxybenzene-1,3,5-tricarbaldehyde with 4-aminobenzoic acid and for the formation of BACT (i.e., the assembling monomer of HOM-1) via double condensation. (b) Photos of the HOM-1 and Pd-HOM-1 powder. (c) UV-Vis spectra of HOM-1 before and after absorption of Pd.

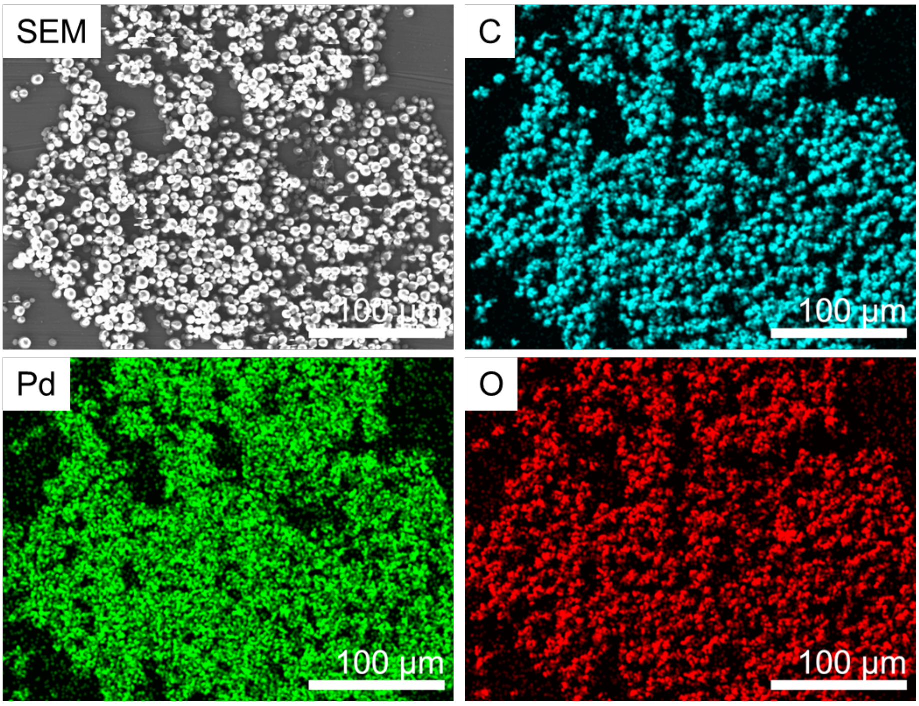

Figure S2. SEM image and EDS mapping images of Pd-HOM-1.

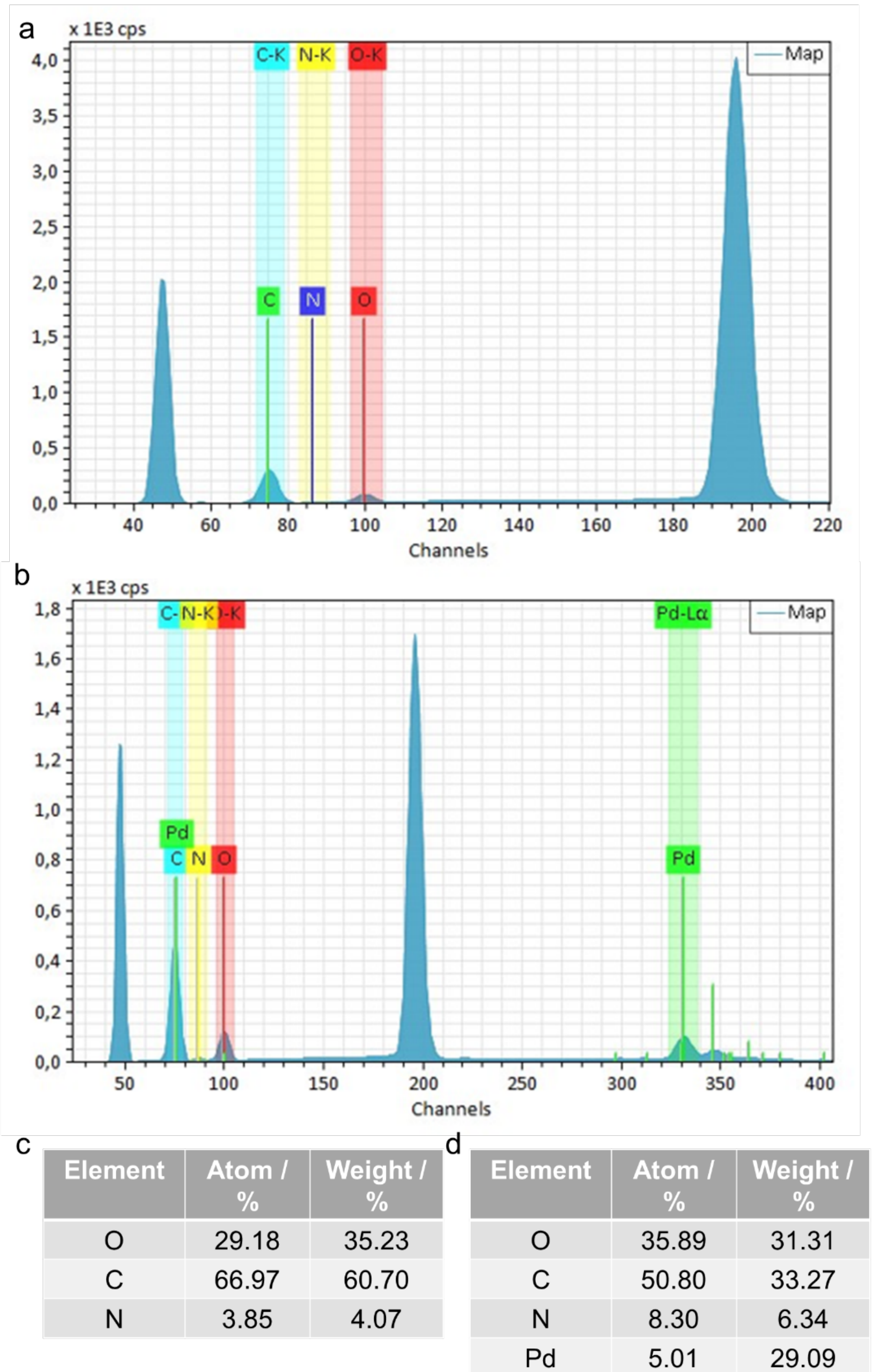

c

Element	Atom / %	Weight / %
O	29.18	35.23
C	66.97	60.70
N	3.85	4.07

d

Element	Atom / %	Weight / %
O	35.89	31.31
C	50.80	33.27
N	8.30	6.34
Pd	5.01	29.09

Figure S3. EDS spectrum of HOM-1 (a) and Pd-HOM-1 (b) The peak around 200 (channels) originated from aluminum since all samples were dispersed on aluminum foil. Distribution of elements based on EDS calculation for HOM-1 (c) and Pd-HOM-1 (d).

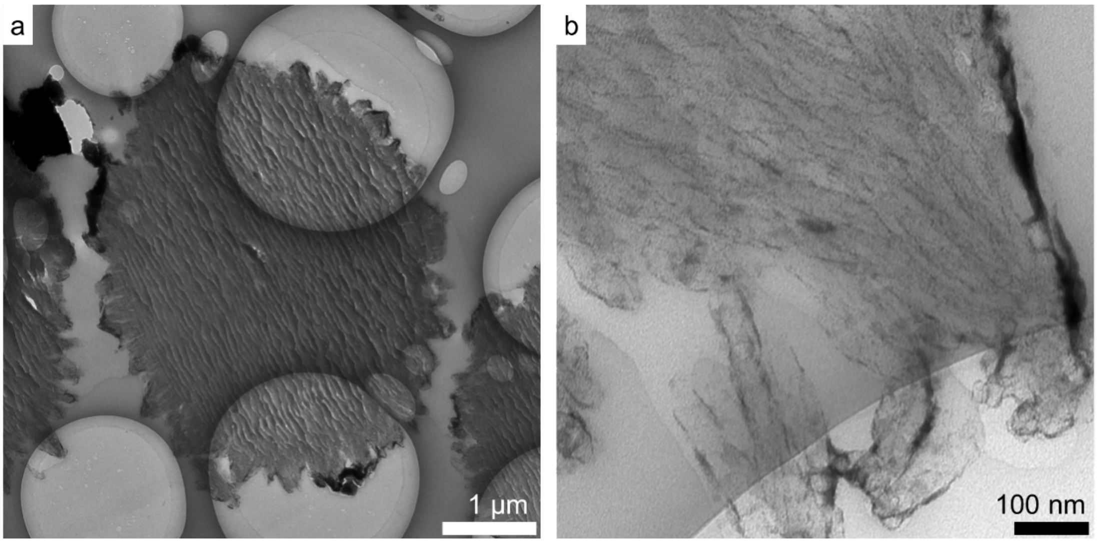

Figure S4. (a) The TEM images of the ultrathin section of Pd-HOM-1. (b)The sample was embedded in resin and ultrathin-sectioned to a thickness of 100 nm.

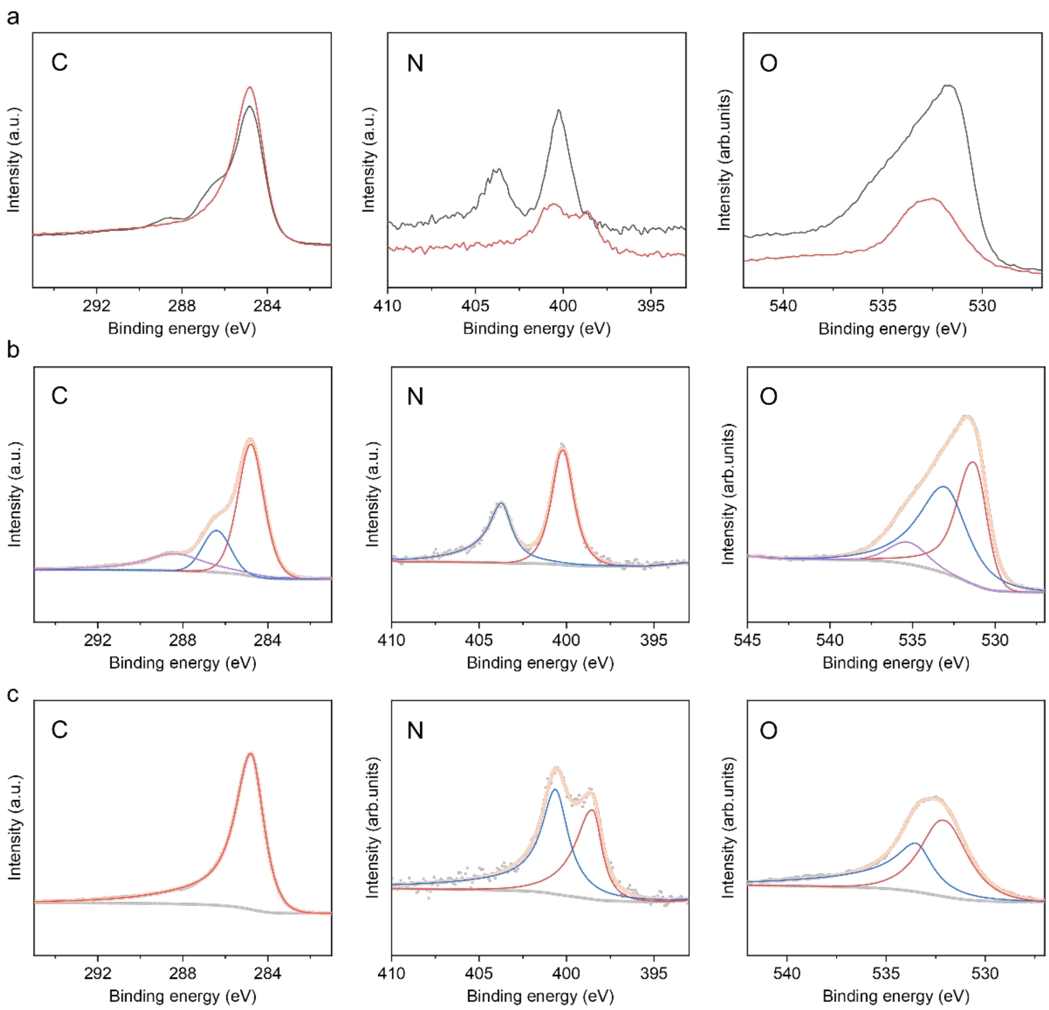

Figure S5. (a) XPS spectra of C 1s, N 1s and O 1s specimens for Pd-HOM-1 (dark line) and Pd-SMC-1 (red line). (b-c)**,** XPS peak fitting results of C 1s, N 1s and O 1s specimens for Pd-HOM-1 (b) and Pd-SMC-1 (c). For C, signals corresponding to C=O (288.5 eV), C–N (286.5 eV), and self-bonding (284.8 eV) were observed before calcination[5]. Post-calcination, the C=O and C–N peaks nearly vanished, leaving only the self-bonding peak. N displayed distinct nitrate signals (403.7 eV) before calcination, originating from the added $Pd(NO_3)_2$. After calcination, the N signal became significantly weaker. And the peak at 398.7 eV is primarily attributed to pyridinic nitrogen located at the edges of the carbon matrix, whereas the peak at 400.6 eV is ascribed to graphitic nitrogen embedded within the carbon framework. These observations suggest that extensive nitrogen loss occurred during pyrolysis, with the residual nitrogen atoms being structurally integrated into the graphitized carbon skeleton[6]. The O 1s peaks at 531.4 and 533.3 eV are respectively assigned to C=O and C-O for the carboxyl group. The peak at 535.3 eV arises from nitrate. After calcination, the peak intensity significantly decreased due to decarboxylation and the thermal decomposition of nitrate species, which were likely released as CO_2 and NO_x.

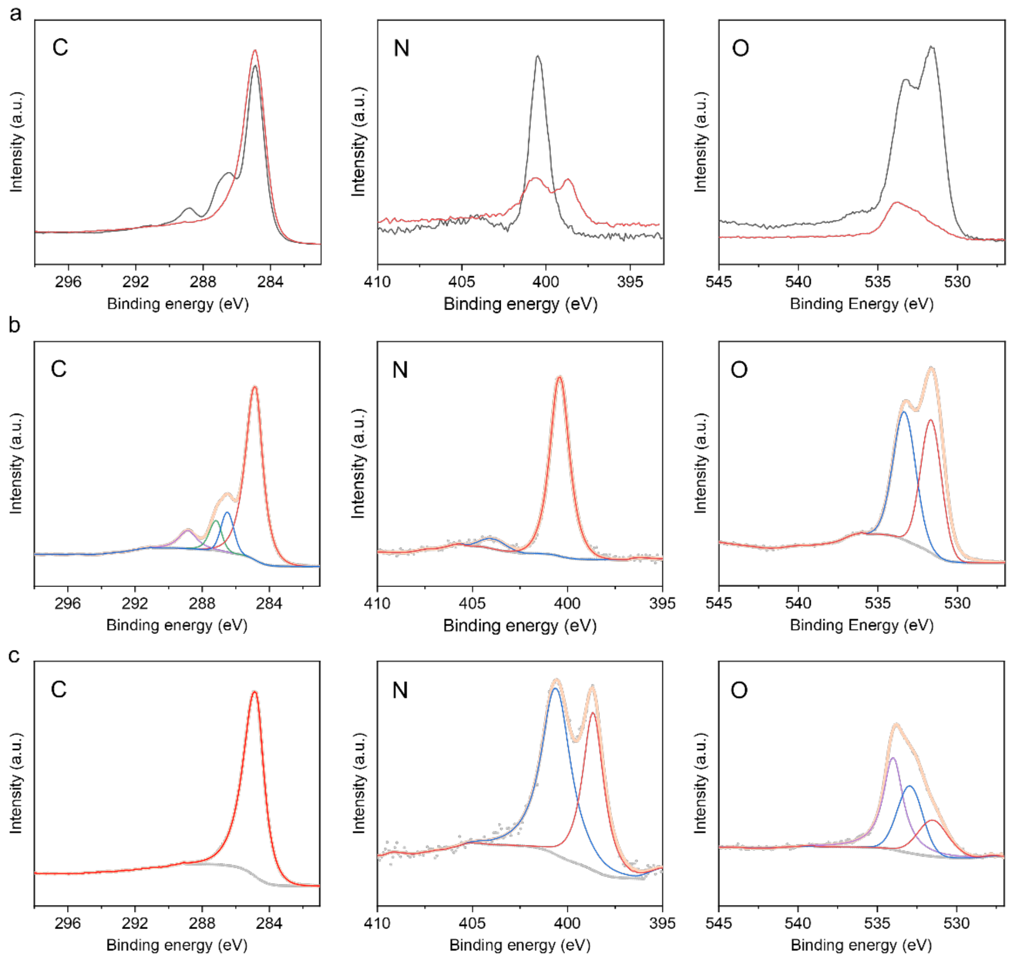

Figure S6. (a) XPS spectra of C 1s, N 1s, and O 1s specimens for HOM-1 (dark line) and SMC-1 (red line). (b-c)**,** XPS peak fitting results of C 1s, N 1s and O 1s specimens for HOM-1 (b) and SMC-1 (c). The intensity is normalized. For C, signals corresponding to C=O (288.8 eV for carboxyl group and 287.2 eV for ketone), C–N (286.5 eV), and self-bonding (284.8 eV) were observed before calcination[5]. Compared with the C signals of HOM-1, the downshift in the C=O binding energy after Pd loading is primarily attributed to electronic interactions between Pd species and carbonyl groups, which increase local electron density by back bonding. Post-calcination, the C=O and C–N peaks also nearly vanished, leaving only the self-bonding peak. In the N 1s region, a signal at 400.4 eV corresponding to C–N bond was observed before calcination, along with a weak peak near 404 eV that may originate from oxidized amine species[6]. Post-calcination, the remaining nitrogen species resembled those found in Pd-SMC-1, suggesting that residual nitrogen was incorporated into the carbon framework during pyrolysis. For O, the most prominent feature was the decrease of the carbonyl oxygen signal at 531.7 eV, confirming the decarboxylation during calcination.

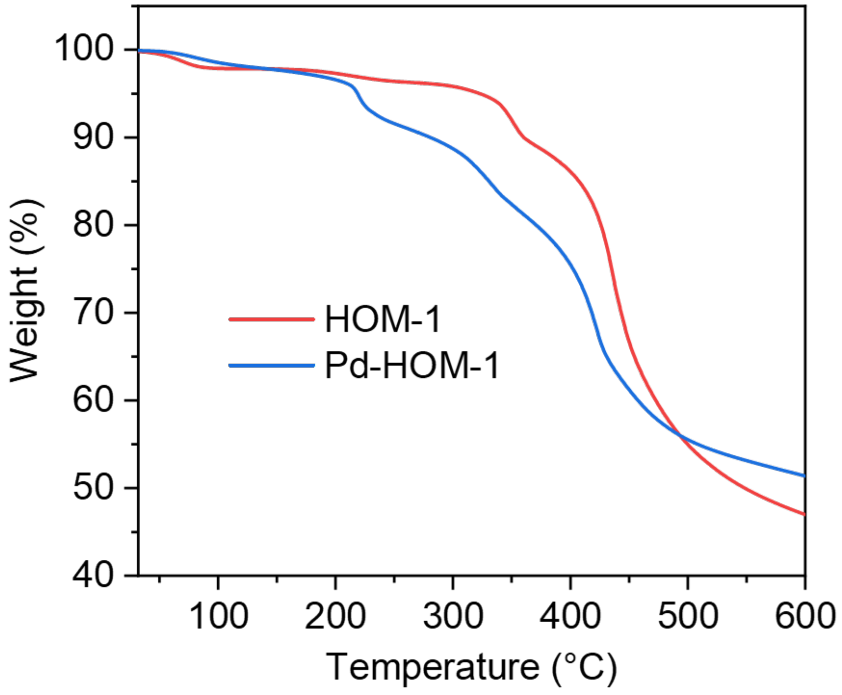

Figure S7. Thermogravimetric curves of Pd-HOM-1 and HOM-1 under nitrogen atmosphere. Before testing, the sample was dried in a vacuum oven for 12 hours. The heating rate during the test was 10.00 °C/min.

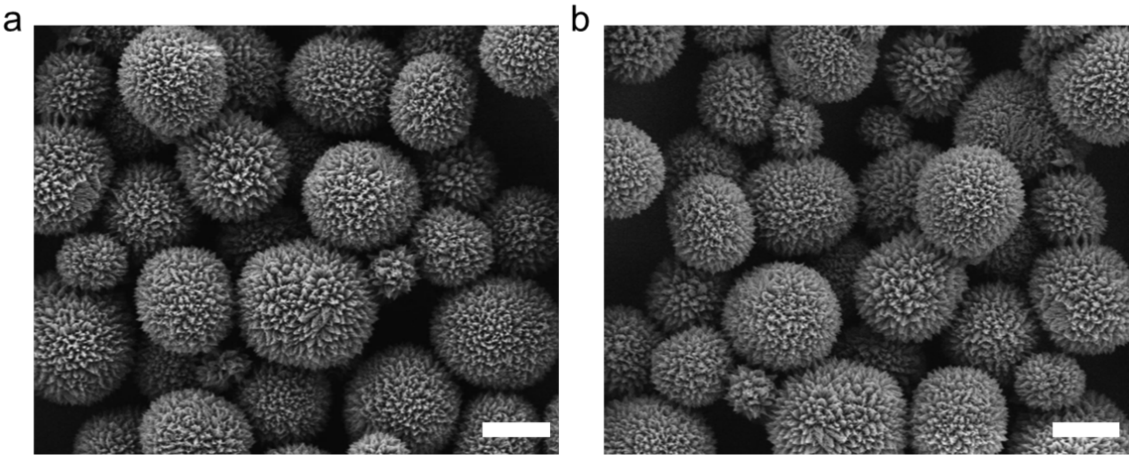

Figure S8. SEM images of HOM-1 (a) and Pd-HOM-1 (b). The scale bars are 3 μm.

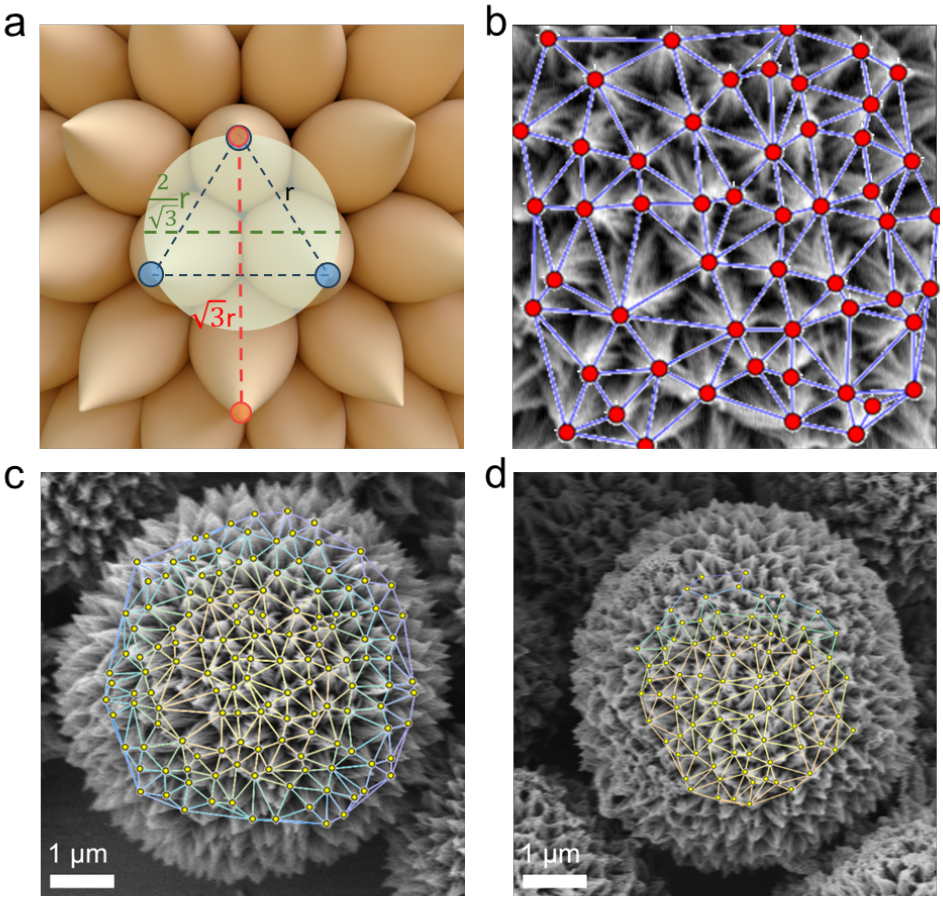

Figure S9. (a-b) The schematic graph for the Delaunay triangle partition. (c) Results of Delaunay triangle partition for HOM-1 particle, with calculated point-spacing of 0.48 ± 0.14 μm. (d) Results of Delaunay triangle partition for Pd-SMC-1 particle, with calculated point-spacing of 0.43 ± 0.12 μm.

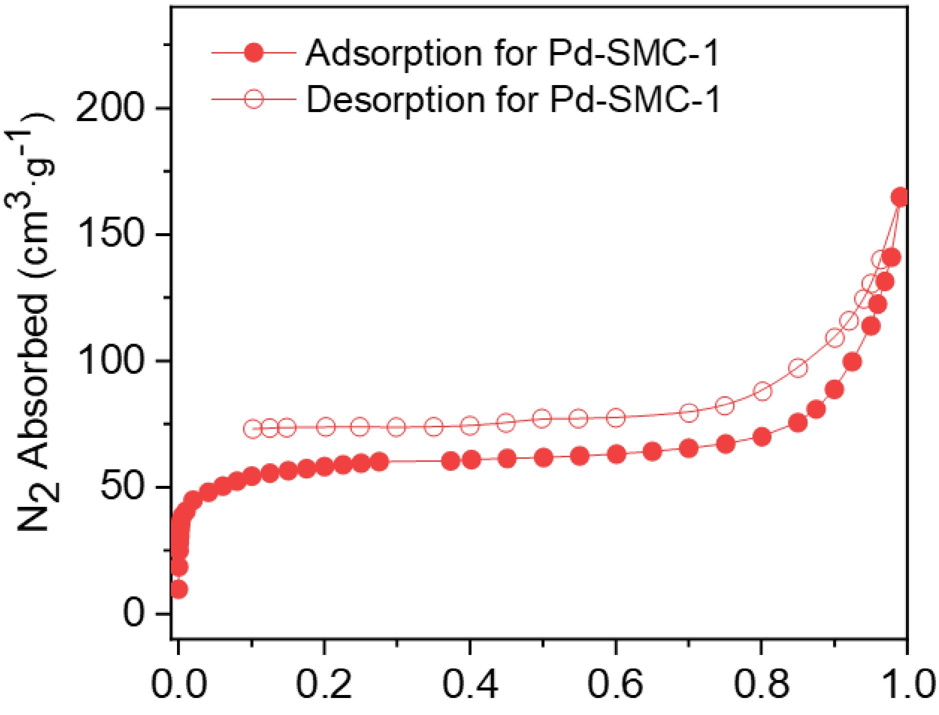

Figure S10. N_2 adsorption–desorption isotherms at 77 K of Pd-SMC-1.

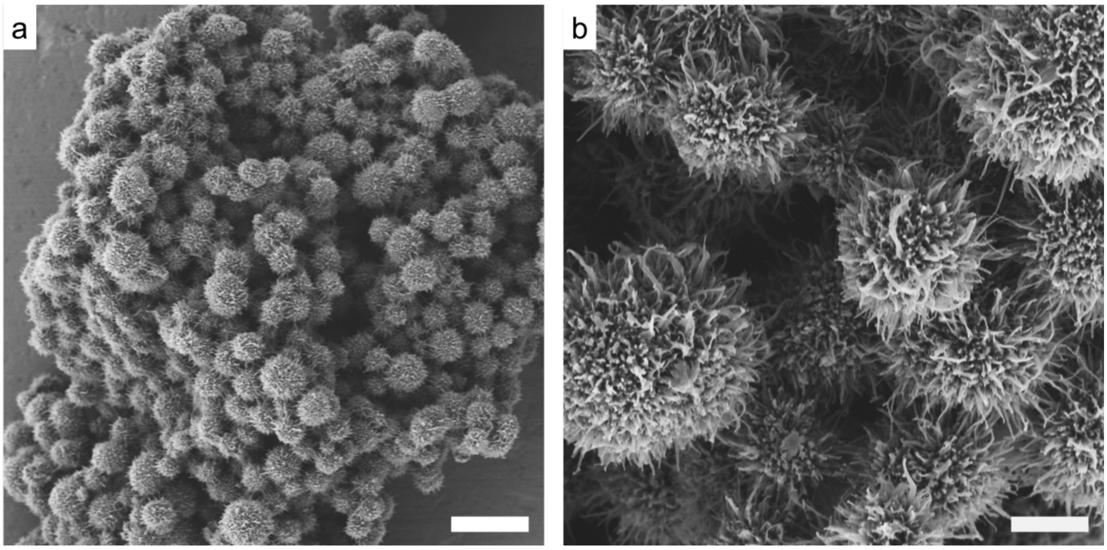

Figure S11. The SEM images of SMC-1 synthesized by directly calcining HOM-1 at 450 °C without prior immersion in the $Pd(NO_3)_2$ solution. (the left scale bar is 10 μm, and the right scale bar is 2 μm).

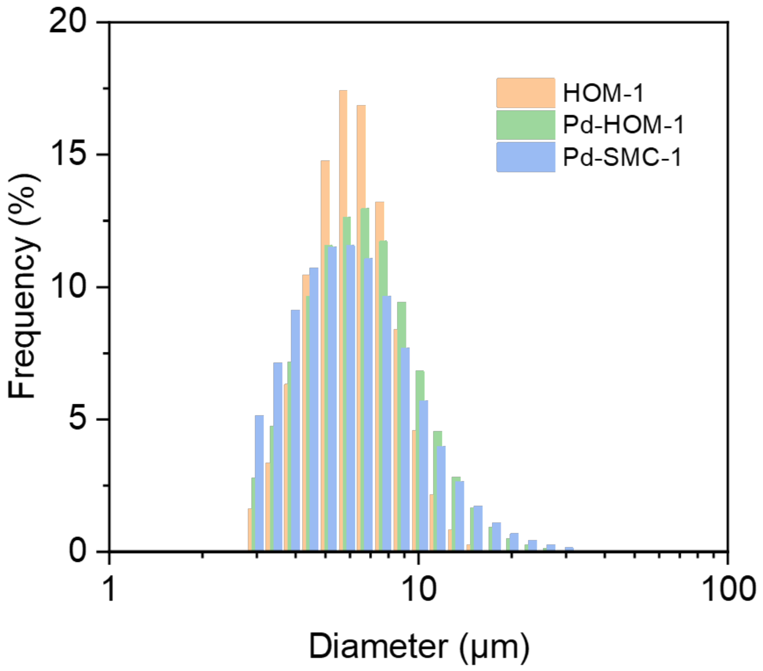

Figure S12. The size distribution of Pd-SMC-1 measured by static laser light scattering, compared with precursor materials.

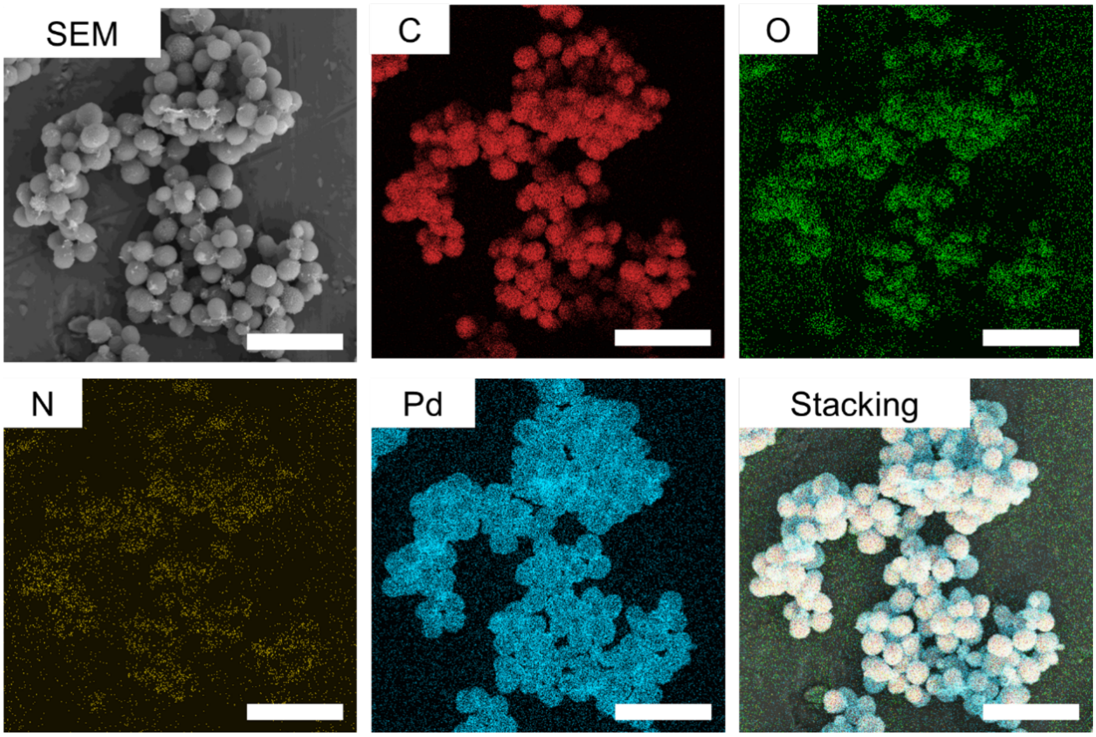

Figure S13. SEM image and elemental mapping images of Pd-SMC-1 (the scale bars are 20 μm). EDS mapping shows a strong spatial correlation between C and Pd. Compared with Pd-HOM-1, weaker signals of N and O suggest that most nitrogen- and oxygen-containing species were removed during calcination.

a

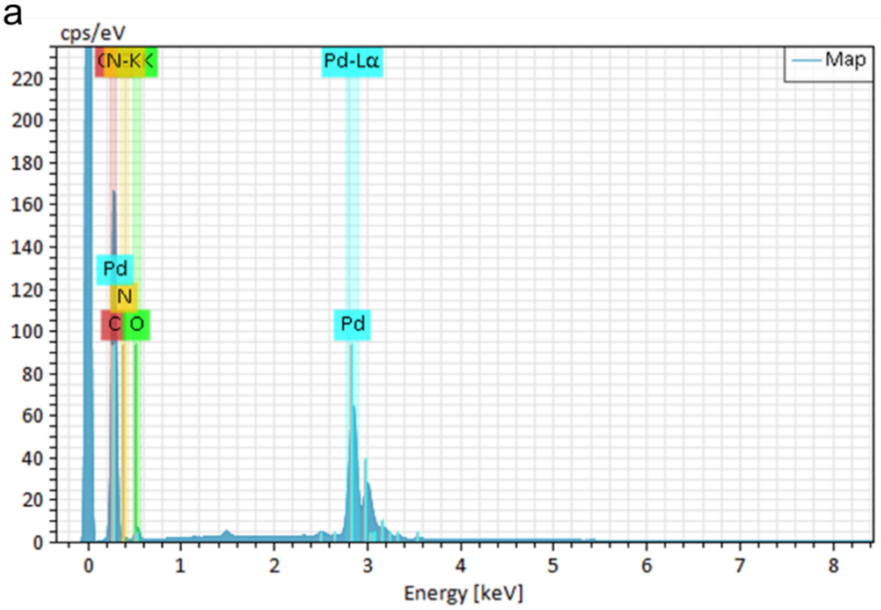

b

Element	Atom / %	Weight / %
O	18.22	14.74
C	68.41	41.56
N	6.06	4.29
Pd	7.32	39.40

Figure S14. (a) EDS spectrum of Pd-SMC-1. The peak around 1.5 keV originated from aluminum since all samples were dispersed on aluminum foil. (b)**,** Distribution of elements based on EDS calculation for Pd-SMC-1.

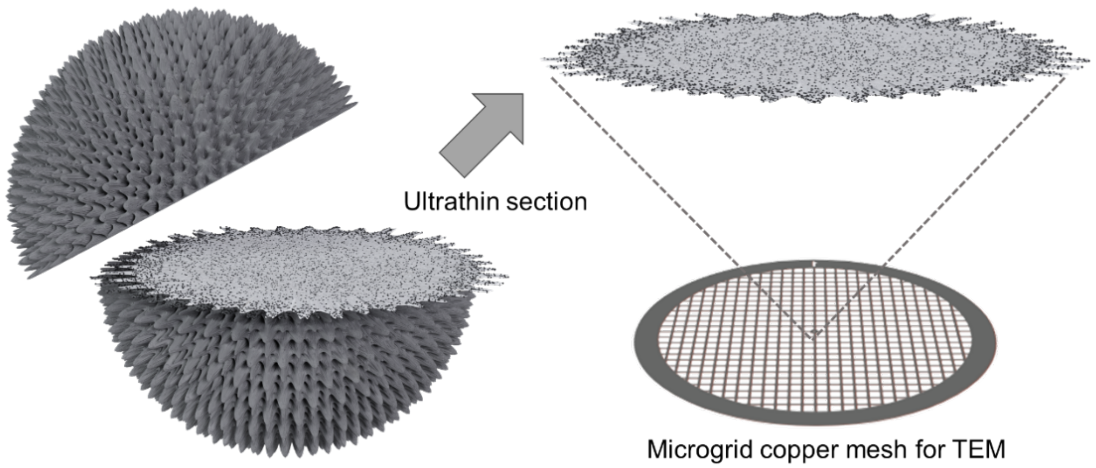

Figure S15. Schematic diagram of ultrathin-section TEM analysis. The detailed information about ultrathin-section can be found in the general information. In brief, samples were immersed in resin, followed by polymerization under ultraviolet light. All samples are cut into about slices with a thickness about 100 nm.

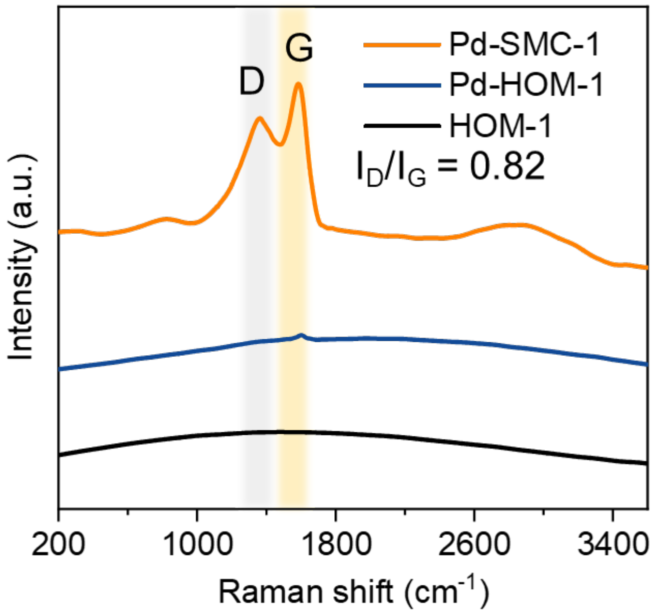

Figure S16. Raman spectra of Pd-SMC-1 compared with its precursor materials.

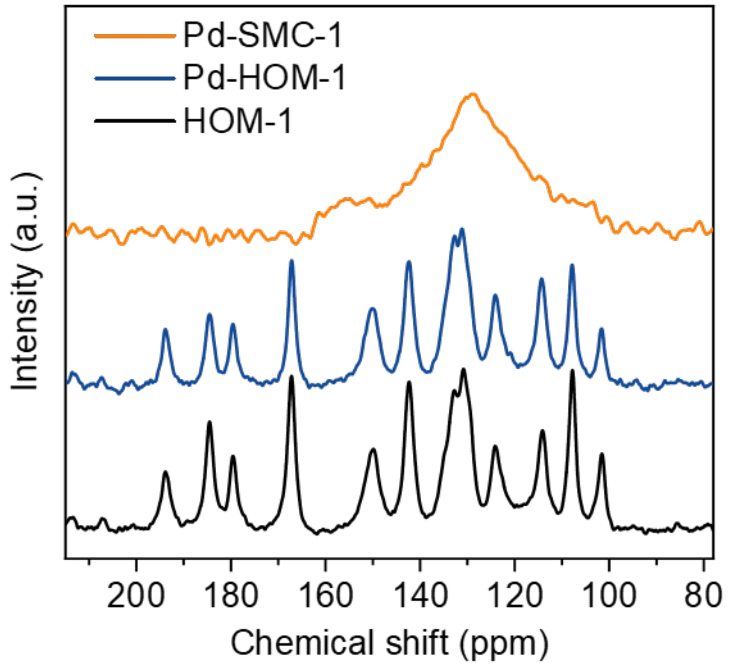

Figure S17. Solid-state ^{13}C NMR spectra of Pd-SMC-1 compared with its precursor materials.

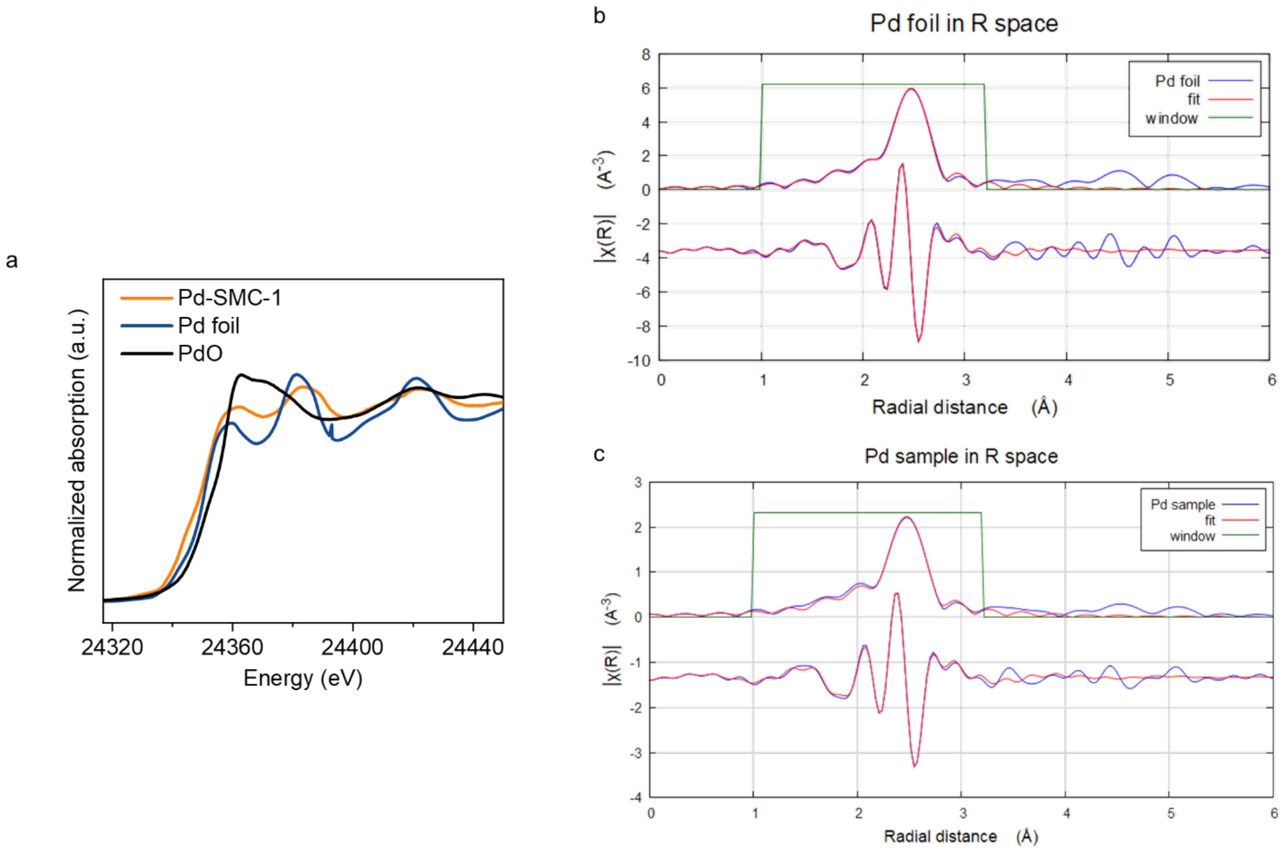

Figure S18. (a) Pd K-edge XANES spectra of Pd-SMC-1, compared with Pd foil and PdO. (b) Stacked plot of the magnitude (top) and imaginary (bottom) non-phase corrected Fourier-transformed Pd K-edge EXAFS of Pd foil. (c) Stacked plot of the magnitude (top) and imaginary (bottom) non-phase corrected Fourier-transformed Pd K-edge EXAFS of Pd-SMC-1.

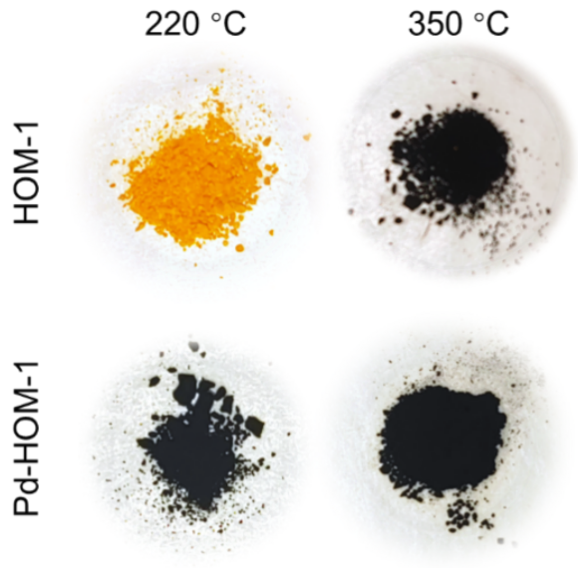

Figure S19. Photographs of HOM-1 and Pd-HOM-1 powder after heating to 220 °C and 350 °C.

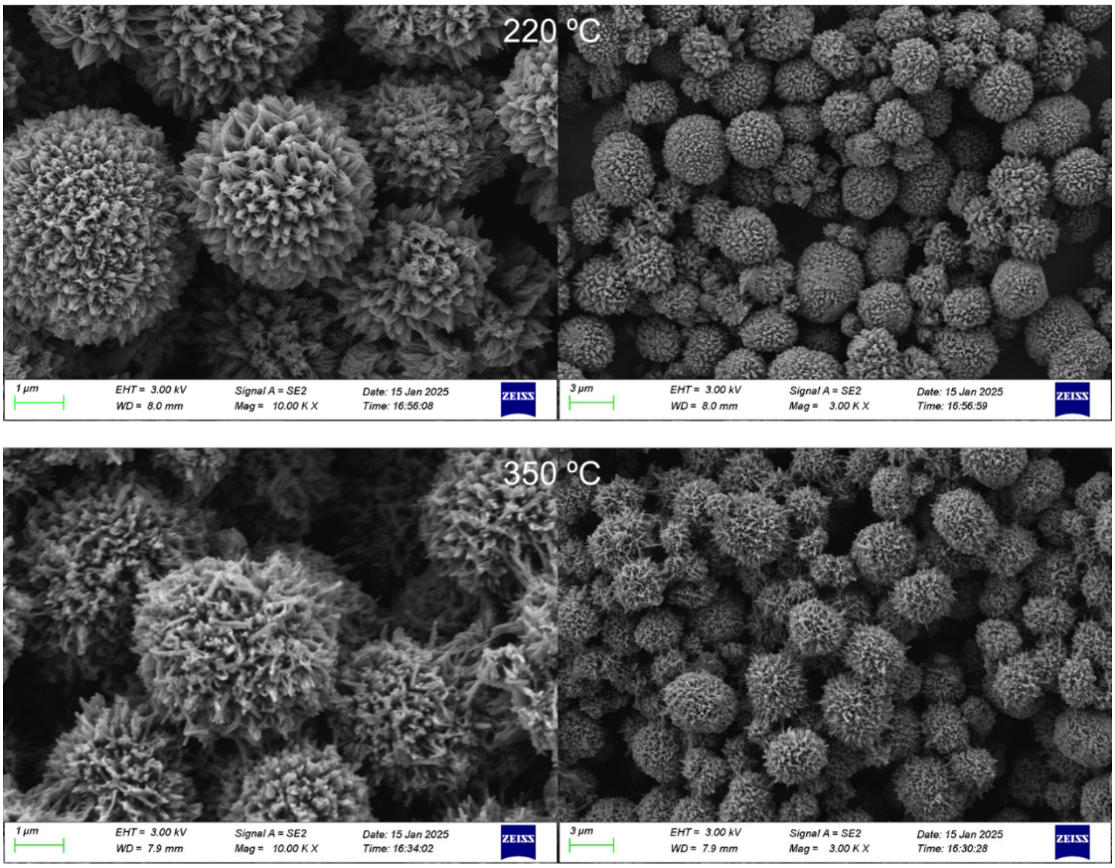

Figure S20. The SEM images of HOM-1 after heating to 220°C and 350°C, respectively. Pristine HOM-1 was calcined under a nitrogen atmosphere by heating from room temperature to either 220 °C or 350 °C at a rate of 5 °C/min, followed by immediate cooling to room temperature under continuous nitrogen flow.

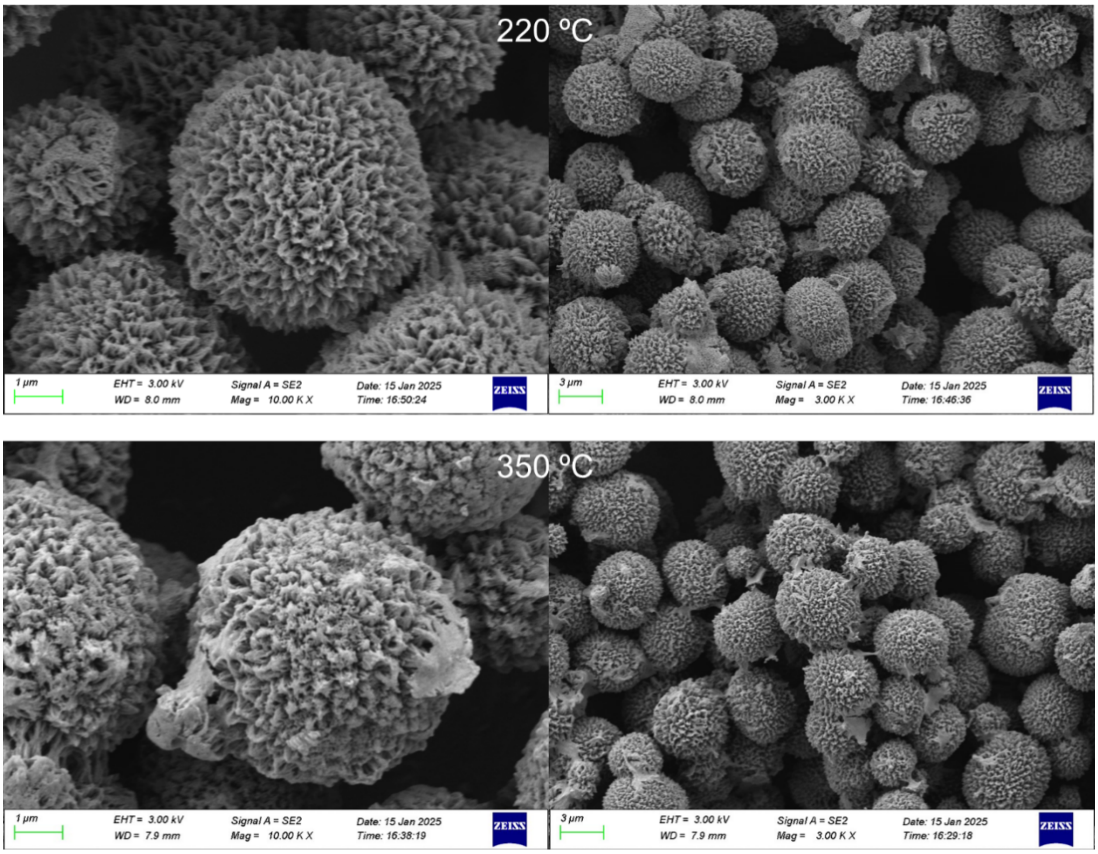

Figure S21. The SEM images of Pd-HOM-1 after heating to 220°C and 350°C, respectively. Pd-HOM-1 was calcined under a nitrogen atmosphere by heating from room temperature to either 220 °C or 350 °C at a rate of 5 °C/min, followed by immediate cooling to room temperature under continuous nitrogen flow.

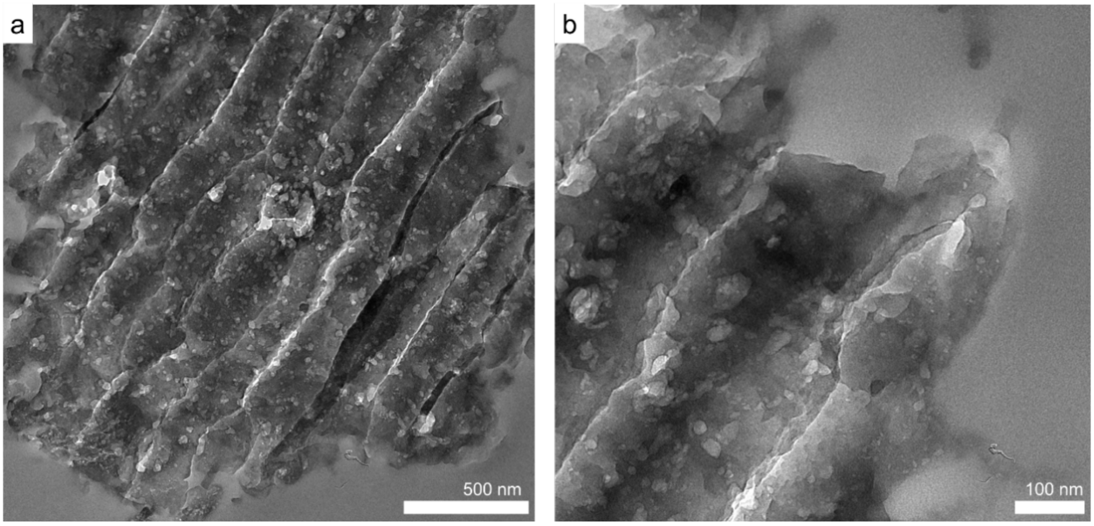

Figure S22. (a-b) The TEM images of the ultrathin section of SMC-1. The thickness of the slice is 100 nm. SMC-1 was obtained by directly calcining HOM-1 without metallization. In contrast to Pd-SMC-1, SMC-1 contains no internal nanoparticles due to the absence of metal species. Instead, numerous hollow cavities are observed, which appear as lighter, irregular voids in the slices. This indicates that, in the absence of Pd to stabilize the framework, significant structural collapse occurs within the material during calcination.

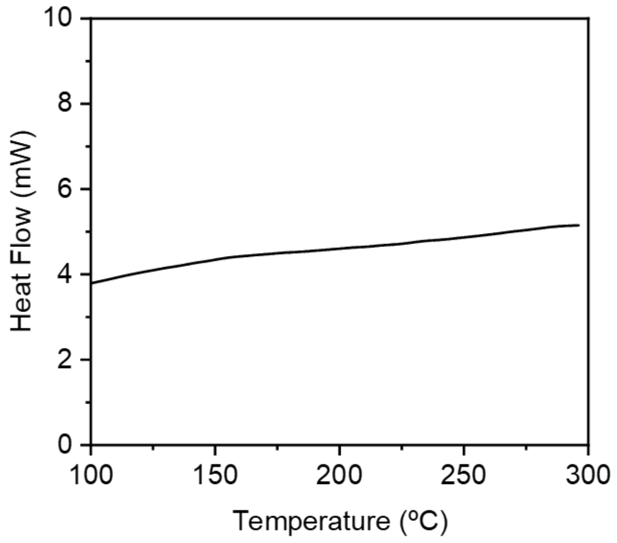

Figure S23. DSC curve of HOM-1 under an N_2 atmosphere (heating rate: 10 °C/min).

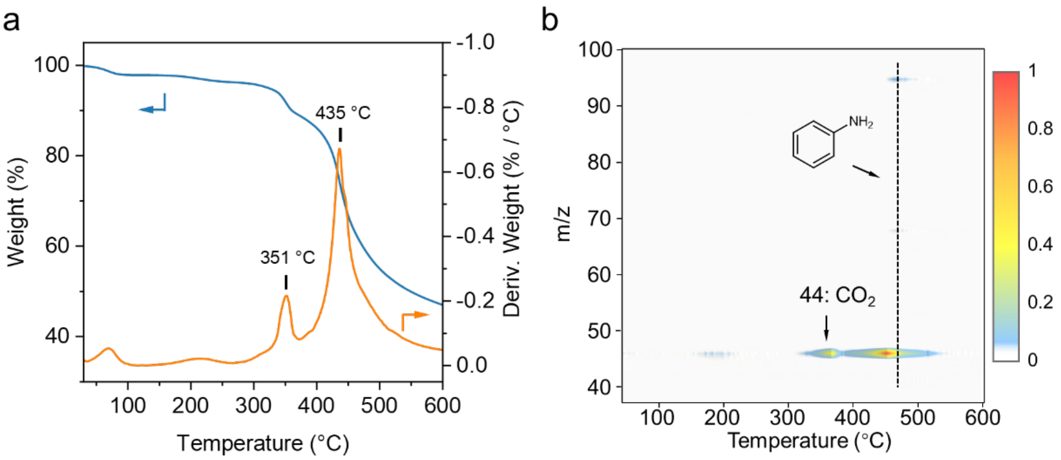

Figure S24. (a) The TG (yellow) and the first derivative curve (blue) for HOM-1 during calcination. (b) TG-MS spectra of thermal degradation of HOM-1.

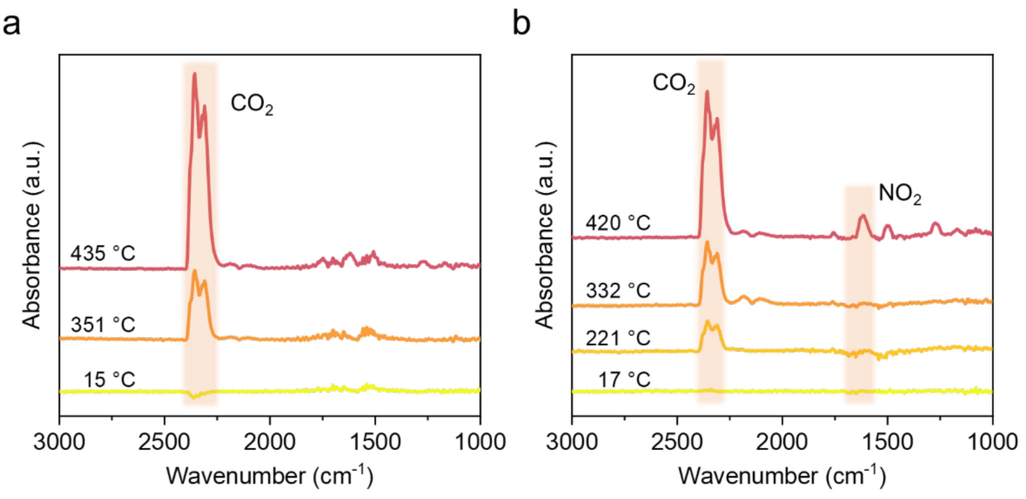

Figure S25. FT-IR spectra of gases produced during the pyrolysis of HOM-1 (a) and Pd-HOM-1 (b) at several characteristic temperatures.

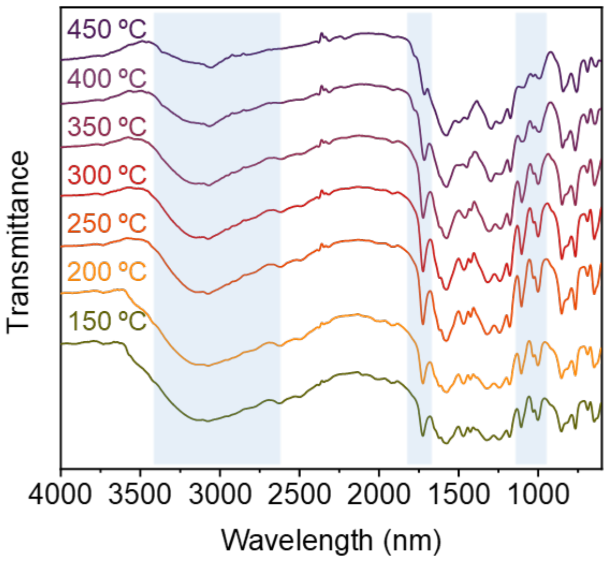

Figure S26. Variable-temperature FTIR spectra of HOM-1 in a nitrogen atmosphere (heating rate: 10 °C/min).

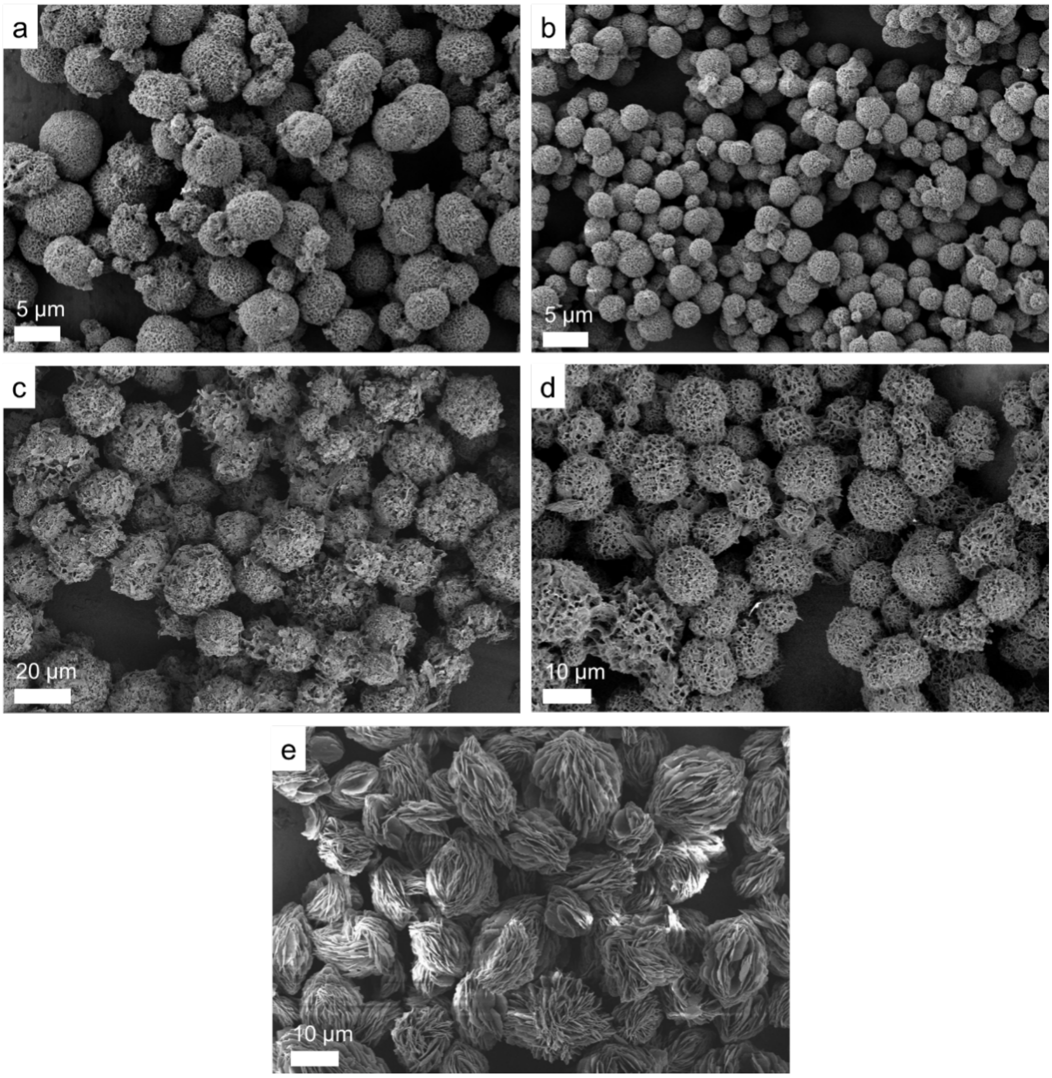

Figure S27. (a-c) SEM images for the precursor materials for various Pd-SMC-2~4 (before Pd loading and calcination). (d-e) SEM images for the precursor materials for various Pd-RMC-5~6 (before Pd loading and calcination). The detailed synthetic condition can be found in Supplementary Table 1.

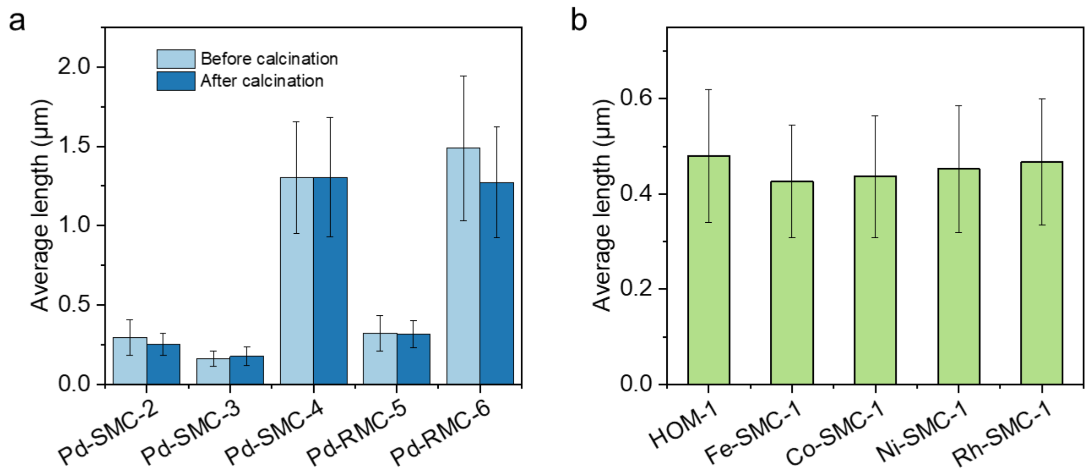

Figure S28. (a) Comparison of point spacing calculated based on Delaunay triangulation before and after calcination of the material. **b.** Comparison of point spacing between HOM-1 and M-SMC-1 loaded with different metals.

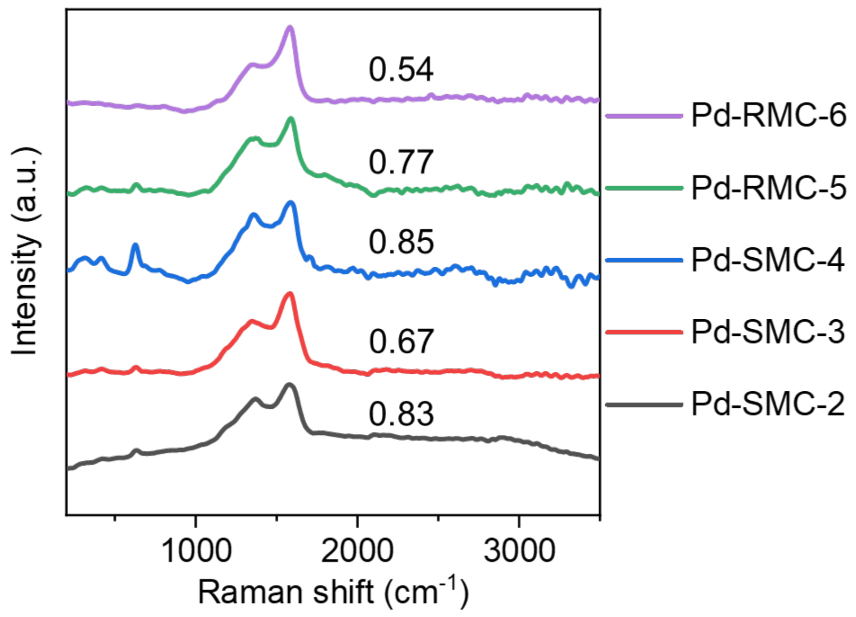

Figure S29. Raman spectra of Pd-MCs, the inserted values are I_D/I_G. All data were collected by a microscope system with a 532 nm laser as excitation light sources. The detailed test method can be found in the general information.

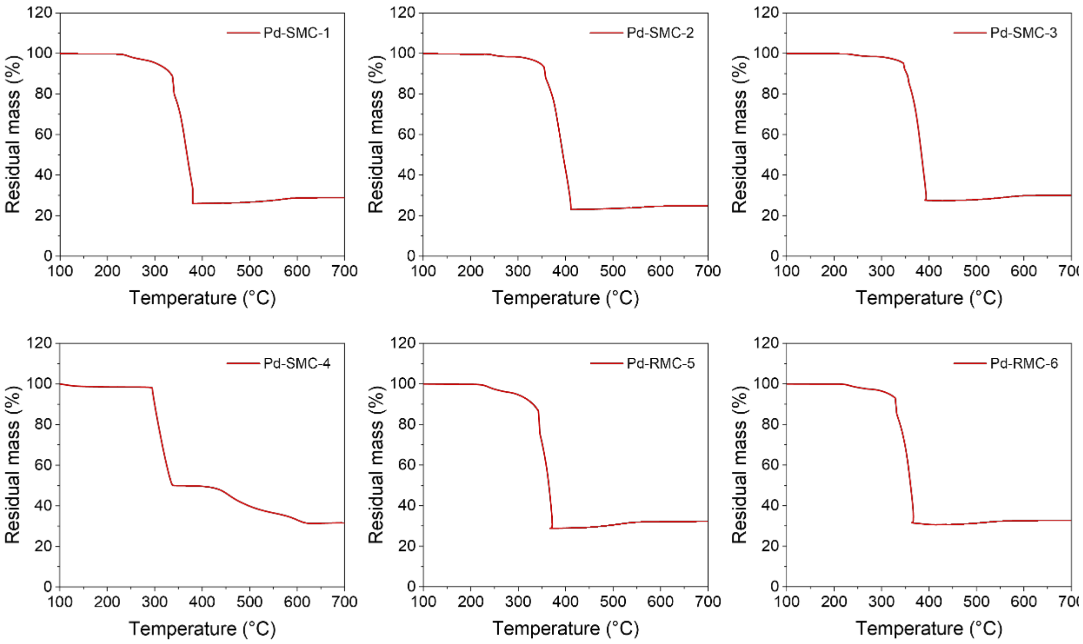

Figure S30. The thermogravimetric curves of Pd-MCs. All samples were dried in a vacuum oven at 40 °C and 1 bar for 12 hours before TG measurement. TG curves were collected in an air atmosphere with a heating rate of 10 °C min^{-1}.

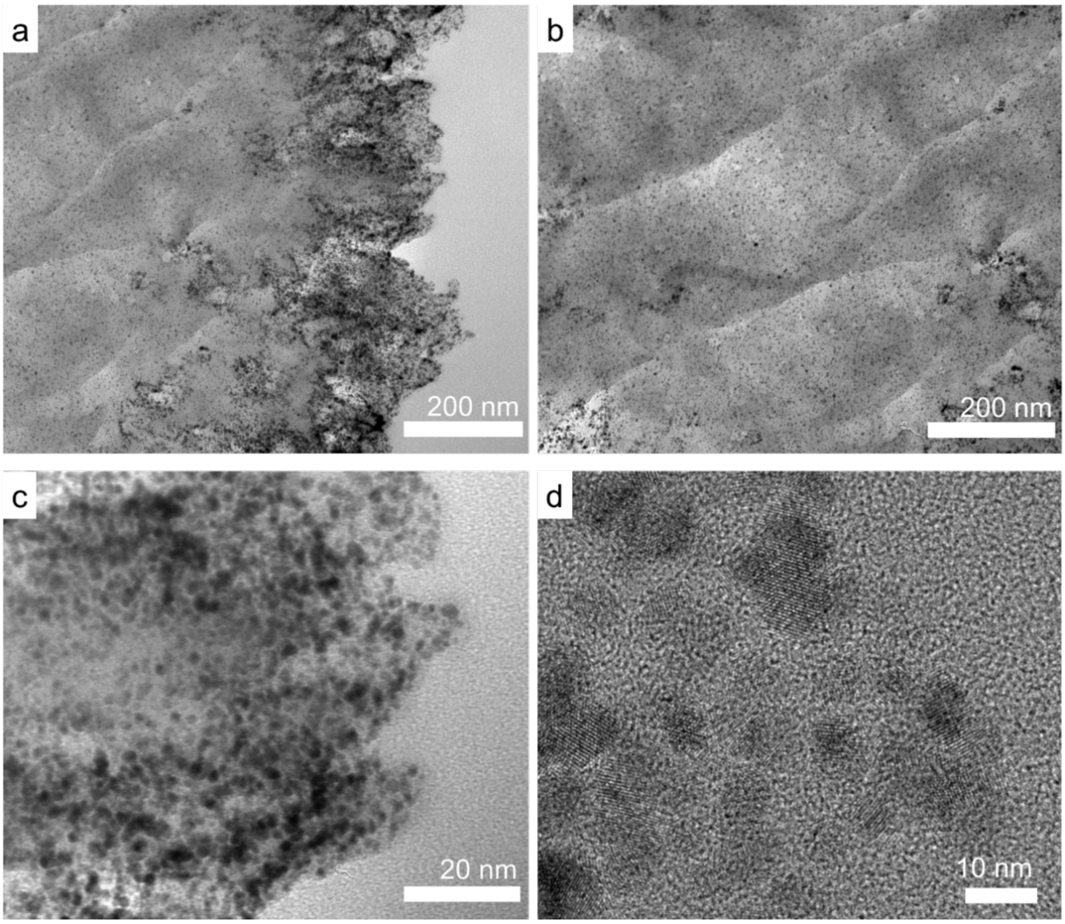

Figure S31. (a-b) TEM images of the ultrathin section of Pd-SMC-2. (c-d) HR-TEM images of the ultrathin section of Pd-SMC-2. The thickness of the slice is 100 nm.

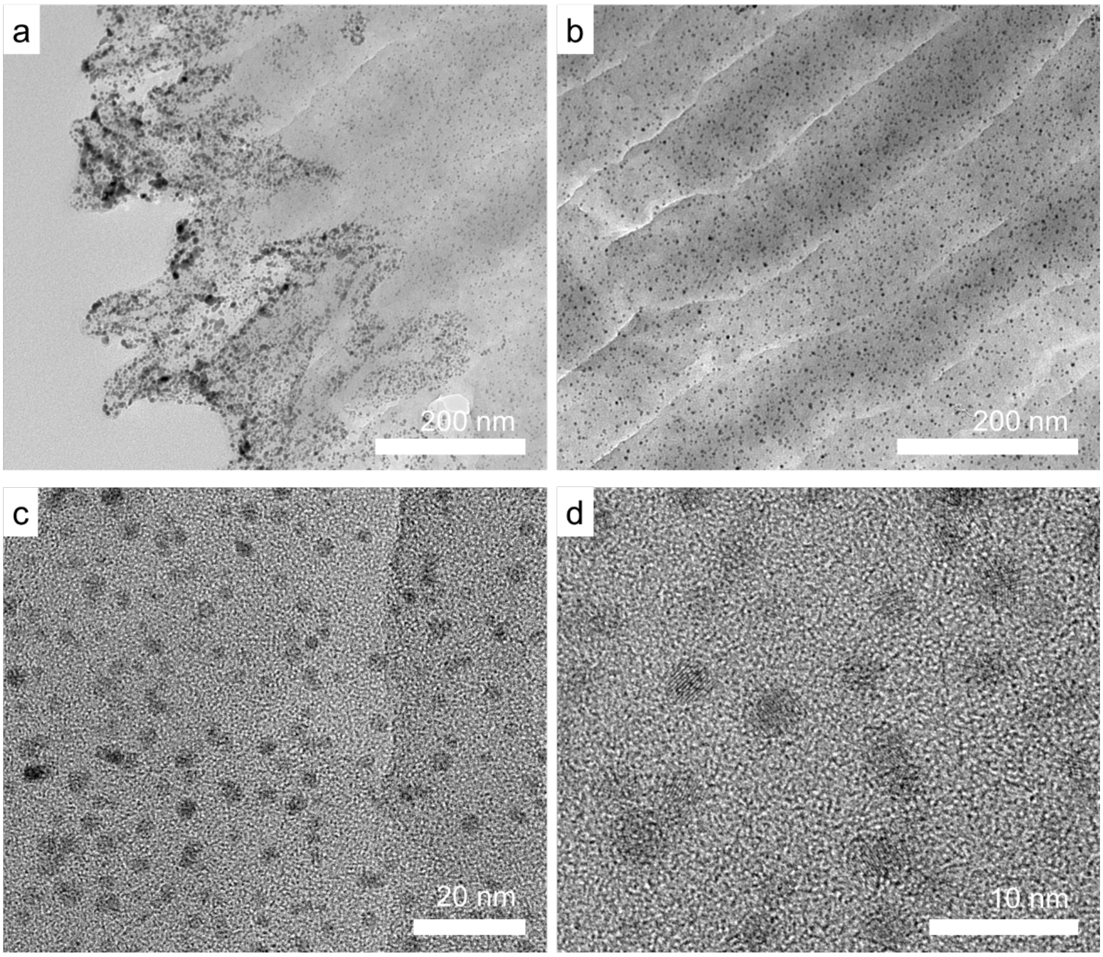

Figure S32. (a-b) TEM images of the ultrathin section of Pd-SMC-3. (c-d) HR-TEM images of the ultrathin section of Pd-SMC-3. The thickness of the slice is 100 nm.

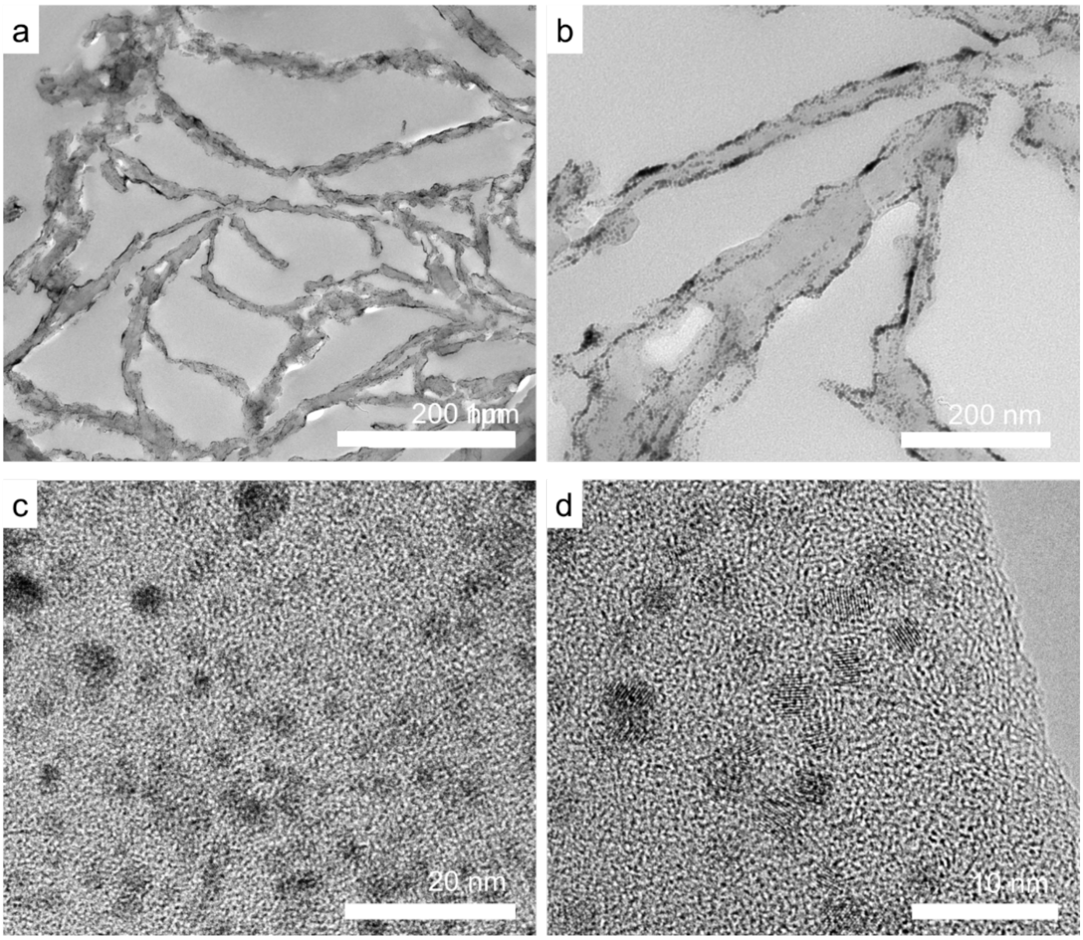

Figure S33. (a-b) TEM images of the ultrathin section of Pd-RMC-6. The thickness of the slice is 100 nm.

a

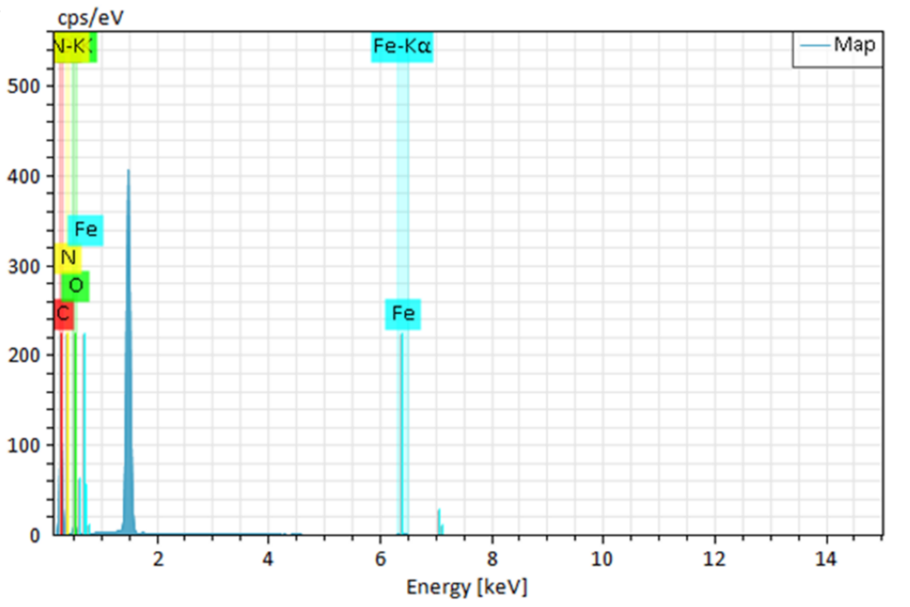

b

Element	Atom / %	Weight / %
O	14.87	17.66
C	74.54	66.45
N	9.02	9.37
Fe	1.57	6.52

Figure S34. (a) EDS spectrum of Fe-SMC-1. The peak around 1.5 keV originated from aluminum since all samples were dispersed on aluminum foil. The peak around 6.4 keV corresponds to the Kα emission of Fe. (b) Distribution of elements based on EDS calculation for Fe-SMC-1.

a

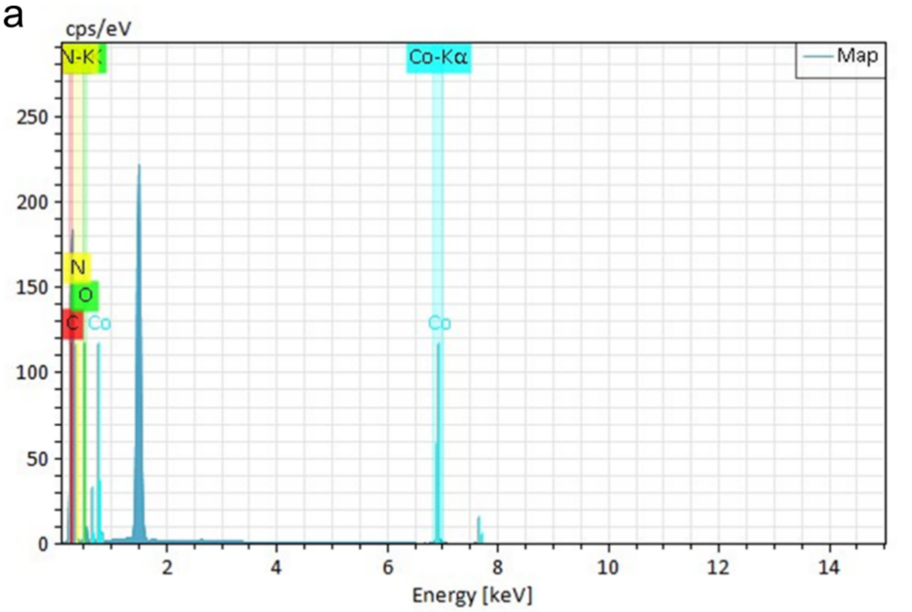

b

Element	Atom / %	Weight / %
O	9.67	11.72
C	80.87	73.59
N	8.08	8.58
Co	1.37	6.11

Figure S35. (a) EDS spectrum of Co-SMC-1. The peak around 1.5 keV originated from aluminum since all samples were dispersed on aluminum foil. The peak around 6.9 keV corresponds to the Kα emission of Co. (b) Distribution of elements based on EDS calculation for Co-SMC-1.

a

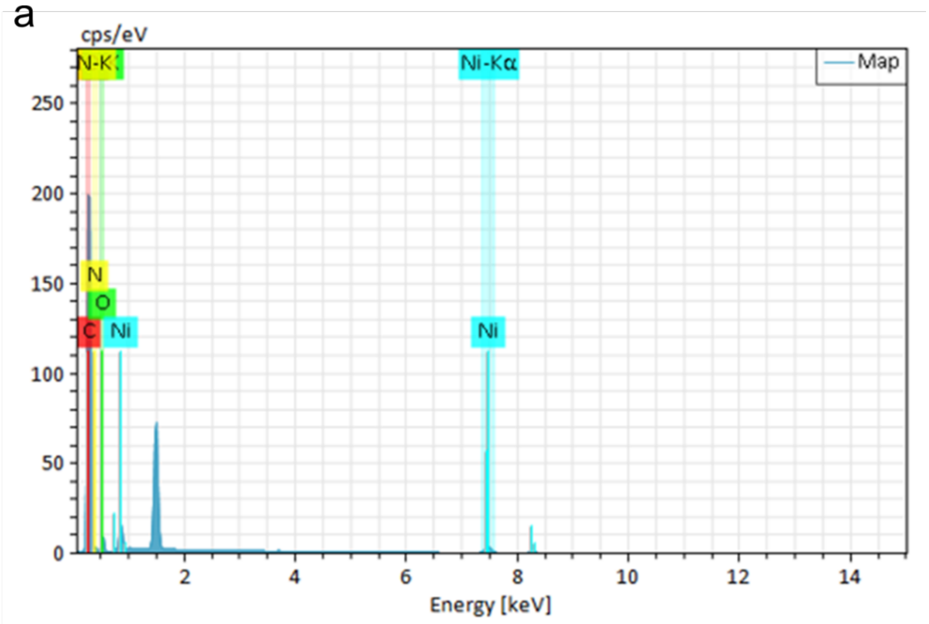

b

Element	Atom / %	Weight / %
O	7.65	9.18
C	82.27	74.13
N	8.26	8.68
Ni	1.82	8.01

Figure S36. (a) EDS spectrum of Ni-SMC-1. The peak around 1.5 keV originated from aluminum since all samples were dispersed on aluminum foil. The peak around 7.5 keV corresponds to the Kα emission of Ni. (b) Distribution of elements based on EDS calculation for Ni-SMC-1.

a

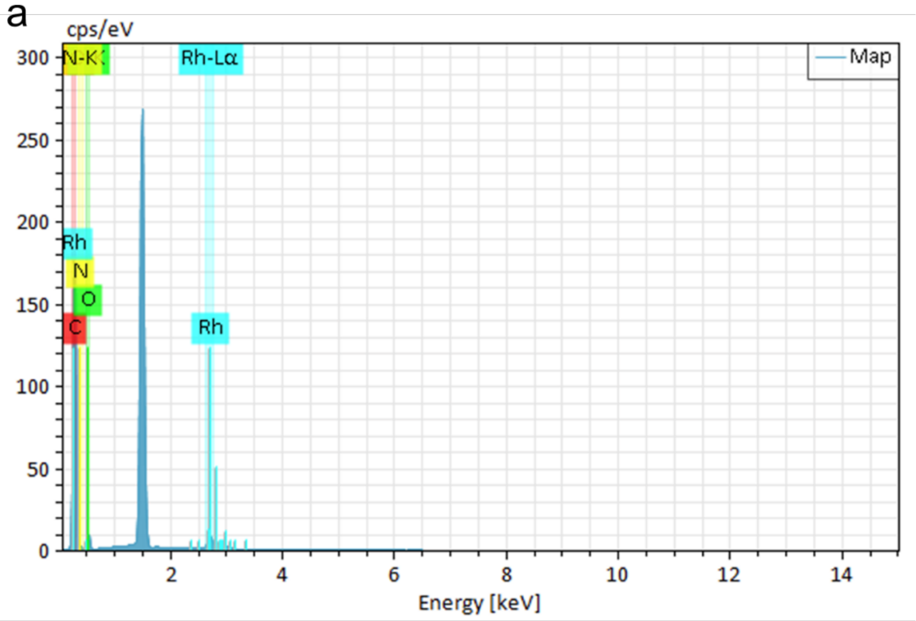

b

Element	Atom / %	Weight / %
O	10.12	11.71
C	81.28	70.57
N	7.19	7.28
Rh	1.41	10.45

Figure S37. (a) EDS spectrum of Rh-SMC-1. The peak around 1.5 keV originated from aluminum since all samples were dispersed on aluminum foil. The peak around 2.7 keV corresponds to the Lα emission of Rh. (b) Distribution of elements based on EDS calculation for Rh-SMC-1.

a

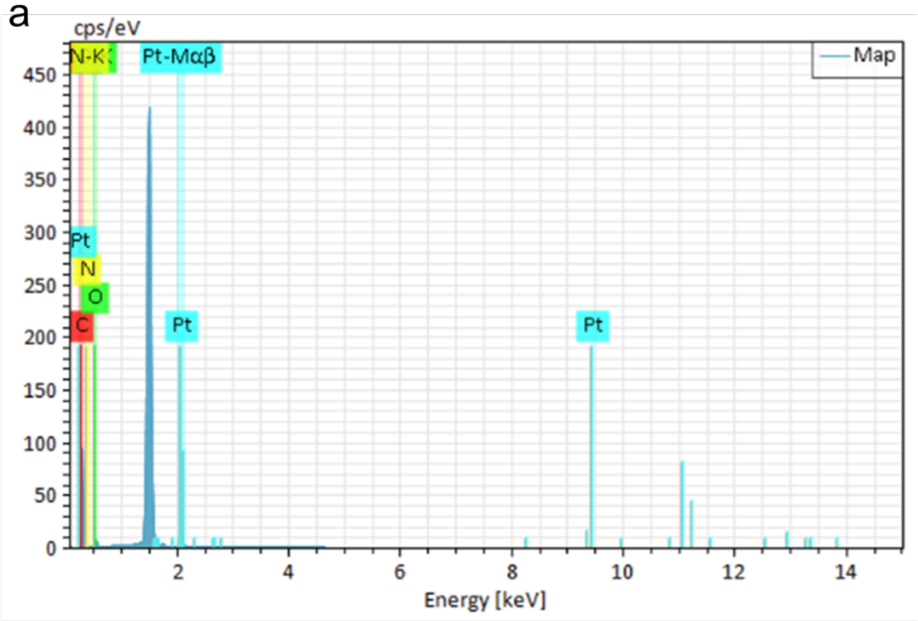

b

Element	Atom / %	Weight / %
O	9.72	12.06
C	82.60	76.97
N	7.50	8.15
Pt	0.19	2.81

Figure S38. (a) EDS spectrum of Pt-SMC-1. The peak around 1.5 keV originated from aluminum since all samples were dispersed on aluminum foil. The peak around 2.0 keV corresponds to the Mα emission of Ru. (b) Distribution of elements based on EDS calculation for Pt-SMC-1.

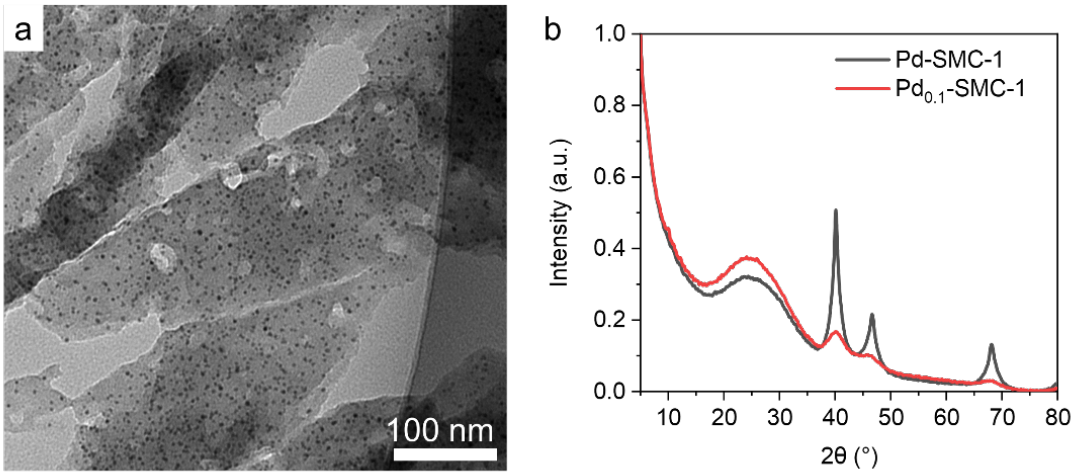

Figure S39. a. TEM image for the ultrathin section of $Pd_{0.1}$-SMC-1. **b.** Comparison of PXRD patterns of $Pd_{0.1}$-SMC-1 and Pd-SMC-1.

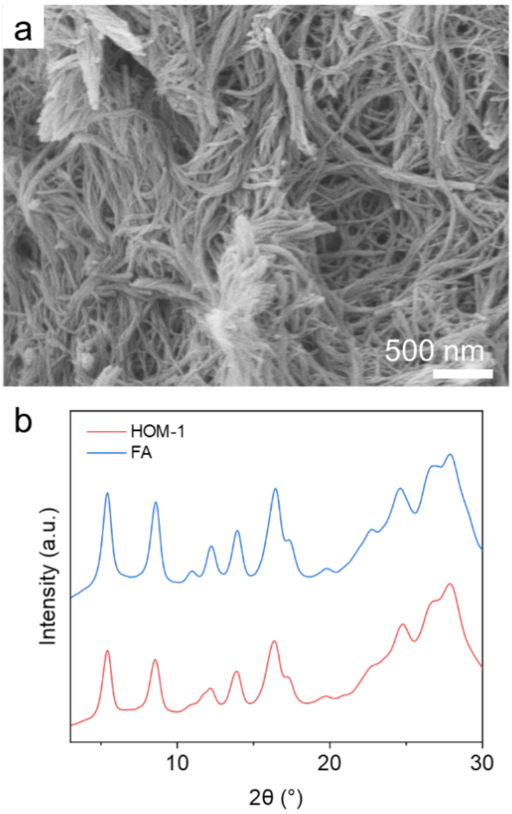

Figure S40. (a) The SEM image for fibrous assembly (FA). (b) The comparison of PXRD patterns for HOM-1 and FA.

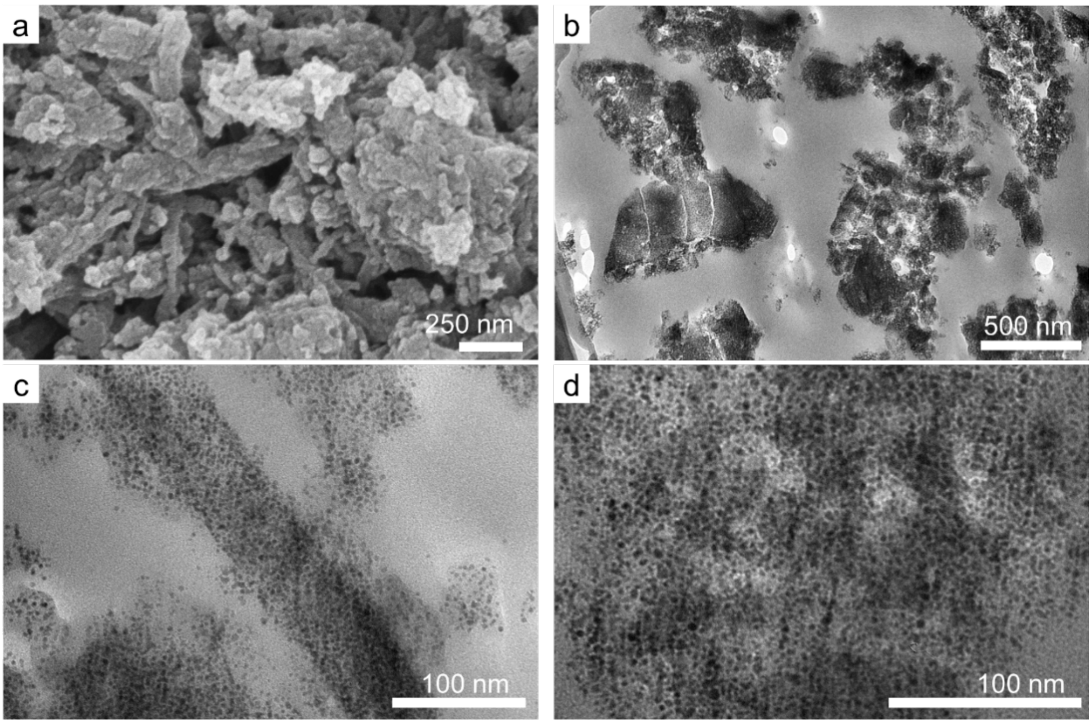

Figure S41. (a) The SEM image for Pd-BAC. The fibrous HOM-1 was obtained by the condensation and precipitation of 2,4,6-trihydroxybenzene-1,3,5-tricarbaldehyde and 4-aminobenzoic acid in a mixed solvent of DMSO and water. The molar ratios of the precursors and the solvent volume were identical to those used for HOM-1. (b-d) The TEM image for the ultrathin-section of Pd-BAC. The thickness of the slice is 100nm.

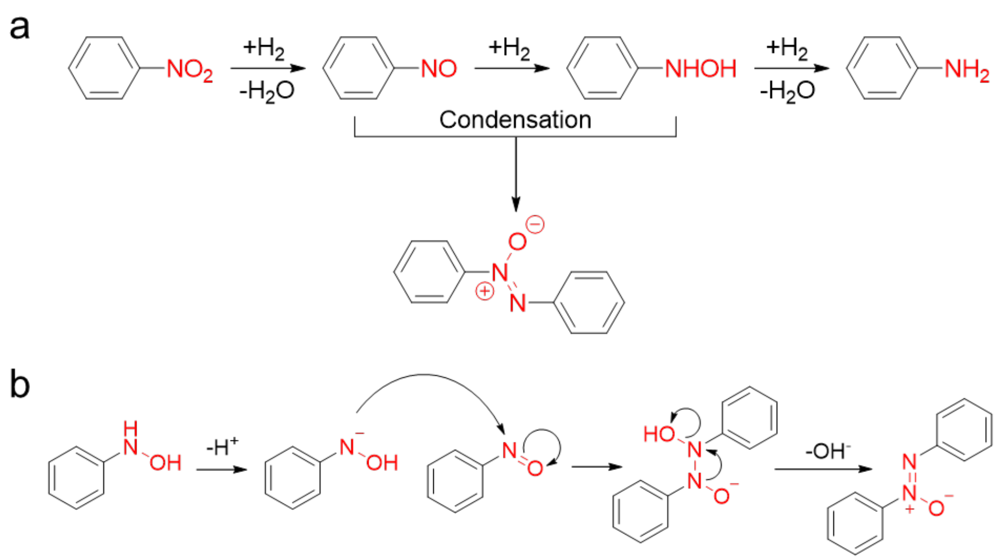

Figure S42. a. Reaction pathway of the hydrogenation of nitroaromatics, exemplified by nitrobenzene. **b.** Condensation of nitrosobenzene with N-phenylhydroxylamine under alkaline conditions.

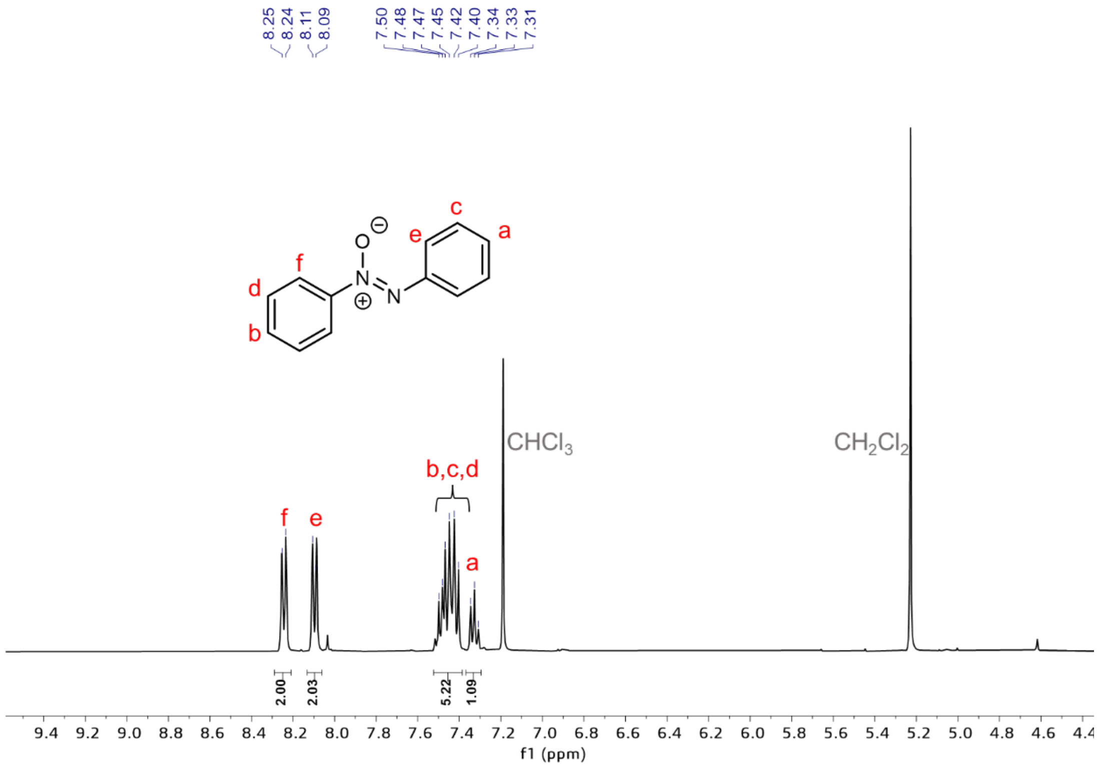

Figure S43. ^{1}H-NMR spectrum of the selective hydrogenation products of nitrobenzene in EDA.

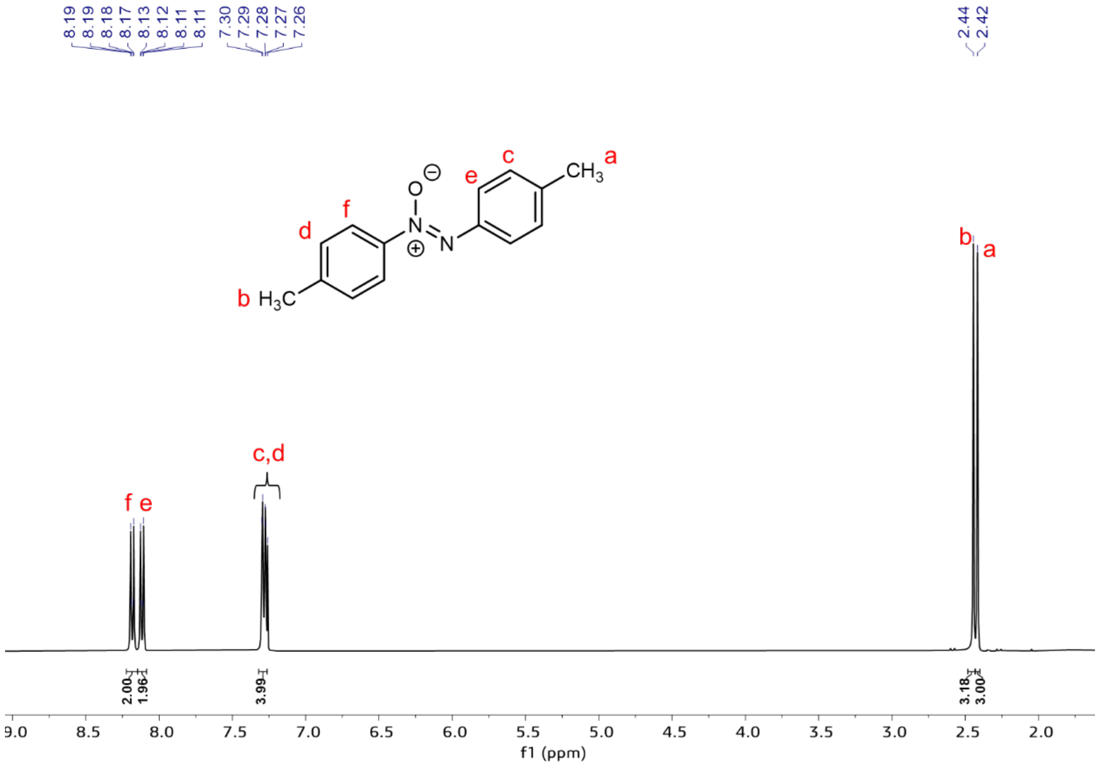

Figure S44. ^{1}H-NMR spectrum of the selective hydrogenation products of p-toluidine in EDA.

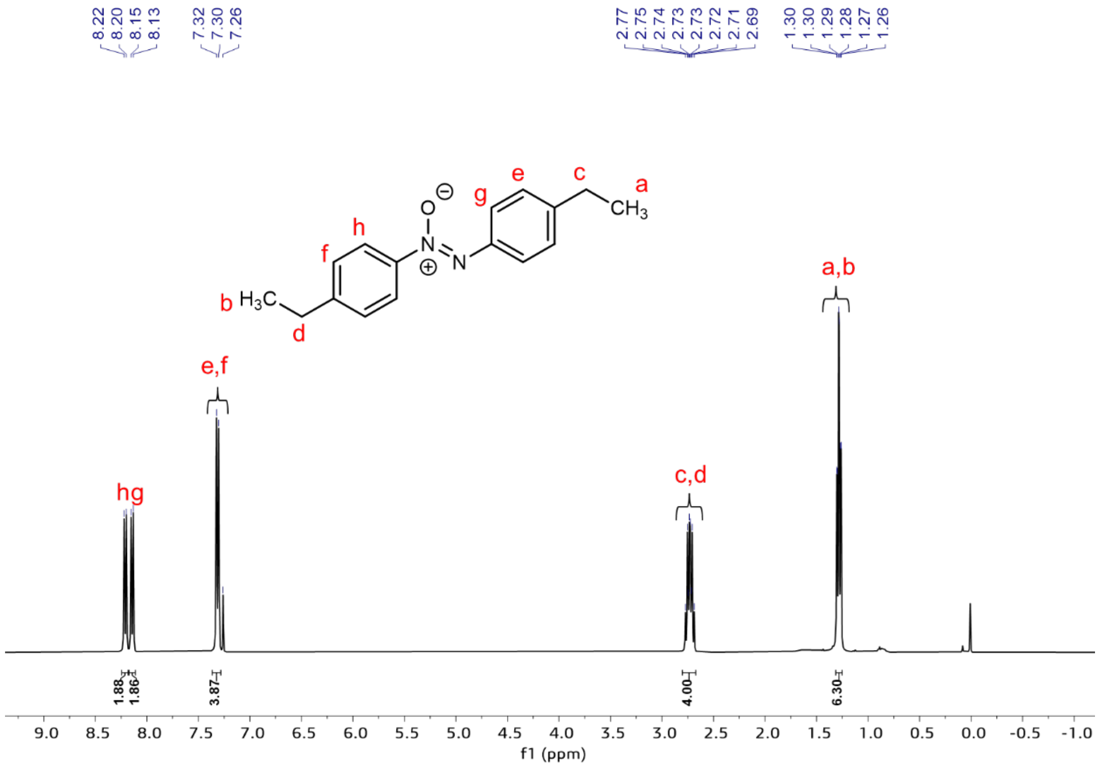

Figure S45. ^{1}H-NMR spectrum of the selective hydrogenation products of 4-ethylaniline in EDA.

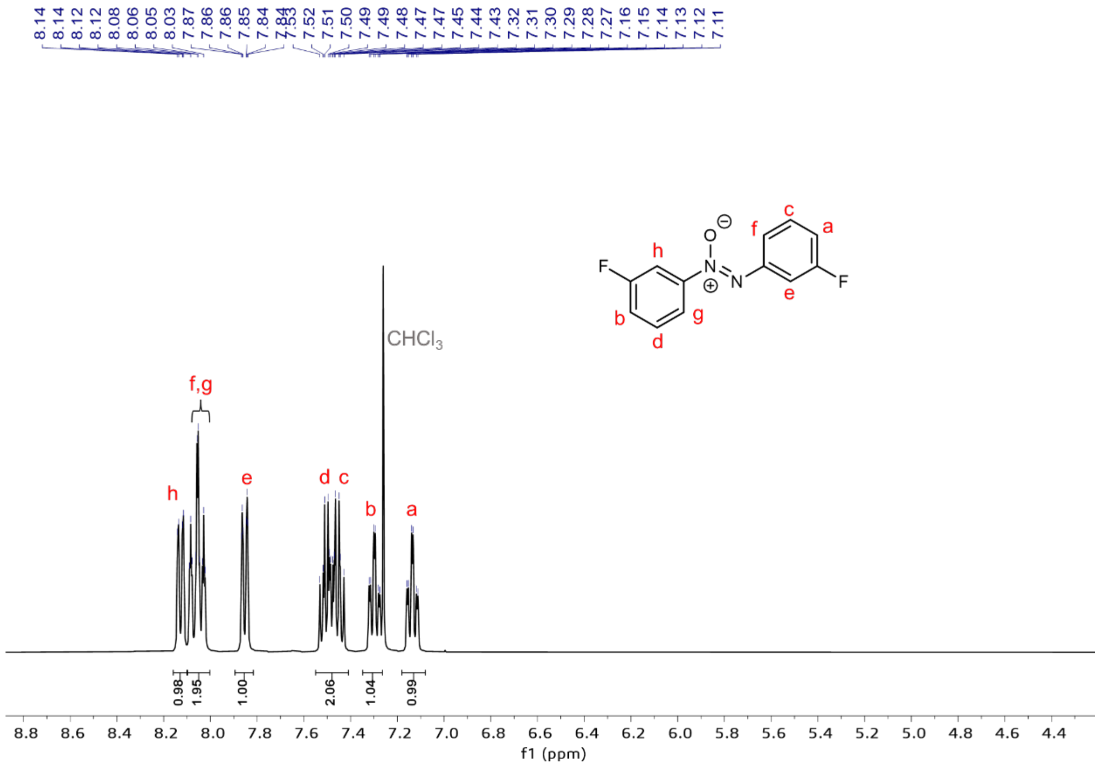

Figure S46. ^{1}H-NMR spectrum of the selective hydrogenation products of 3-fluoroaniline in EDA.

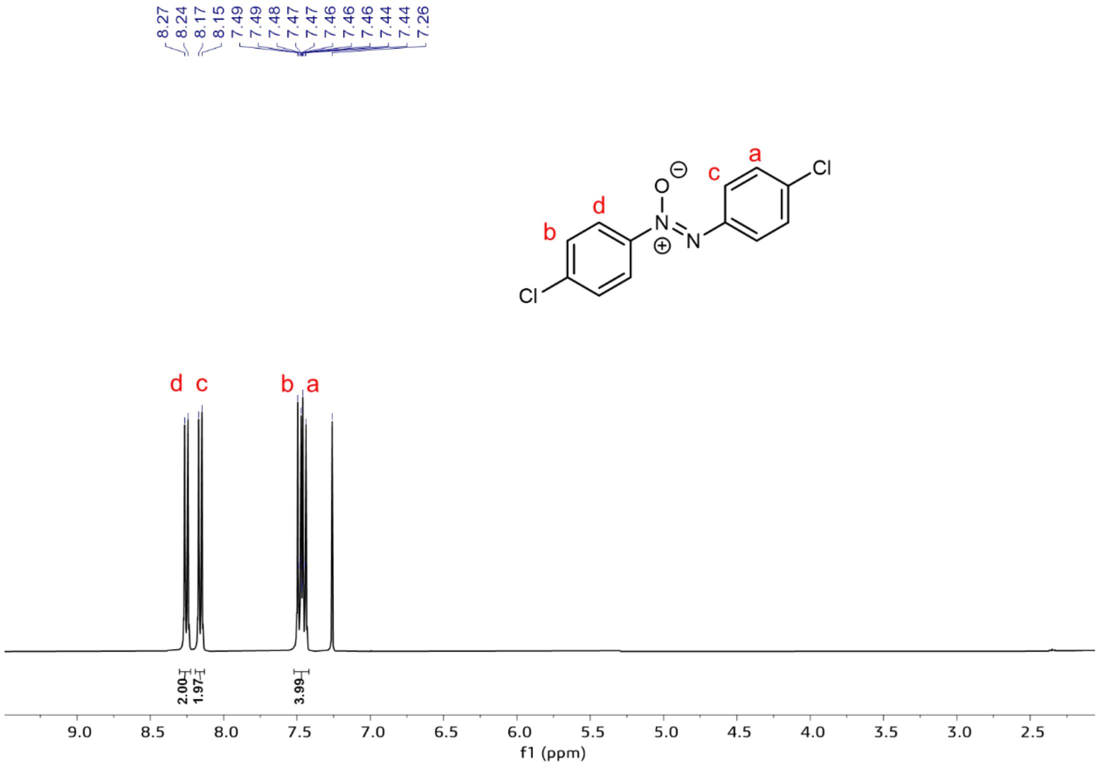

Figure S47. ^{1}H-NMR spectrum of the selective hydrogenation products of 4-chloroaniline in EDA.

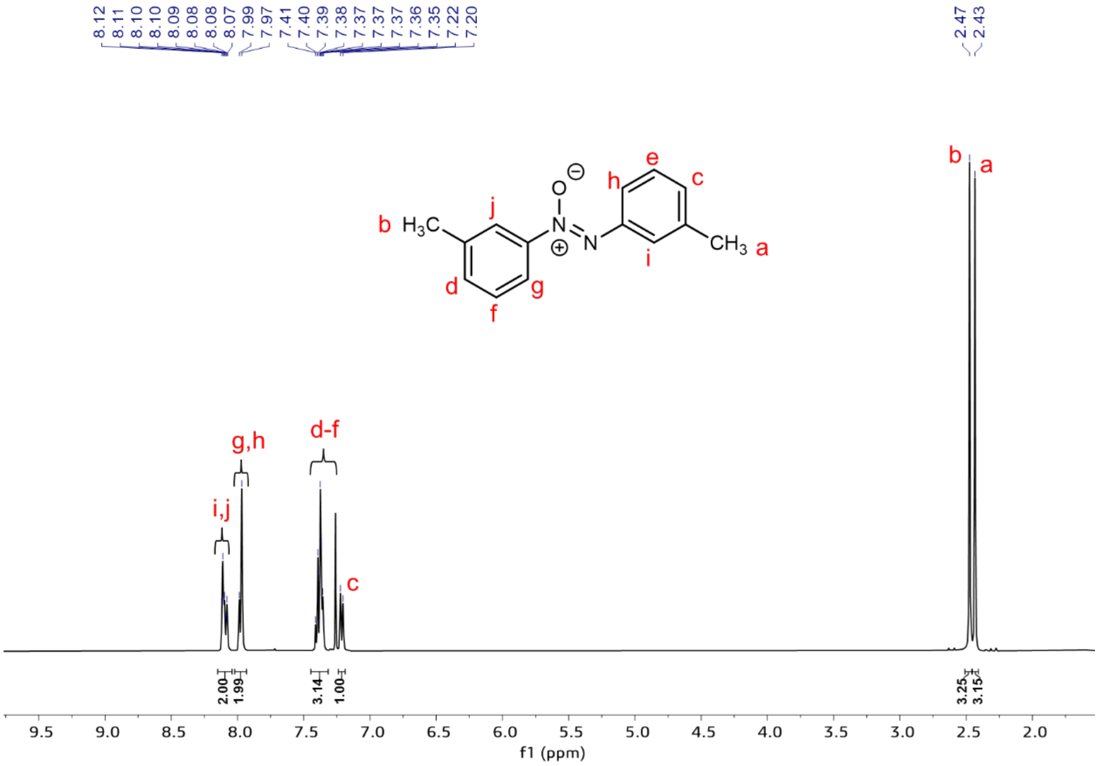

Figure S48. ^{1}H-NMR spectrum of the selective hydrogenation products of m-toluidine in EDA.

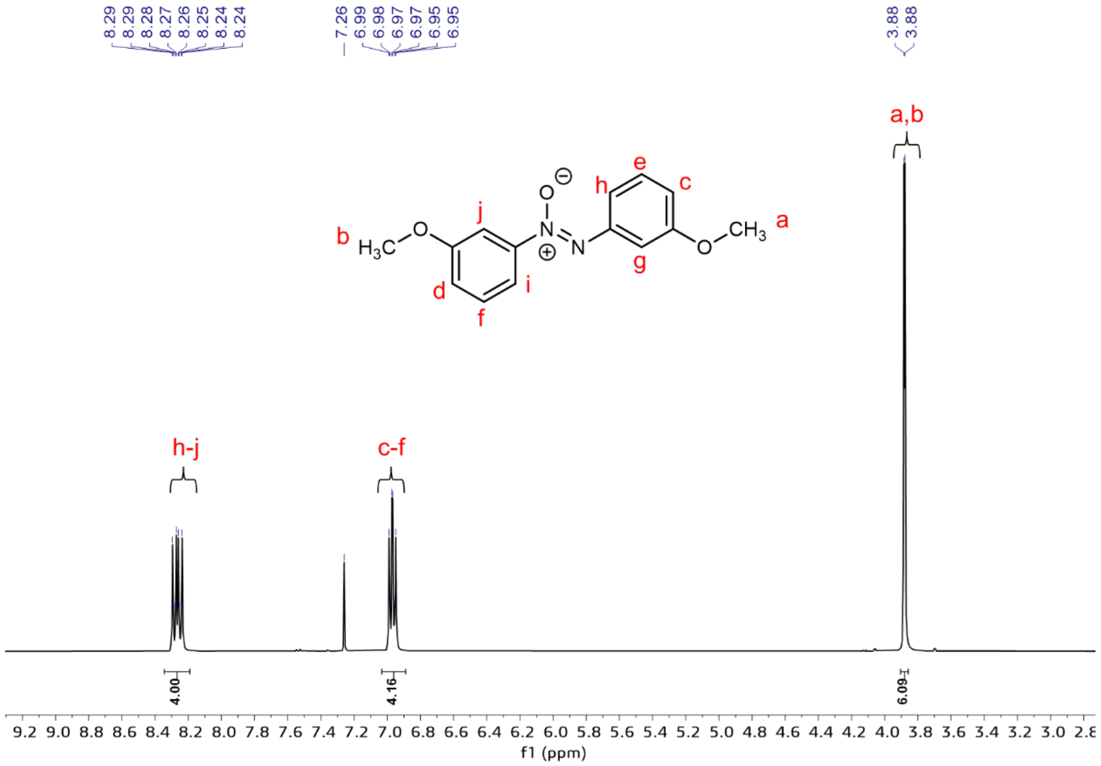

Figure S49. ^{1}H-NMR spectrum of the selective hydrogenation products of 3-methoxyaniline in EDA.

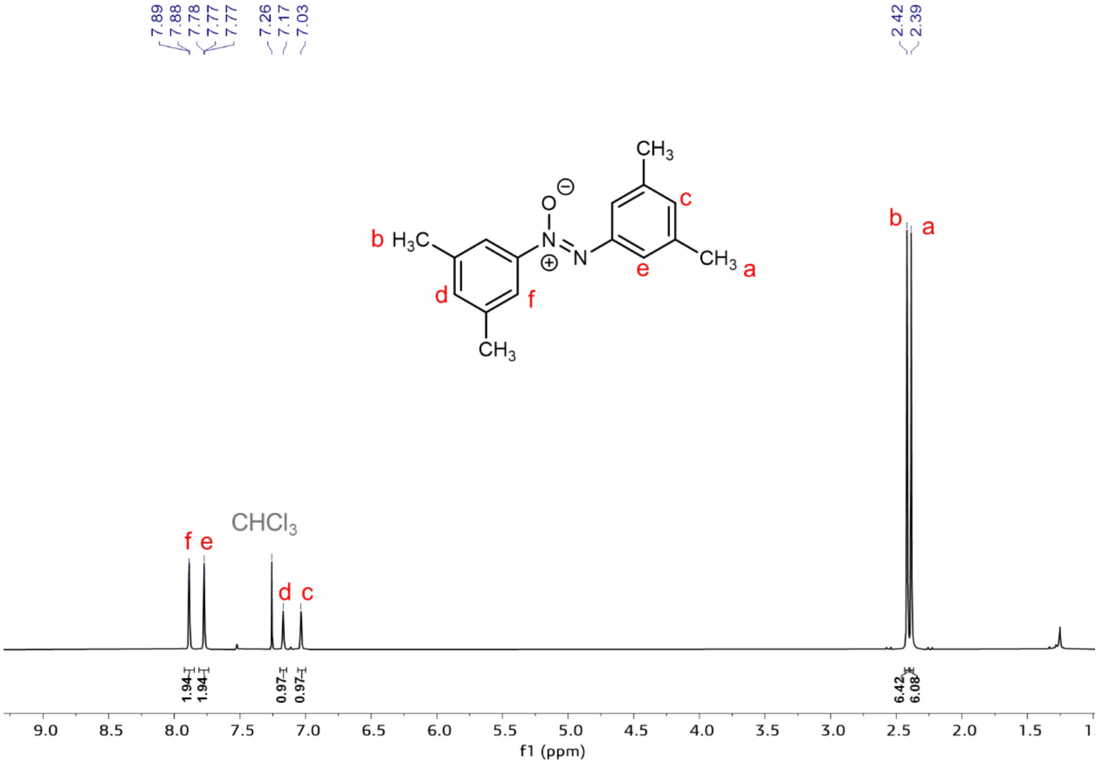

Figure S50. ^{1}H-NMR spectrum of the selective hydrogenation products of 3,5-dimethylaniline in EDA.

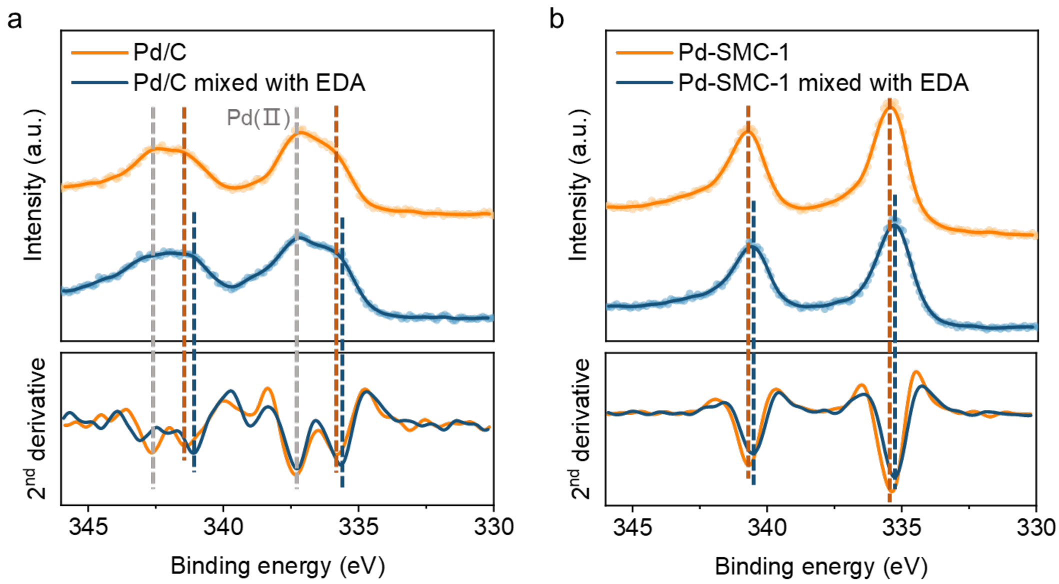

Figure S51. (a) XPS spectra of Pd 3d specimens for Pd/C compared with the sample treated by EDA. The bottom is the second-derivative spectra. The grey lines indicate the signal of Pd(II) in Pd/C. (b) XPS spectra of Pd 3d specimens for Pd-SMC-1 compared with the sample treated by EDA. The bottom is the second-derivative spectra.

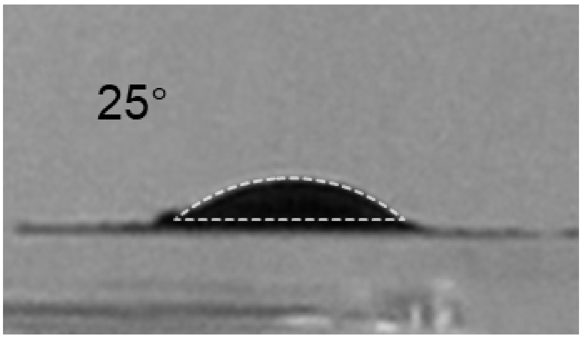

Figure S52. EDA contact angle over Pd-SMC-1.

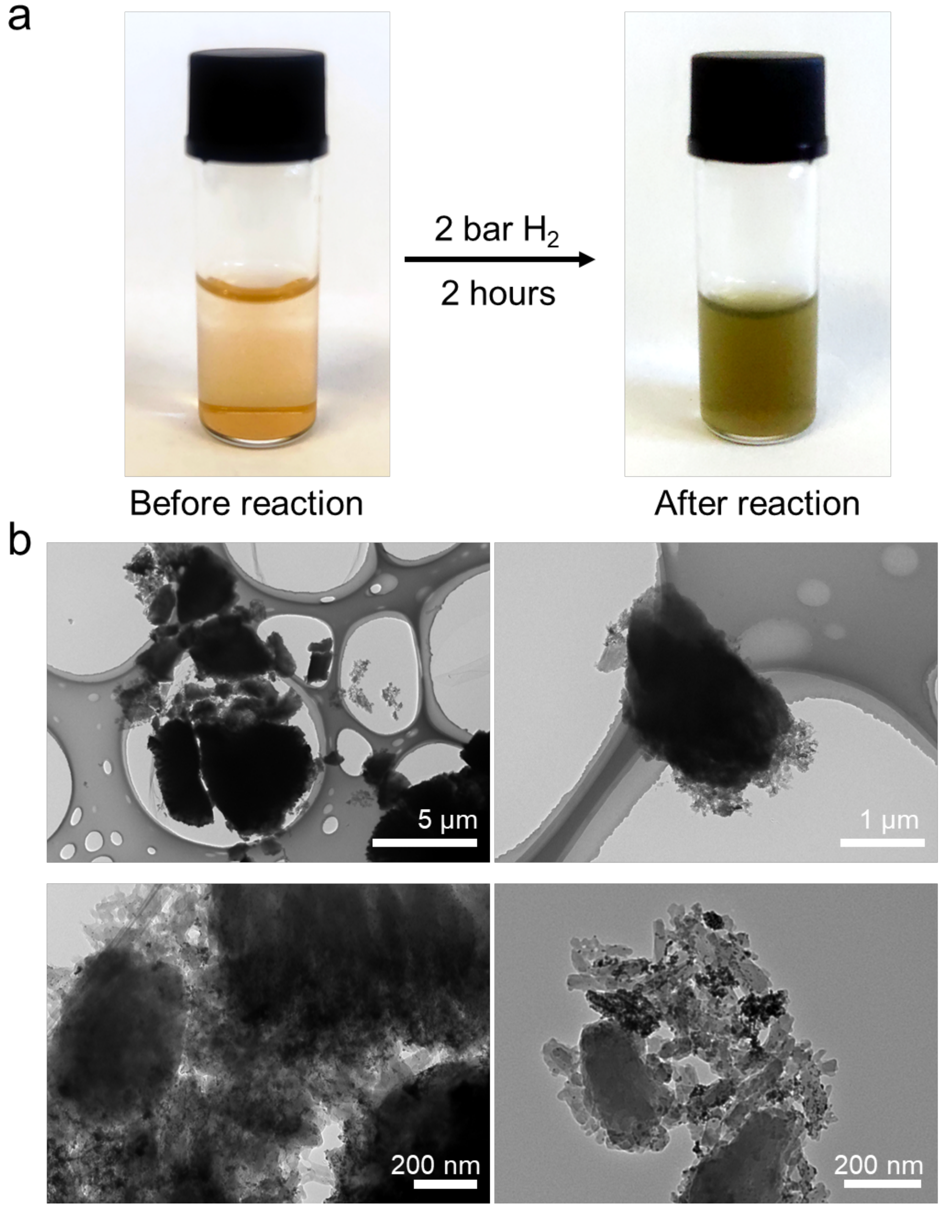

Figure S53. (a) Photos of the Pd-HOM-1 before and after reaction. (b) TEM images of Pd-HOM-1 after reaction. After the reaction, the original spherical morphology of Pd-HOM-1 disappeared, collapsing into aggregated fragments of varying sizes. Meanwhile, Pd nanoparticles with a broad size distribution were observed within the structure.

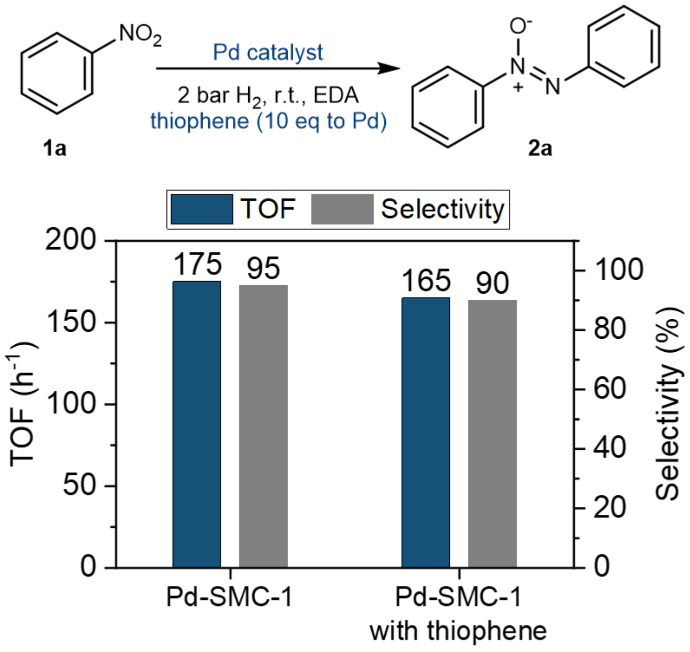

Figure S54. Comparison of catalytic performance of Pd catalyst with thiophene.

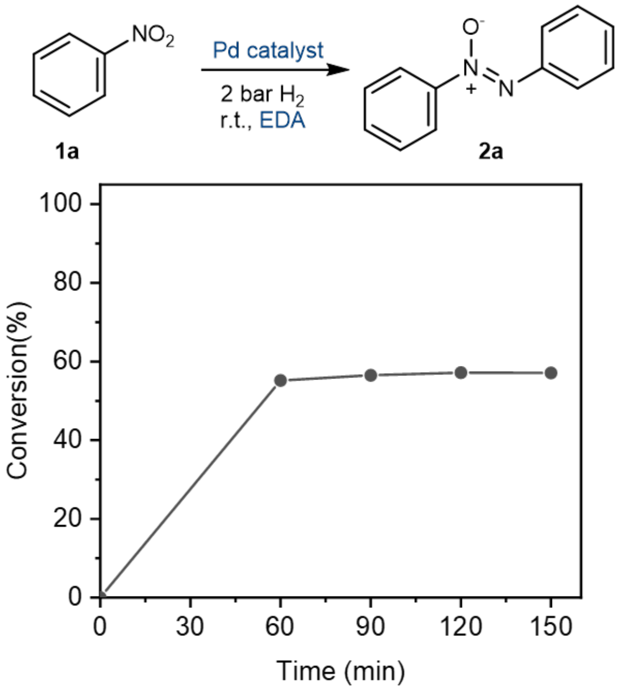

Figure S55. Hot filtration test for Pd-SMC-1 over the hydrogenation of nitrobenzene in EDA.

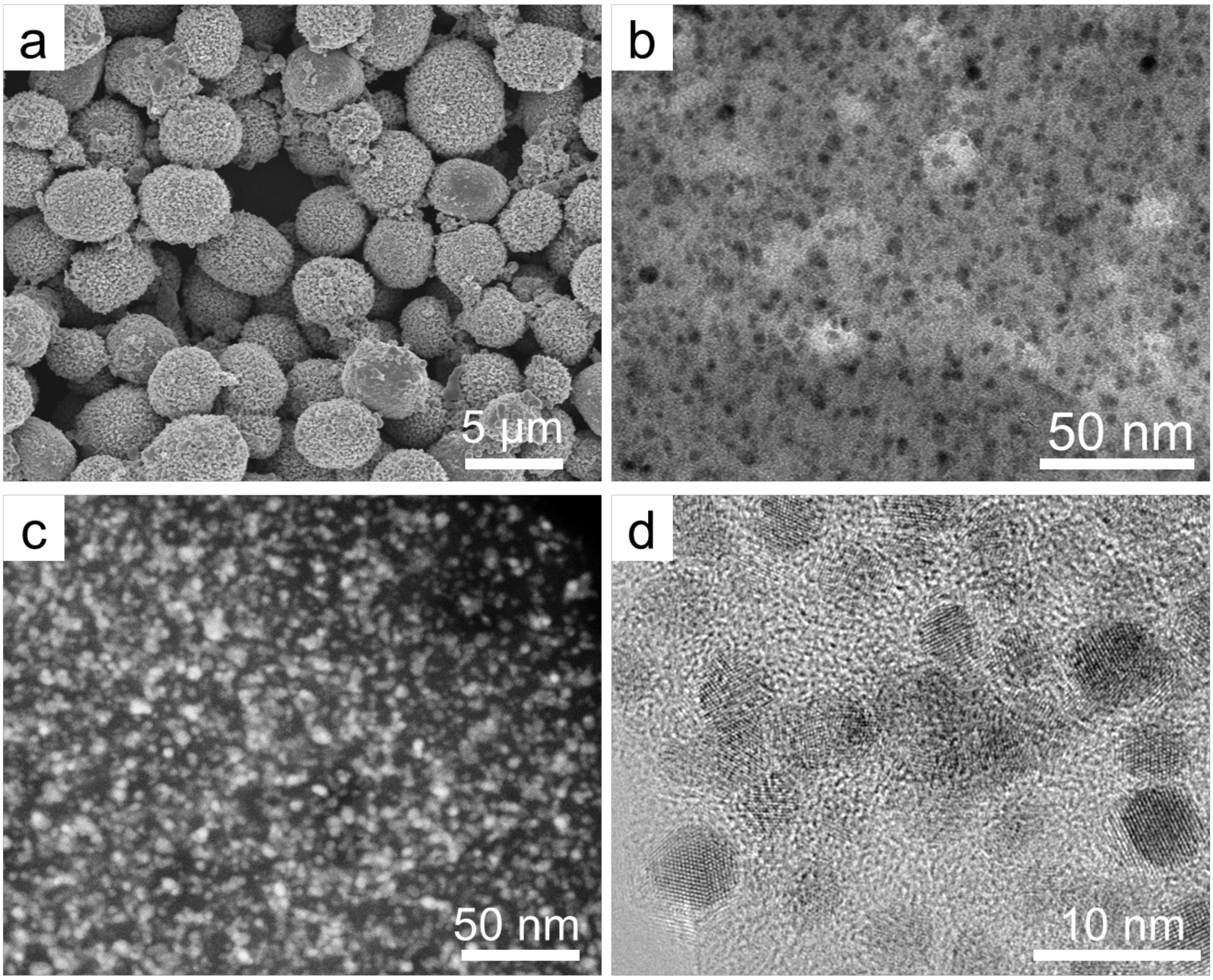

Figure S56. (a) SEM image of ultrathin section for Pd-SMC-1 after 5 cycles of reaction. (b) TEM image of ultrathin section for Pd-SMC-1 after 5 cycles of reaction. (c) STEM image of ultrathin section of Pd-SMC-1 after reaction. (d) HR-TEM images of Pd nanoparticles within Pd-SMC-1 after reaction.

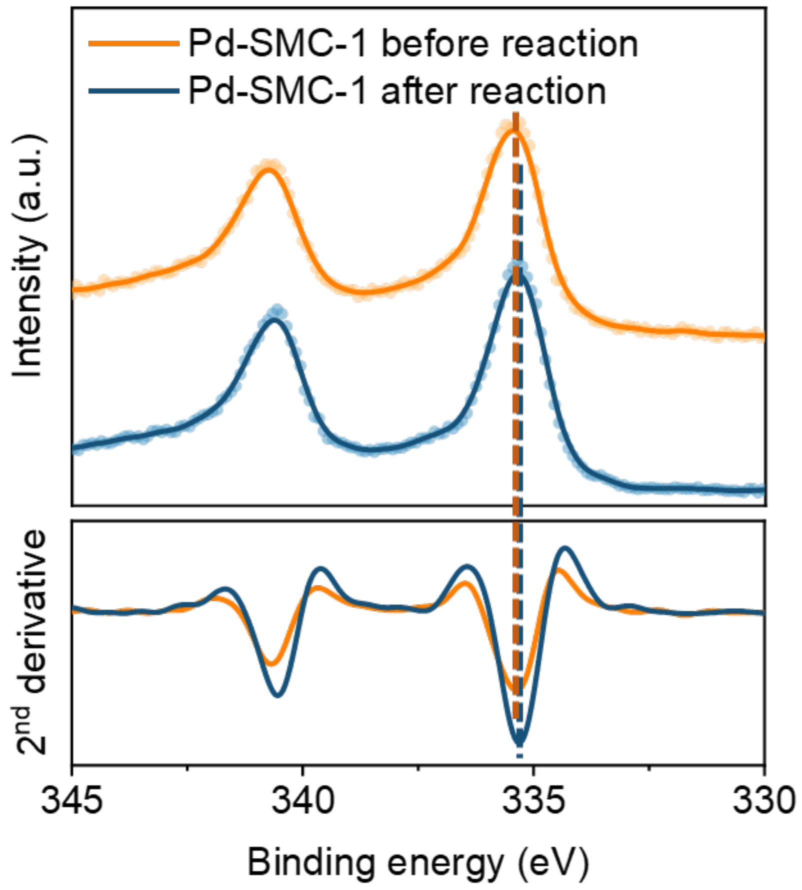

Figure S57. XPS spectra of Pd 3d specimens for Pd-SMC-1 before and after the hydrogenation of nitrobenzene in EDA.

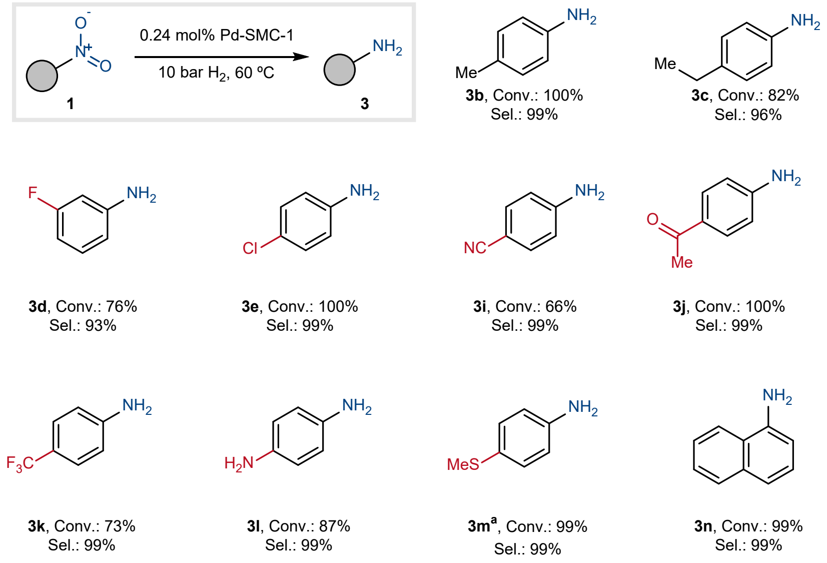

Figure S58. Substrate scope for selective hydrogenation of nitrobenzene in H_2O. Reaction condition: 400 µL of H_2O, 60 °C, 12 h, 10 bar H_2, 15 µL nitrobenzene, 0.24 mol% Pd. [a] 24 h of reaction time.

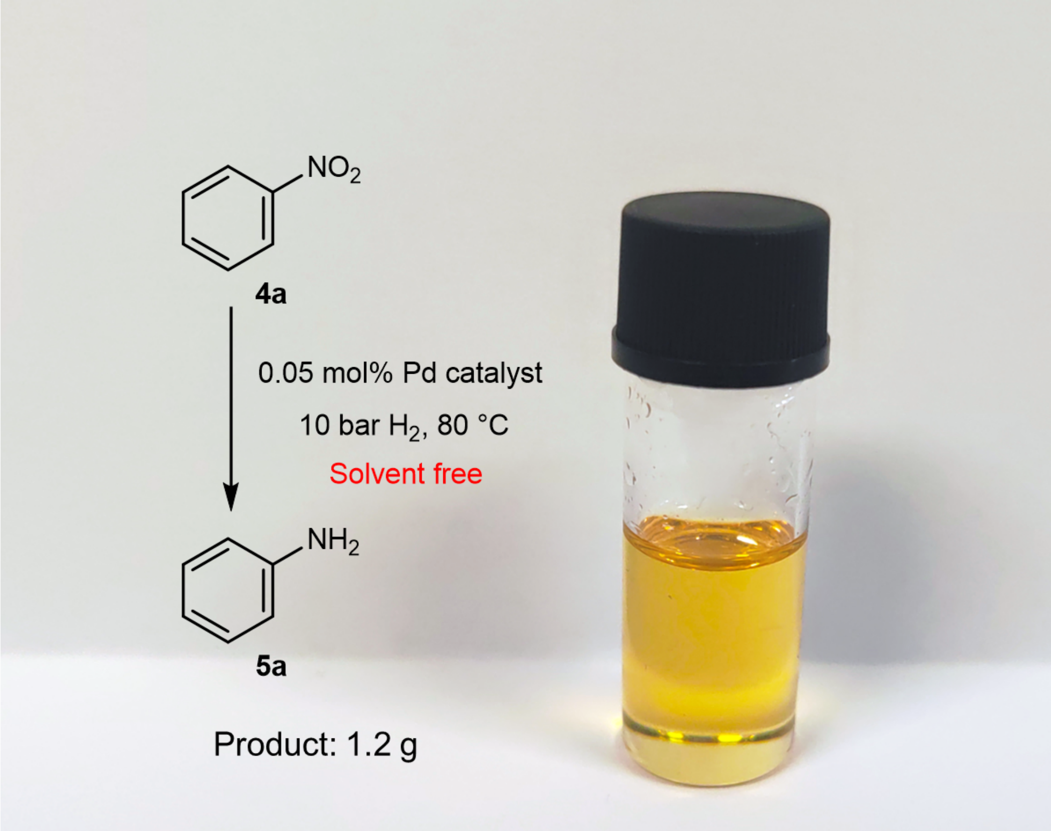

Figure S59. Isolated phenethylamine product from gram-scale solvent-free hydrogenation. The reaction was conducted with nitrobenzene (1.5 mL) and Pd-SMC-1 (0.5 mol%) under 10 bar H_2 at 80°C for 36 h. The crude mixture was purified via silica gel column chromatography (hexane/ethyl acetate = 5:1, v/v) to afford aniline as a yellow oil.

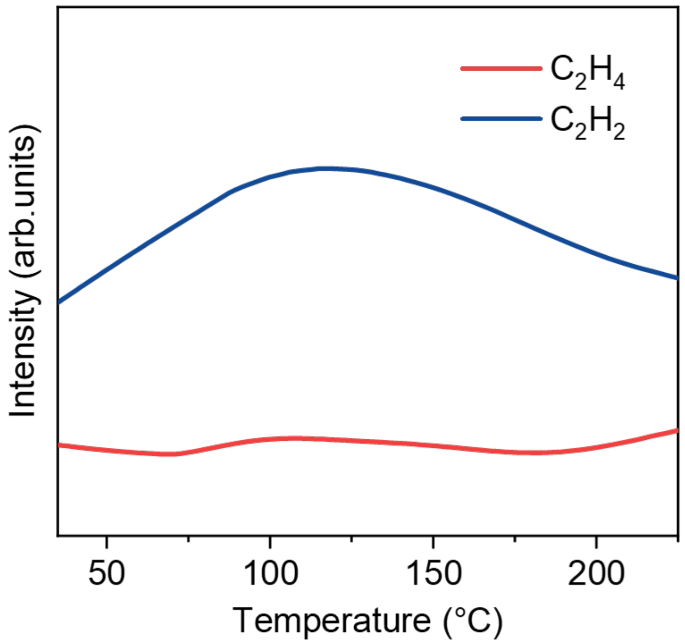

Figure S60. Temperature-programmed desorption experiments of C_2H_2 and C_2H_4 over Pd-SMC-1. The desorption of absorbed gas was carried out under a He atmosphere, with a heating rate of 10 °C/min up to 500 °C, and monitored by mass spectrometry.

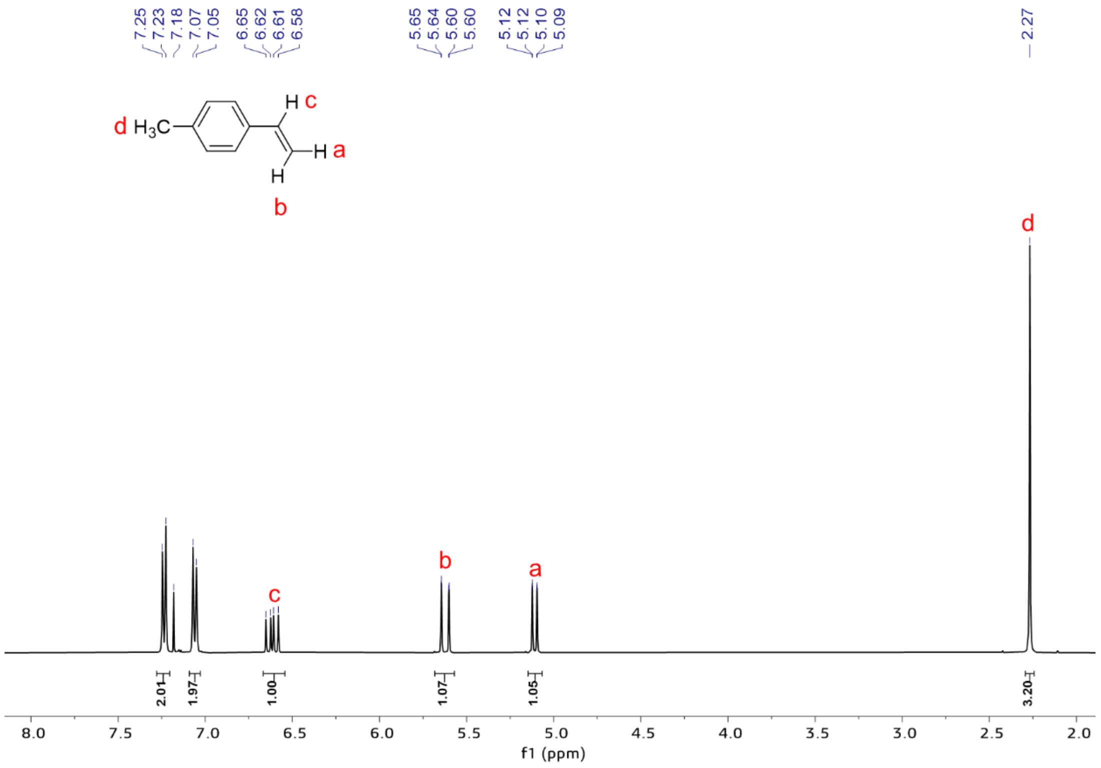

Figure S61.^{1}H-NMR spectrum of the selective hydrogenation products of 4-methylphenylene.

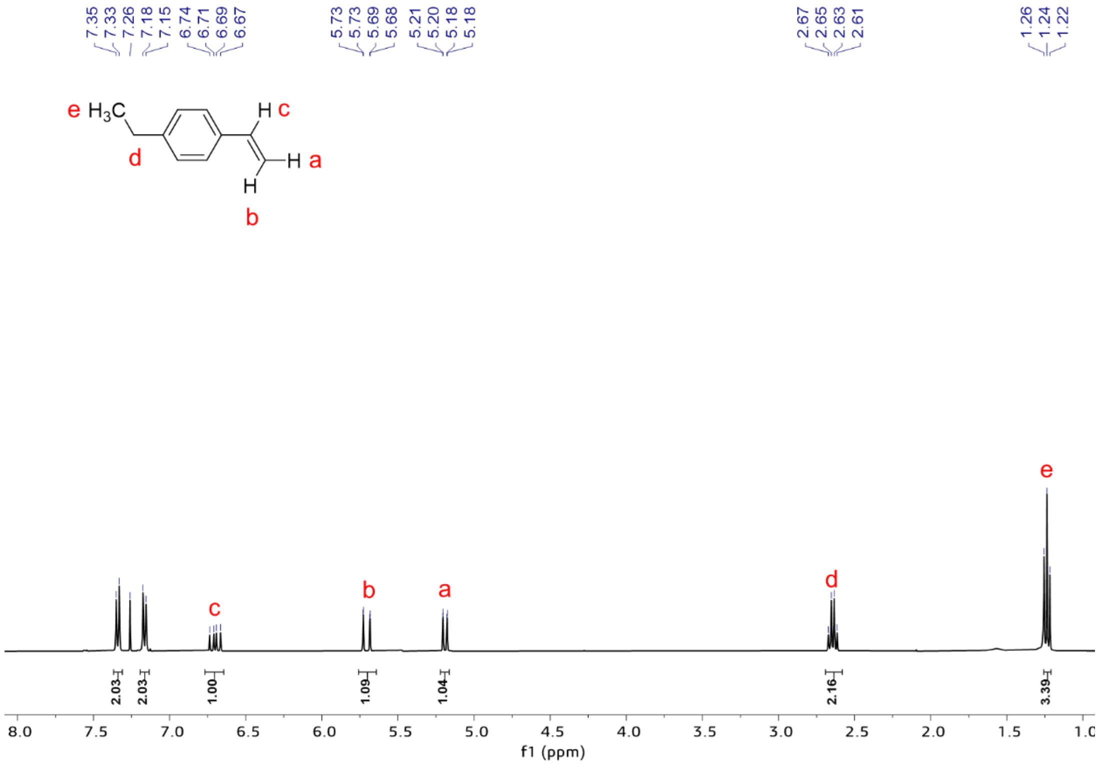

Figure S62. ^{1}H-NMR spectrum of the selective hydrogenation products of 4-ethylstyrene.

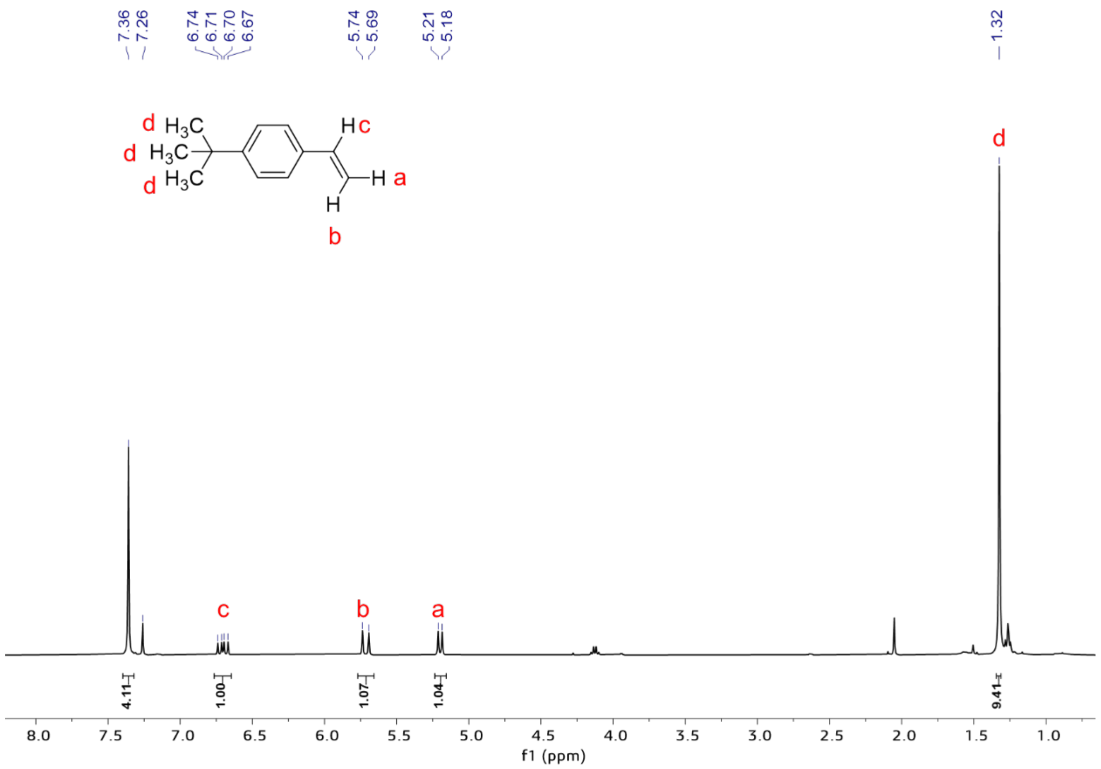

Figure S63. ^{1}H-NMR spectrum of the selective hydrogenation products of 4-tert-butylstyrene.

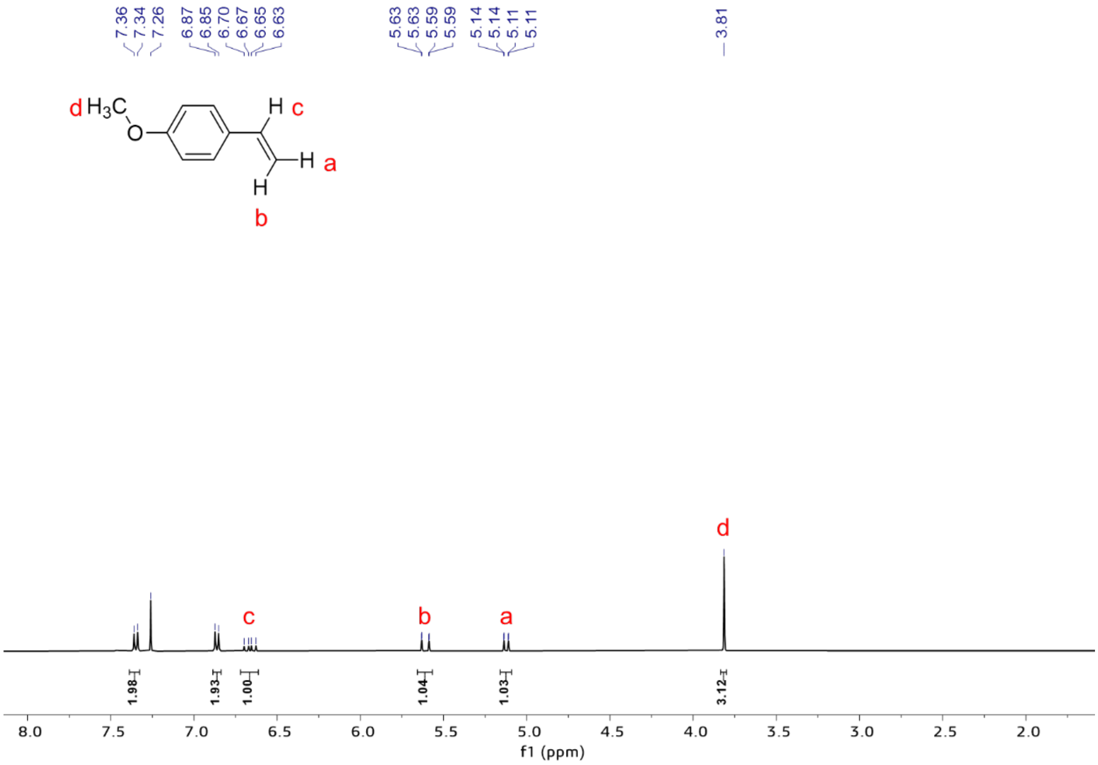

Figure S64. ^{1}H-NMR spectrum of the selective hydrogenation products of 4-methoxystyrene.

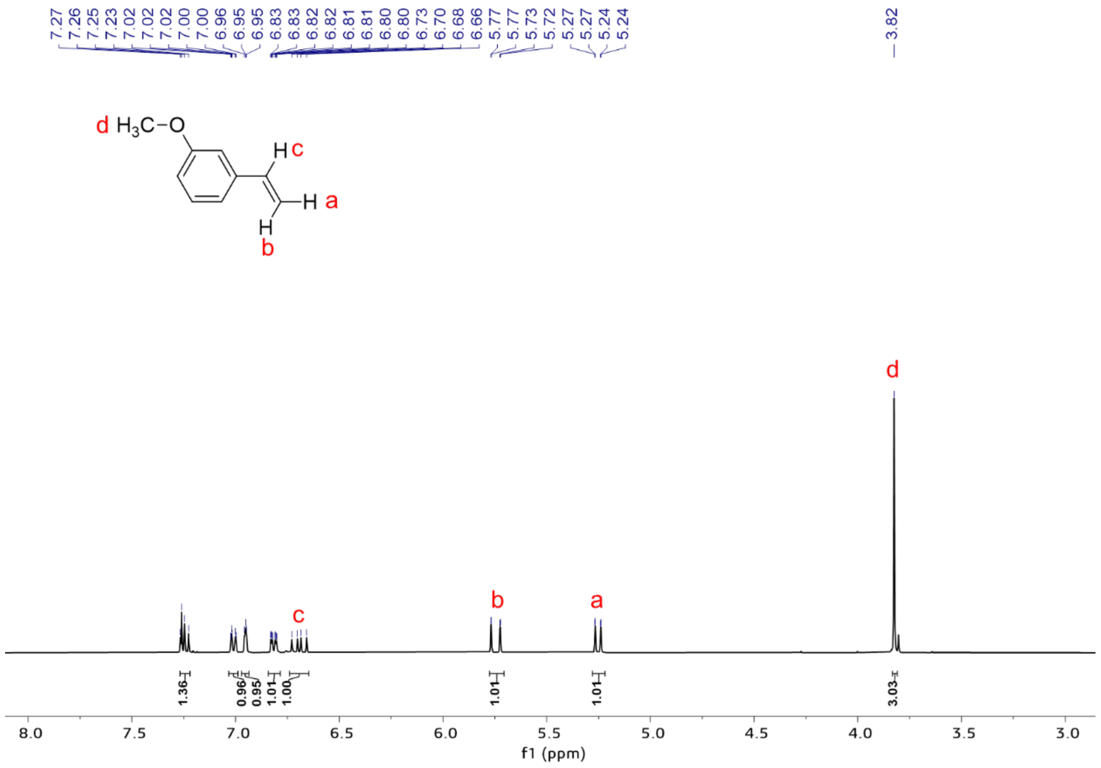

Figure S65. ^{1}H-NMR spectrum of the selective hydrogenation products of 1-methoxy-3-vinylbenzene.

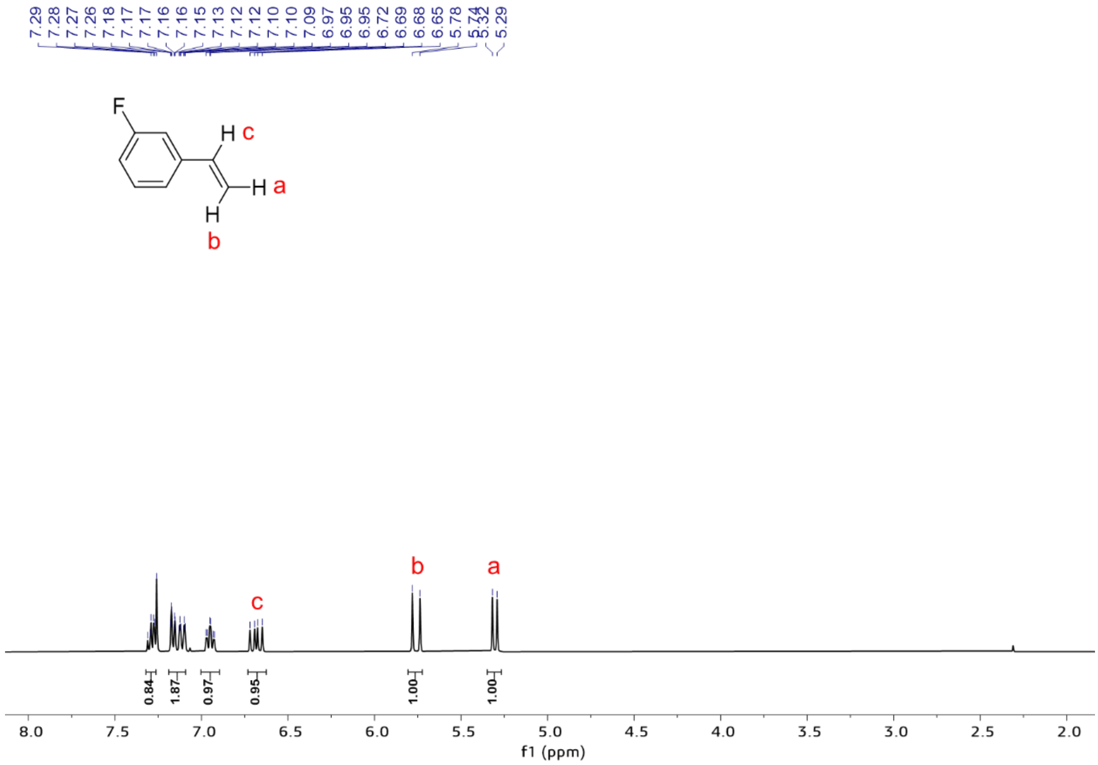

Figure S66. ^{1}H-NMR spectrum of the selective hydrogenation products of 3-fluorostyrene.

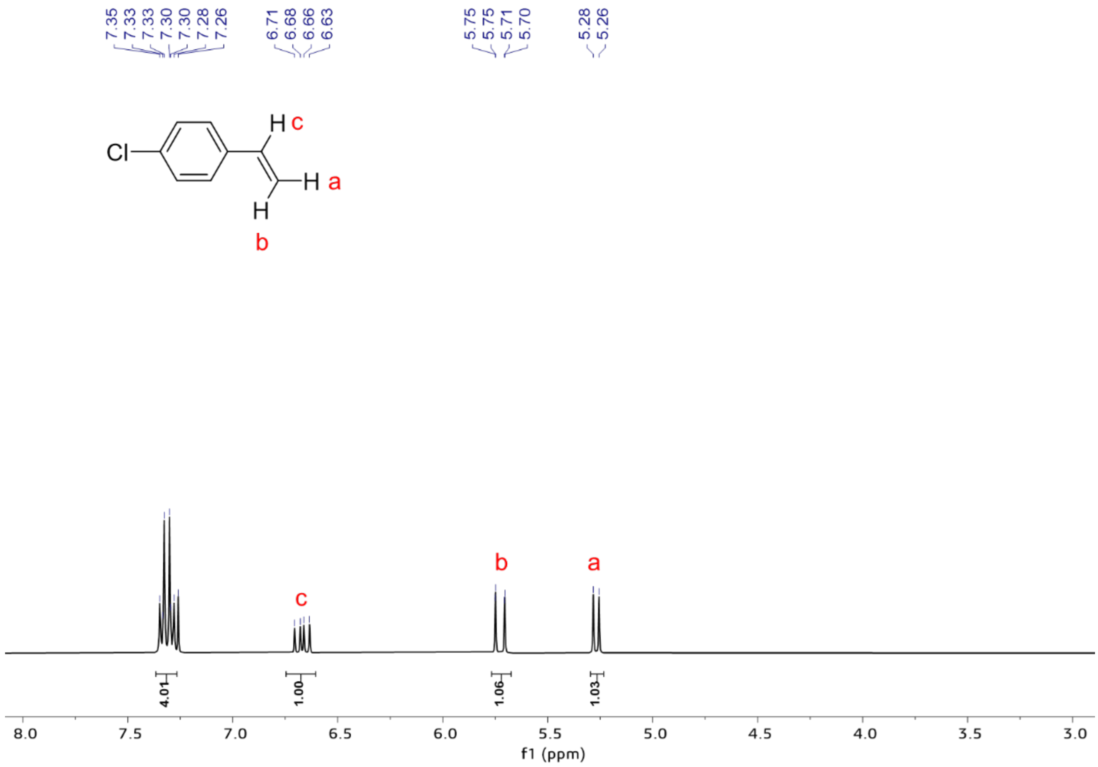

Figure S67. ^{1}H-NMR spectrum of the selective hydrogenation products of 4-chlorostyrene.

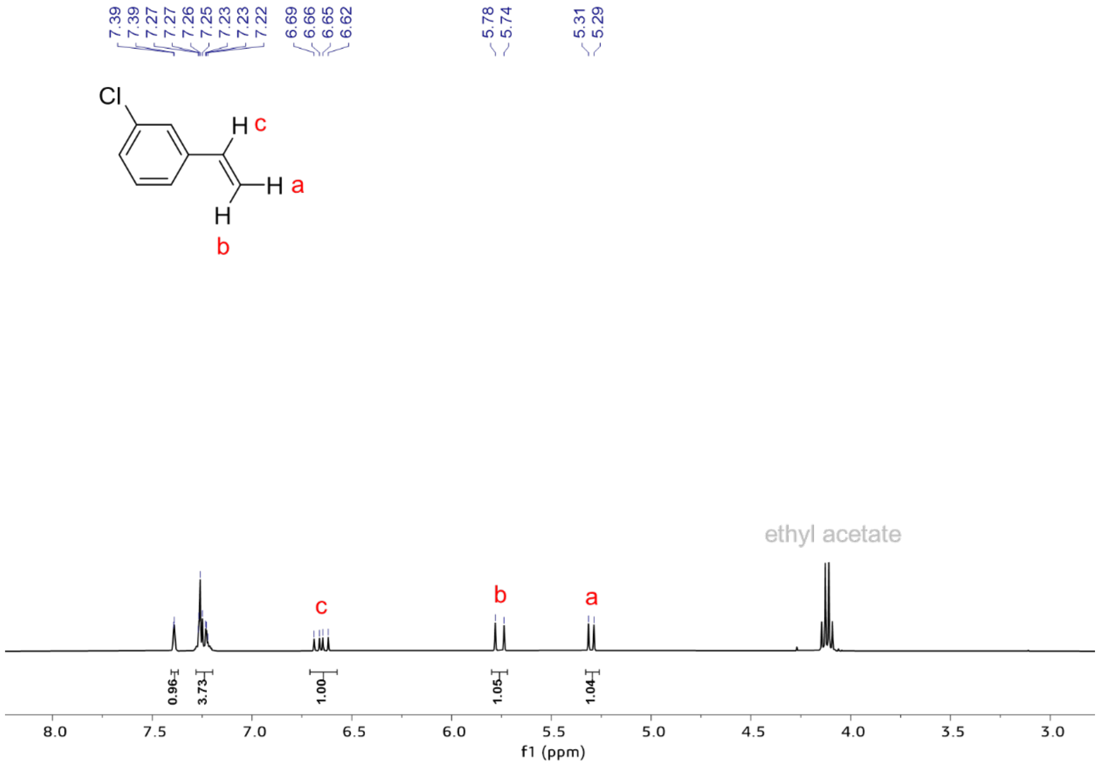

Figure S68. ^{1}H-NMR spectrum of the selective hydrogenation products of 3-chlorostyrene.

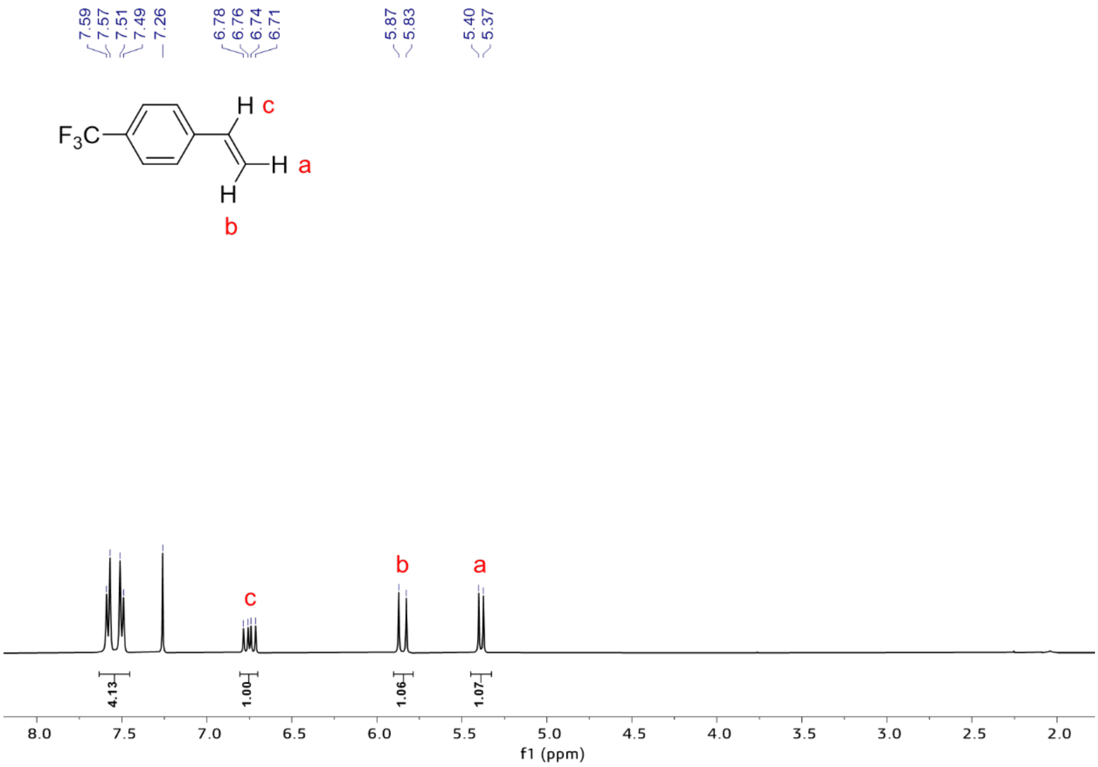

Figure S69. ^{1}H-NMR spectrum of the selective hydrogenation products of 4-(trifluoromethyl)styrene.

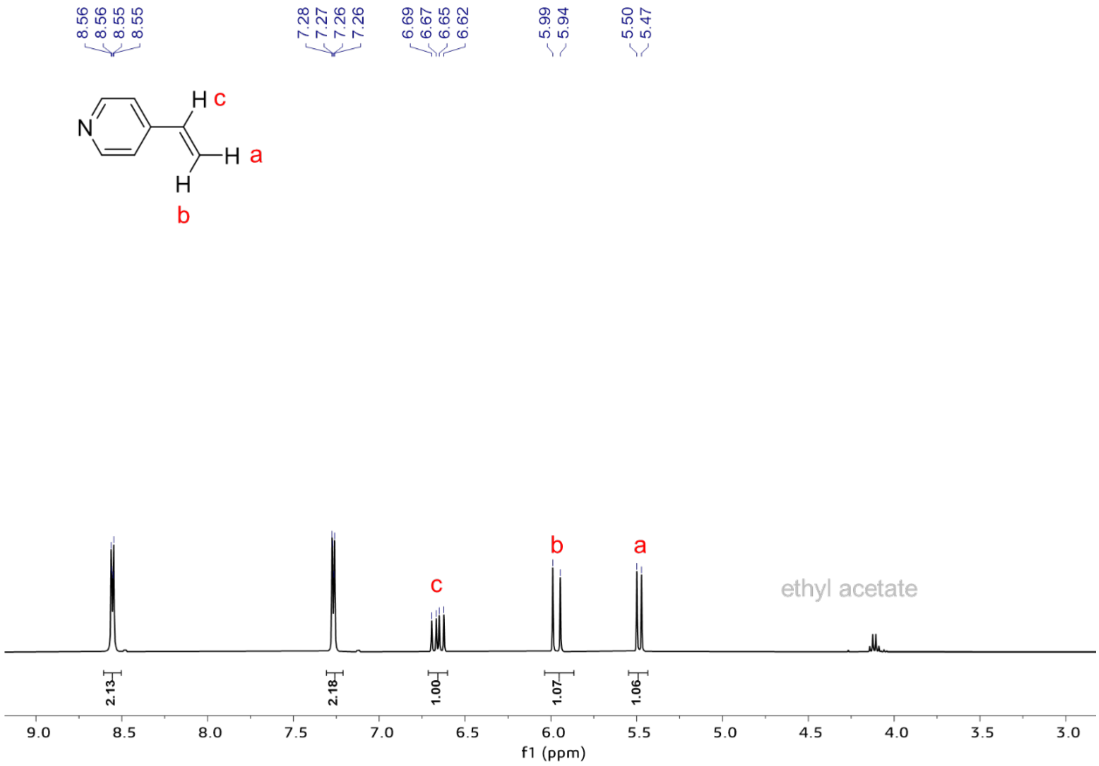

Figure S70. ^{1}H-NMR spectrum of the selective hydrogenation products of 4-vinylpyridine.

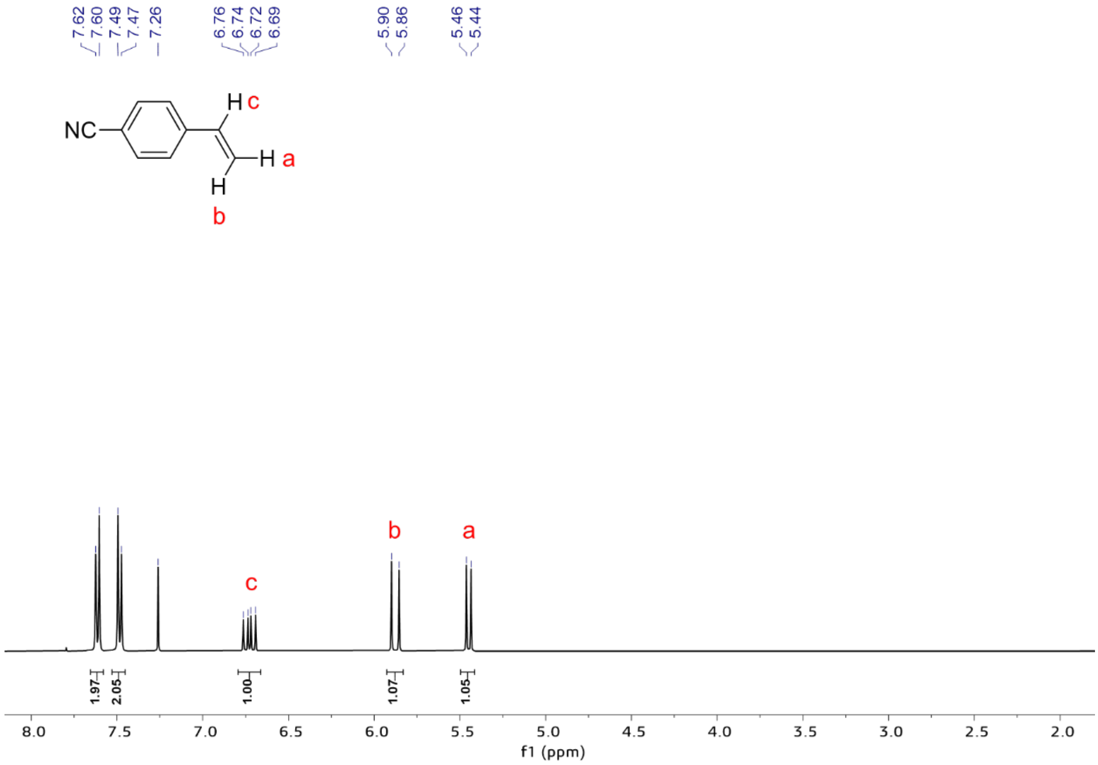

Figure S71. ^{1}H-NMR spectrum of the selective hydrogenation products of 4-cyanostyrene.

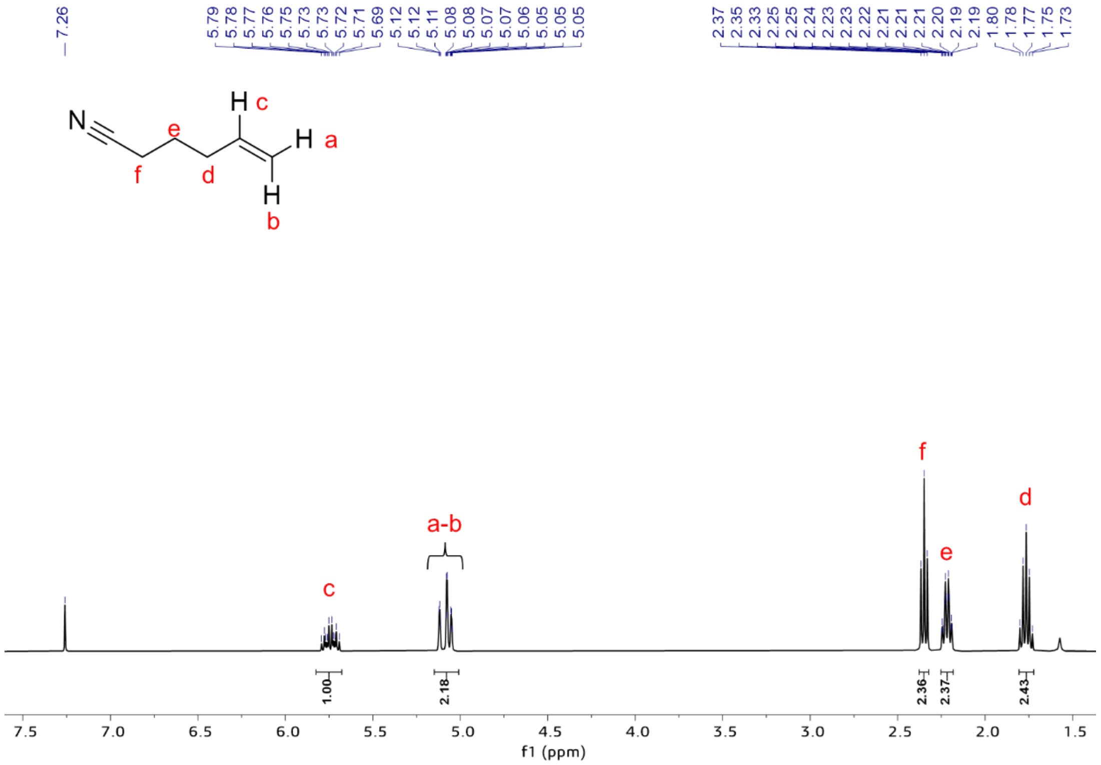

Figure S72. ^{1}H-NMR spectrum of the selective hydrogenation products of 5-hexenenitrile.

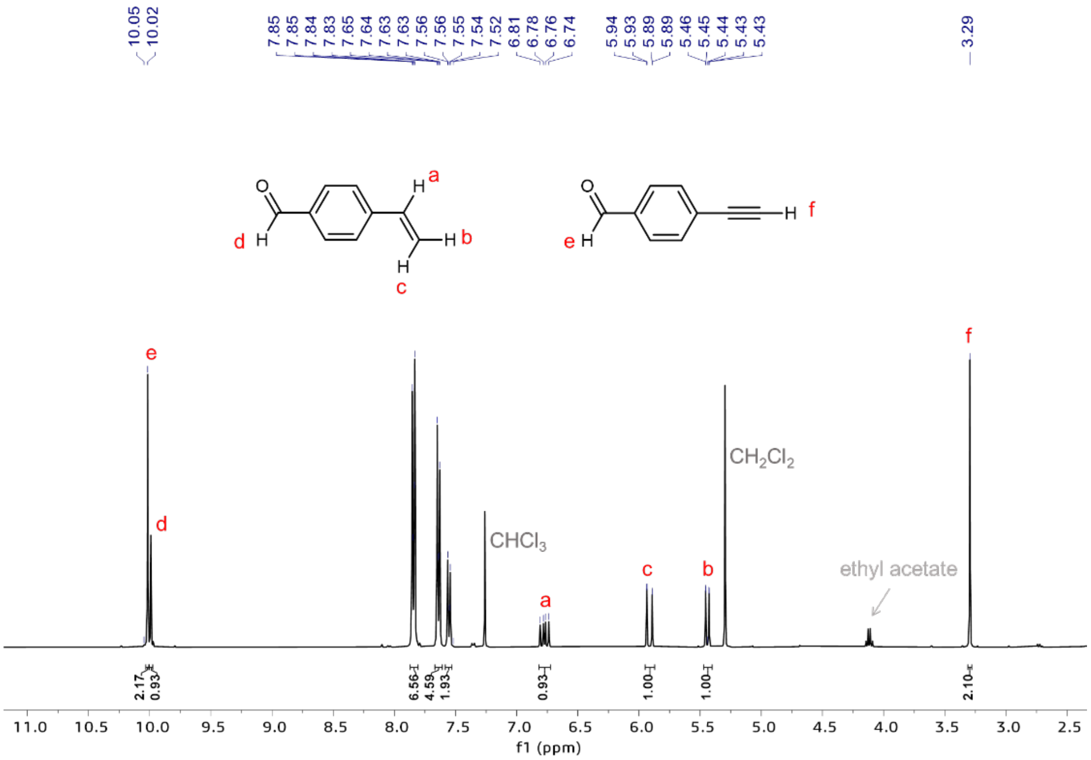

Figure S73. ^{1}H-NMR spectrum of the selective hydrogenation products of 4-ethynylbenzaldehyde.

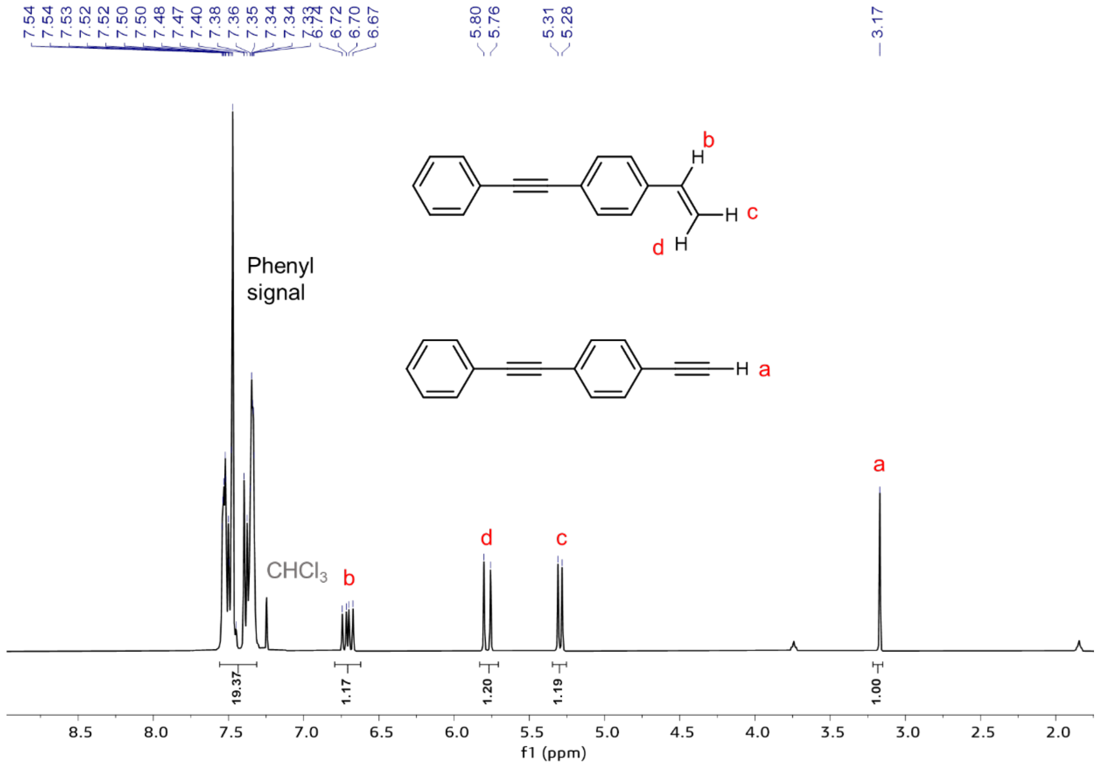

Figure S74. ^{1}H-NMR spectrum of the selective hydrogenation products of 1-ethynyl-4-(phenylethynyl)benzene.

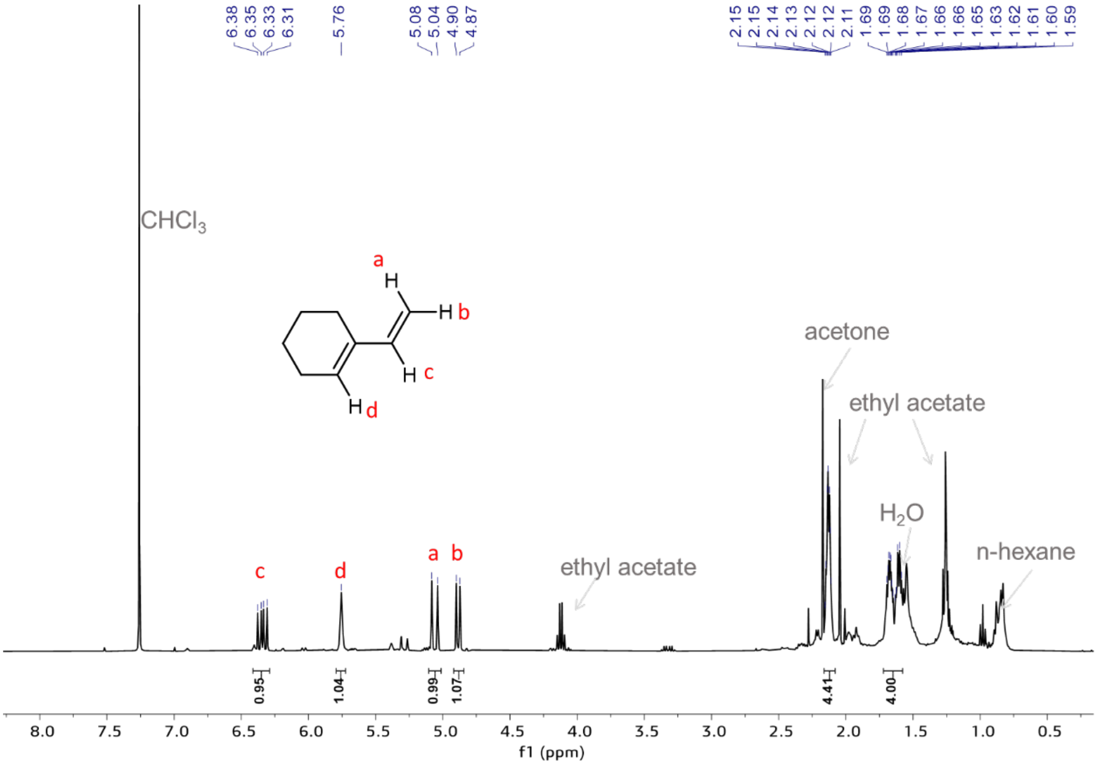

Figure S75. ^{1}H-NMR spectrum of the selective hydrogenation products of 1-vinylcyclohex-1-ene.

Supplementary Code

```
clear all;

[filename, pathname] = uigetfile('*.csv', 'select csv file');
input_file = fullfile(pathname, filename);
% If the user deselects, execution stops
if isequal(filename, 0)
  return;
end
data = readmatrix(input_file,'NumHeaderLines', 1); % Assume that the CSV file does not have a header
line

[max_x, max_idx] = max(data(:, 2));
[min_x, min_idx] = min(data(:, 2));
[max_y, max_idy] = max(data(:, 3));
[min_y, min_idy] = min(data(:, 3));
center_x = (min_x+max_x) / 2;
a = max_x-min_x;
center_y = (min_y+max_y) / 2;
b = max_y-min_y;
data([max_idx, min_idx, max_idy, min_idy], :) = [];
points_x = data(:, 2);
points_y = data(:, 3);

disp(['Analyzed particle identification: ', filename(1:end-4)]);
disp(['Center coordinates：（', num2str(center_x), ',',num2str(center_y),')']);
if a == b %spherical model
    diameter = a;
    radius = diameter / 2;
    distance = sqrt((points_x - center_x).^2 + (points_y - center_y).^2);
    filted_data = data(distance <= radius, :);
    points_x = filted_data(:, 2);
    points_y = filted_data(:, 3);
    D = 2*sqrt((points_x - center_x).^2 + (points_y - center_y).^2);
```

```
    points_z = sqrt(radius^2 - (points_x - center_x).^2 - (points_y - center_y).^2);
    disp(['diameter： ', num2str(diameter)]);
else %ellipsoidal model
    A = a/2;
    B = b/2;
    %{
    plot(points_x, points_y, 'o', 'MarkerSize', 5, 'MarkerFaceColor', 'red', 'MarkerEdgeColor', 'red');
    % Creates an Angle vector of theta that defines the range of the ellipse (0 to 2*pi)
    theta = linspace(0, 2*pi, 100);
    x_ellipse = center_x + A*cos(theta);
    y_ellipse = center_y + B*sin(theta);
    plot(x_ellipse, y_ellipse, 'r', 'LineWidth', 2);
   %}
    distance = (points_x - center_x).^2 / A^2 + (points_y - center_y).^2 / B^2;
    filted_data = data(distance <= 1, :);
    points_x = filted_data(:, 2);
    points_y = filted_data(:, 3);
    points_z = A * sqrt(1 - ((points_x - center_x).^2 / A^2) - ((points_y - center_y).^2 / B^2));
    disp(['Parameter A： ', num2str(A)]);
    disp(['Parameter B： ', num2str(B)]);
    radius = (A+B)/2;
    disp(['Mean radius:', num2str(radius), '±', num2str(abs(A-radius))]);
end

output_filename = fullfile(pathname, [filename(1:end-4), '_extended.csv']);
points = [points_x, points_y, points_z];
csvwrite(output_filename, points);

edge_threshold = 0.8;  %Set threshold value！！！

triangles = delaunay(points(:,1), points(:,2));
x = points(:,1);
y = points(:,2);
z = points(:,3);
```

```
figure;
% Find the minimum value of Z
z_min = min(z(:));
% Find the maximum value of Z
z_max = max(z(:));
%Set color mapping
cmap = colormap;
% Counts all side lengths
edge_lengths = [];
for i = 1:size(triangles, 1)
    % Gets the vertex index of the current triangle
    vertex_indices = triangles(i,:);

    % Calculates the length of the three sides
    edge1 = norm(points(vertex_indices(1), :) - points(vertex_indices(2), :));
    edge2 = norm(points(vertex_indices(2), :) - points(vertex_indices(3), :));
    edge3 = norm(points(vertex_indices(3), :) - points(vertex_indices(1), :));

    % Determines whether the side length is greater than or equal to the threshold. If so, add it to the side length list
    if edge1 <= edge_threshold
        edge_lengths = [edge_lengths, edge1];
        hold on;
        z_values = mean(points(vertex_indices([1, 2]), 3));
        color_index=(z_values-z_min)/(z_max-z_min);
        color_value=cmap(round(color_index*(size(cmap, 1)-1))+1,:);
        plot3([points(vertex_indices(1), 1), points(vertex_indices(2), 1)], [points(vertex_indices(1), 2), points(vertex_indices(2), 2)], [points(vertex_indices(1), 3), points(vertex_indices(2), 3)], 'Color', color_value);
    end

    if edge2 <= edge_threshold
        edge_lengths = [edge_lengths, edge2];
        hold on;
```

```
        z_values = mean(points(vertex_indices([2, 3]), 3));
        color_index=(z_values-z_min)/(z_max-z_min);
        color_value=cmap(round(color_index*(size(cmap, 1)-1))+1,:);
        plot3([points(vertex_indices(2), 1), points(vertex_indices(3), 1)], [points(vertex_indices(2), 2), points(vertex_indices(3), 2)], [points(vertex_indices(2), 3), points(vertex_indices(3), 3)], 'Color', color_value);
    end

    if edge3 <= edge_threshold
        edge_lengths = [edge_lengths, edge3];
        hold on;
        z_values = mean(points(vertex_indices([3, 1]), 3));
        color_index=(z_values-z_min)/(z_max-z_min);
        color_value=cmap(round(color_index*(size(cmap, 1)-1))+1,:);
        plot3([points(vertex_indices(3), 1), points(vertex_indices(1), 1)], [points(vertex_indices(3), 2), points(vertex_indices(1), 2)], [points(vertex_indices(3), 3), points(vertex_indices(1), 3)], 'Color', color_value);
    end

end

% Calculate the average side length
average_edge_length = mean(edge_lengths);
standard_deviation = std(edge_lengths);
%trimesh(triangles,x,y,z);
axis equal;
% Show results
disp(['Average length: ', num2str(average_edge_length)]);
disp(['Standard deviation: ', num2str(standard_deviation)]);

plot3(x, y, z, 'o', 'MarkerSize', 3, 'MarkerFaceColor', '#F17777', 'MarkerEdgeColor', 'none');

xlabel('Length / nm', 'FontSize', 20, 'FontName', 'Arial');
ylabel('Length / nm', 'FontSize', 20, 'FontName', 'Arial');
```

```
zlabel('Length / nm', 'FontSize', 20, 'FontName', 'Arial');
ax = gca;
set(ax, 'FontName', 'Arial', 'FontSize', 20);
set(gca, 'YDir', 'reverse');
grid off;
```

Reference

[1] B. Ravel, M. Newville, *Journal of synchrotron radiation* **2005**, *12* (4), 537.
[2] A. I. Frenkel, C. W. Hills, R. G. Nuzzo, *The Journal of Physical Chemistry B* **2001**, *105* (51), 12689, https://doi.org/10.1021/jp012769j.
[3] A. Jentys, *Phys. Chem. Chem. Phys.* **1999**, *1* (17), 4059, https://doi.org/10.1039/A904654B.
[4] B. An, Y. Meng, Z. Li, Y. Hong, T. Wang, S. Wang, J. Lin, C. Wang, S. Wan, Y. Wang, W. Lin, *J. Catal.* **2019**, *373*, 37, https://doi.org/https://doi.org/10.1016/j.jcat.2019.03.008.
[5] R. Bai, G. He, L. Li, T. Zhang, J. Li, X. Wang, X. Wang, Y. Zou, D. Mei, A. Corma, J. Yu, *Angew. Chem. Int. Ed.* **2023**, *62* (48), e202313101, https://doi.org/https://doi.org/10.1002/anie.202313101.
[6] N. Hellgren, R. T. Haasch, S. Schmidt, L. Hultman, I. Petrov, *Carbon* **2016**, *108*, 242, https://doi.org/https://doi.org/10.1016/j.carbon.2016.07.017.
